# PAIR: Reconstructing Single‐Cell Open‐Chromatin Landscapes for Transcription Factor Regulome Mapping

**DOI:** 10.1002/advs.202524392

**Published:** 2026-03-14

**Authors:** Yanchi Su, Qi Qi, Yi Fan, Yubo Wang, Gaoyang Hao, Ka‐Chun Wong, Yunhe Wang, Xiangtao Li

**Affiliations:** ^1^ School of Information Science and Technology Northeast Normal University Jilin China; ^2^ School of Artificial Intelligence Jilin University Jilin China; ^3^ Department of Computer Science City University of Hong Kong Hong Kong SAR; ^4^ School of Artificial Intelligence Hebei University of Technology Tianjin China

**Keywords:** bipartite graph neural network, clustering, imputation, scATAC‐seq

## Abstract

Single‐cell ATAC‐seq (scATAC‐seq) enables the interrogation of chromatin accessibility at cellular resolution, yet its practical utility is often constrained by limited sequencing depth, extreme sparsity, and pervasive technical missingness, which collectively hamper robust cell‐state delineation and inference of transcription factor (TF) regulatory programs. We present PAIR, a probabilistic framework that restores scATAC‐seq accessibility profiles by directly modeling the native cell–peak bipartite structure of chromatin accessibility. PAIR leverages a bipartite graph encoder to learn representations for both cells and peaks, and incorporates a variational latent layer to explicitly capture uncertainty arising from sparse and noisy measurements. To jointly recover discrete accessibility patterns and quantitative signal, PAIR integrates two complementary decoders: a qualitative decoder that reconstructs open/closed cell–peak incidences and a quantitative decoder that models accessibility counts under a Negative Binomial likelihood. Trained end‐to‐end with variational and embedding regularization, PAIR yields cell and peak embeddings and an imputed accessibility matrix that improves downstream analyses. Across simulated datasets with controlled sequencing depth, noise, and dropout, as well as multiple publicly available benchmarks, PAIR consistently improves clustering performance and increases sensitivity for differential accessibility. Beyond cell‐level analyses, PAIR‐derived peak embedding enables locus‐centric regulatory interrogation: co‐accessibility analysis around SOX10 reveals structured regulatory neighborhoods, and graph‐based peak modules show selective activity across melanoma cell states and identify gene sets with clinically relevant survival associations. In a forebrain atlas, PAIR restores regulatory signals spanning both promoter‐proximal and distal elements and uncovers biologically coherent enrichment patterns consistent with neuronal specialization.

## Graphical abstract

scATAC‐seq analysis is often constrained by limited sequencing depth, extreme sparsity, and pervasive technical missingness. PAIR is a probabilistic framework that restores scATAC‐seq accessibility profiles by directly modeling the native cell–peak bipartite structure of chromatin accessibility. This model improves robustness for downstream analyses such as clustering, batch‐aware integration, and regulatory program discovery.

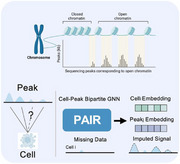

## 1 Introduction

Single‐cell epigenomics enables the systematic characterization of regulatory landscapes at cellular resolution, offering a direct window into how chromatin state underpins gene regulation, cell identity, and intra‐tissue heterogeneity. Among these technologies, single‐cell ATAC‐seq (scATAC‐seq) [1, 2] profiles chromatin accessibility by capturing Tn5 insertion events across the genome in individual cells. By resolving accessibility variation across cells, scATAC‐seq enables the identification of cell types and states [3] from their regulatory programs, supports the systematic discovery of candidate cis‐regulatory elements [4, 5] and their cell‐type‐specific activity, and facilitates the inference of putative transcriptional regulators through motif enrichment and related analyses [6].

In practice, however, scATAC‐seq measurements are dominated by extreme sparsity and high dimensionality: although each cell contains a large number of potentially accessible loci, only a small number of distinct fragments are sequenced, yielding pervasive dropouts and a data structure that is often close to binary at the peak level [7]. A wide range of denoising and imputation strategies has been developed for scRNA‐seq [8, 9, 10, 11], and some have been applied to scATAC‐seq by analogy [12]. Yet scATAC‐seq differs fundamentally in measurement physics and data geometry, so direct transfer often produces unstable or suboptimal results. This gap has motivated scATAC‐specific methods that learn low‐dimensional representations for visualization, clustering, and downstream regulatory inference, while contending with sparsity and technical confounders.

Importantly, many existing approaches can be grouped according to the type of representation they emphasize, either qualitative accessibility states (binary open/closed) or quantitative intensity signals (count‐aware). This modeling choice directly determines which aspects of chromatin accessibility are preserved in downstream analyses. On the qualitative side, several workflows explicitly operate on binarized accessibility. For example, SnapATAC [13] represents each cell as a binary vector over genome bins and constructs a Jaccard similarity matrix for downstream embedding; batch effects are addressed by applying Harmony in the reduced space. cisTopic [14] analyses are also frequently presented in terms of a binary accessibility matrix when assessing reconstructed accessibility patterns. Similarly, SCALE [15] combines a variational autoencoder with a probabilistic Gaussian mixture model to learn latent features tailored to scATAC‐seq and support unsupervised clustering. scOpen [16] likewise starts from a binarized scATAC‐seq cell‐by‐region matrix, applies TF–IDF (term frequency–inverse document frequency) weighting, and then performs regularized Non‐negative matrix factorization (NMF) to obtain denoised accessibility estimates. ArchR [17] adopts a TF–IDF and latent semantic indexing (LSI) workflow similar to related pipelines. Crucially, its iterative LSI implementation applies binarization by default. This choice prioritizes presence or absence signals over count magnitude when modeling insertion profiles.

In contrast, a second line of methods retains more of the count structure or uses transformations defined on counts to preserve quantitative variation. In Signac [18], the standard workflow performs TF–IDF normalization (correcting across cells and reweighting peaks) followed by SVD on the TF–IDF matrix to obtain low‐dimensional embeddings for clustering and visualization. SnapATAC2 [19] performs spectral embedding by projecting a cell‐by‐feature count matrix into a low‐dimensional space. It supports similarity metrics based on cosine distance or the Jaccard index. These options provide a practical connection between count‐weighted representations and set‐overlap views of accessibility. Importantly, when multiple fragments support the same peak in a given cell, the count magnitude provides a natural notion of evidence strength for accessibility rather than merely presence/absence. This quantitative variation can help distinguish strongly accessible regulatory elements from weak or sporadic accessibility, and it becomes especially informative when aggregating signals across groups of cells for differential accessibility or regulatory program inference [7], where subtle but consistent shifts in accessibility intensity may be missed after binarization. Finally, probabilistic frameworks such as PeakVI [20] explicitly allow the input accessibility matrix to be binary or count‐valued, and incorporate observed covariates to reduce technical effects in the learned representation. This landscape highlights a recurring trade‐off: binary formulations can be robust to dropouts and often integrate naturally with similarity measures and batch correction in latent space, but they may discard potentially informative variation in accessibility magnitude; count‐aware formulations preserve more quantitative structure, yet can be more sensitive to coverage heterogeneity and technical biases. Framing prior work through this qualitative–quantitative lens makes it easier to motivate “dual‐view” models that aim to exploit both types of signal rather than committing to only one.

In parallel to deep generative models, a growing line of work [21, 22, 23, 24, 25] uses graph neural networks (GNNs) for representation learning in omics, where message passing provides a principled way to aggregate information along explicitly defined relationships. Several recent methods illustrate how graph construction and self‐supervised objectives can improve robustness and capture higher‐order dependencies beyond feature‐only modeling. For example, SpaBatch [21] demonstrates how a variational graph autoencoder can be paired with self‐supervised and metric‐learning objectives to support integration across datasets while mitigating batch effects, highlighting the usefulness of graph‐based latent modeling for aligning heterogeneous measurements. SpaCross [22] combines masked self‐supervision with hybrid graph modeling for cross‐dataset integration, reinforcing the broader trend that carefully designed graph objectives can substantially influence the learned representations. These approaches differ in the specific relationships they encode, for example, graphs based on neighborhood structure, feature similarity, or cross‐view consistency, but collectively they highlight a general lesson: explicitly modeling relational structure can be beneficial when the data exhibit strong sparsity, heterogeneity, or multi‐level organization.

Here, we present PAIR, a probabilistic framework for scATAC‐seq that models chromatin accessibility through a cell–peak bipartite graph, where cells and peaks are represented as two distinct node types and edges encode open/closed accessibility states. PAIR couples a variational latent layer with a dual‐decoder design to jointly capture complementary aspects of the data: a qualitative decoder reconstructs the binary incidence structure of accessibility, while a quantitative decoder models over‐dispersed accessibility counts using a Negative Binomial likelihood. To mitigate major technical confounders, PAIR explicitly accounts for cell‐specific sequencing depth and peak‐specific baseline effects, and can be applied in settings with substantial batch‐driven variation, yielding representations that are less dominated by these nuisance factors. The learned representations operate at two levels: (i) uncertainty‐aware low‐dimensional embeddings for cells and peaks that support downstream analyses such as integration, subpopulation discovery, and visualization; and (ii) a probabilistic, corrected reconstruction of the accessibility matrix that enables more reliable inference of differential accessibility and cell‐state annotation at single‐region resolution. We demonstrate PAIR on multiple published datasets and benchmark it against state‐of‐the‐art methods across clustering, imputation, and regulatory analyses, showing that PAIR improves cell‐state resolution and facilitates transcription factor–centric interpretation of regulatory programs from sparse scATAC‐seq profiles.

## 2 Results

### 2.1 Overview of the Method

scATAC‐seq profiles are intrinsically sparse due to limited fragments per cell, leading to widespread missingness and substantial technical variability in observed accessibility counts. PAIR addresses this challenge by modeling scATAC‐seq data through a cell–peak bipartite graph and a probabilistic dual‐decoder framework that jointly captures (i) the qualitative open/closed accessibility structure and (ii) the quantitative count distribution. Starting from the raw cell‐by‐peak count matrix, PAIR performs standard quality control to filter low‐quality cells and low‐prevalence peaks, computes a cell‐wise library size factor to account for depth heterogeneity, and binarizes the matrix to construct the cell–peak adjacency, where an edge indicates that a peak is accessible in a cell). On this bipartite graph, PAIR applies a simplified propagation‐based bipartite graph encoder that iteratively aggregates information between cells and peaks using normalized neighborhood aggregation across multiple layers, following the principle of retaining the core message‐passing component while avoiding unnecessary transformations. To explicitly represent uncertainty under severe sparsity, the encoder output is passed through a variational latent layer that parameterizes a node‐wise Gaussian posterior for both cell and peak nodes and samples stochastic embeddings via the reparameterization trick; the posterior is regularized toward a standard normal prior via a Kullback–Leibler (KL) divergence. Finally, PAIR employs two complementary decoders: a qualitative decoder that reconstructs the binary cell–peak accessibility structure (open/closed) from cell–peak latent compatibility, and a quantitative decoder that reconstructs the observed counts under a Negative Binomial (NB) likelihood, whose mean is modulated by cell depth and peak‐specific effects to model over‐dispersed single‐cell count variability. By jointly optimizing the qualitative structural objective, the quantitative NB reconstruction objective, and variational regularization, PAIR outputs (1) uncertainty‐aware cell and peak embeddings and (2) an imputed accessibility signal, enabling downstream analyses such as clustering, batch correction, and transcription regulatory program inference from denoised chromatin accessibility landscapes.

### 2.2 Simulation Benchmarks Demonstrate Robust scATAC‐seq Imputation by PAIR Across Sparsity, Depth, and Noise

We evaluated imputation performance under controlled ground truth using two complementary simulation settings. First, we generated fully synthetic scATAC‐seq datasets in silico, in which both the underlying accessibility state and intensity for each cell–peak pair are explicitly specified, enabling a direct assessment of reconstruction accuracy under increasing sparsity. Second, we constructed bulk‐guided simulated datasets to model factors that arise from experimental sampling, including reduced sequencing depth and additive noise.

**FIGURE 1:**
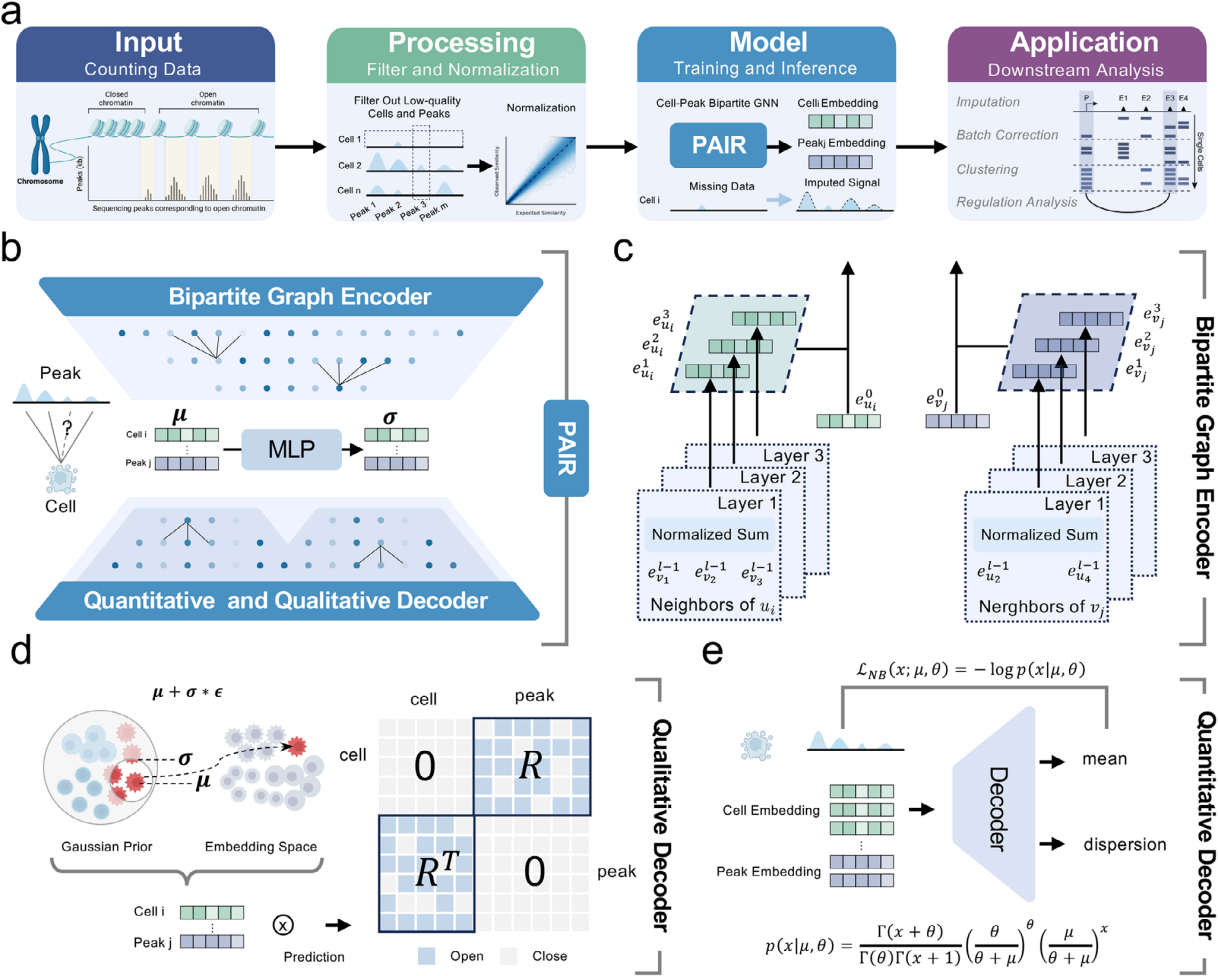
Overview of PAIR for scATAC‐seq imputation, clustering, and downstream analysis. (a) End‐to‐end workflow. Starting from the raw cell‐by‐peak scATAC‐seq count matrix, low‐quality cells and peaks are filtered, and the data are normalized. PAIR is then trained on the resulting cell–peak bipartite graph to produce cell and peak embeddings and an imputed accessibility signal, which can be used for downstream tasks such as imputation, batch correction, clustering, and regulatory analysis. (b) Model architecture. A bipartite graph encoder learns representations for both cell and peak nodes; a variational module parameterizes a node‐wise Gaussian posterior with mean μ and standard deviation σ (predicted by an MLP), bridging the encoder and the two decoders. (c) Bipartite graph message passing. Cell and peak embeddings are updated by aggregating information from opposite‐type neighbors via normalized summation across multiple propagation layers; embeddings from different layers capture increasingly high‐order neighborhood structure and are combined to form final representations. (d) Qualitative decoder. Latent embeddings are sampled via the reparameterization trick and used to reconstruct the binary cell–peak adjacency (open/closed accessibility) through a compatibility score between cell and peak embeddings. (e) Quantitative decoder. The count matrix is reconstructed under a Negative Binomial likelihood, where the decoder outputs distribution parameters (mean and dispersion) to model over‐dispersed scATAC‐seq counts.

**FIGURE 2:**
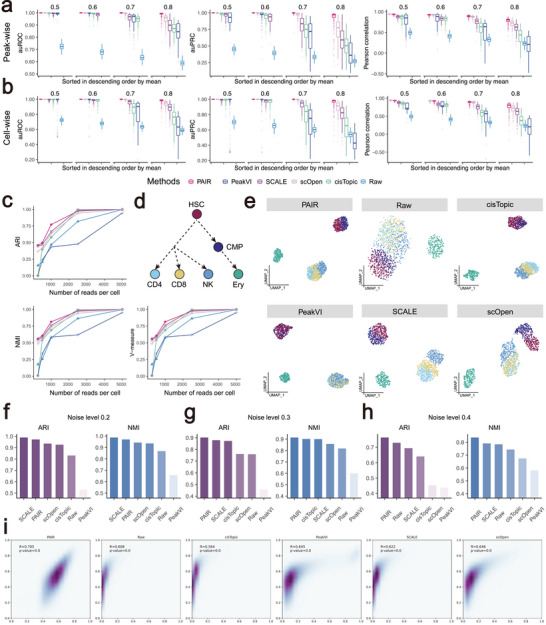
Simulation benchmarks demonstrate robust scATAC‐seq imputation by PAIR across sparsity, depth, and noise. (a‐b) Performance on simulated datasets with increasing sparsity (retention rates 0.5–0.8). Imputation quality is assessed by auROC, auPRC, and Pearson correlation, comparing imputed signals with the bulk‐derived reference. Boxplots summarize results across simulated replicates, and methods are ordered by their mean performance. (c) Performance under varying sequencing depth, generated by downsampling to different numbers of reads per cell. Clustering quality on the imputed data is evaluated using ARI, NMI, and V‐measure. (d) Ground‐truth developmental relationships among cell types used in the simulation, reflecting a hematopoietic hierarchy from HSC to CMP and downstream lineages (CD4, CD8, NK, and Ery). (e) UMAP visualization at 1,000 reads per cell, comparing PAIR with competing methods and the raw input. (f–h) Robustness of imputation to noise. Bar plots report ARI and NMI at noise levels 0.2 (e), 0.3 (f), and 0.4 (g). (i) Agreement between imputed profiles and the bulk reference, shown as density scatter plots. Pearson correlation coefficients and corresponding p‐values are reported for each method.

We first increased sparsity from 0.5 to 0.8 by progressively masking accessibility observations and then quantified imputation accuracy using both peak‐wise and cell‐wise metrics (Figures 1 and 2a,b), including auROC and auPRC for recovering accessible events and Pearson correlation for agreement with the simulated quantitative signal. Across all sparsity settings, PAIR shows the best overall performance by mean auROC, auPRC, and Pearson correlation, with the advantage becoming most pronounced under severe sparsity (0.7–0.8). When sparsity is mild (0.5–0.6), most methods perform comparably, whereas at higher sparsity levels competing methods exhibit a clear decline in auPRC and correlation. In contrast, PAIR maintains higher precision–recall performance and stronger quantitative concordance, indicating improved recovery of both the qualitative accessibility pattern and the underlying intensity structure under extensive missingness. As expected, the raw matrix consistently yields the lowest auROC/auPRC and reduced correlations, demonstrating that increased sparsity substantially erodes signal and that accurate recovery requires principled modeling.

We next downsampled fragments to generate datasets with varying numbers of reads per cell (250, 500, 1000, 2500, 5000) and quantified downstream clustering quality using ARI, NMI, completeness, Homogeneity, and V‐measure (Figure 2c; Figure S2c,d). All methods improve as depth increases and converge at high coverage, but PAIR shows a clear advantage in the low‐depth regime where scATAC‐seq is most challenging. At 1,000 reads per cell, PAIR consistently attains higher metircs than PeakVI, SCALE, scOpen, and cisTopic, and substantially outperforms the raw data. PeakVI exhibits the slowest improvement as depth increases, whereas PAIR reaches near‐saturated clustering accuracy with fewer reads, indicating that it extracts stable cell‐state structure more efficiently under limited sampling. Consistent with the clustering metrics, UMAP embeddings at 1,000 reads per cell (Figure 2e) indicate that PAIR recovers a clear separation of major lineages (Figure 2d) and improves resolution among closely related cell states. In the PAIR embedding, the progenitor compartment forms a compact cluster in which HSC and CMP occupy distinct subregions, whereas PeakVI, SCALE, and cisTopic show noticeably stronger mixing between these two progenitor states. PAIR also yields a more structured separation between CD4 and CD8 T cells, which appear as partially resolved subclusters rather than a single heavily overlapping mass. By comparison, the raw matrix produces a collapsed manifold with diffuse boundaries, most prominently reflected by substantial overlap between CD4 and CD8 populations and reduced separation between progenitors and differentiated lineages. scOpen separates the progenitor states more clearly than most baselines, but it still shows appreciable CD4–CD8 overlap, suggesting less stable neighborhood geometry. UMAP plots at other sequencing depth are shown in Figures S1 and S2. Overall, PAIR more consistently preserves inter‐lineage separation while maintaining within‐lineage compactness across the simulated populations.

To stress‐test robustness, we injected additional noise and evaluated clustering after imputation (Figure 2f–h). At noise level 0.2, SCALE achieves the highest ARI and NMI, with PAIR a close second and both substantially ahead of Raw and PeakVI. As noise increases, PAIR becomes the most robust approach. At noise level 0.3, PAIR achieves the best ARI and NMI, exceeding SCALE and cisTopic, and maintaining a large margin over scOpen, Raw, and PeakVI. At noise level 0.4, PAIR remains the top performer, while Raw and SCALE drop to lower values and scOpen and PeakVI degrade most severely. These results indicate that PAIR preserves discriminative structure under perturbations that mimic technical variability and spurious insertions. Finally, we assessed global concordance between imputed profiles and the bulk reference using correlation analysis (Figure 2i). PAIR achieves the highest Pearson correlation (R = 0.705), outperforming scOpen (R = 0.646), PeakVI (R = 0.645), SCALE (R = 0.622), Raw (R = 0.608), and cisTopic (R = 0.564). The tighter concentration of points along the diagonal further indicates that PAIR better recovers bulk‐consistent quantitative trends rather than only improving qualitative separability.

Overall, across controlled increases in sparsity, decreases in sequencing depth, and injected noise, PAIR provides the most reliable reconstruction and the greatest improvements in downstream clustering. The benefits are especially evident in the regimes that most closely resemble real scATAC‐seq data, namely, high sparsity and limited reads per cell.

### 2.3 PAIR Improves Cell Clustering Across Diverse scATAC‐seq Datasets

We next assessed whether the representations learned by PAIR translate into improved cell clustering on real scATAC‐seq datasets spanning distinct tissues and experimental settings. We compared PAIR with cisTopic, EpiScanpy, PeakVI, SCALE, scDEC, and SnapATAC2 on nine public benchmarks, using the same clustering protocol and evaluating agreement with provided cell‐type annotations. In addition to ARI and NMI, we reported homogeneity, completeness, and V‐measure to account for differences in cluster granularity across methods.

**FIGURE 3:**
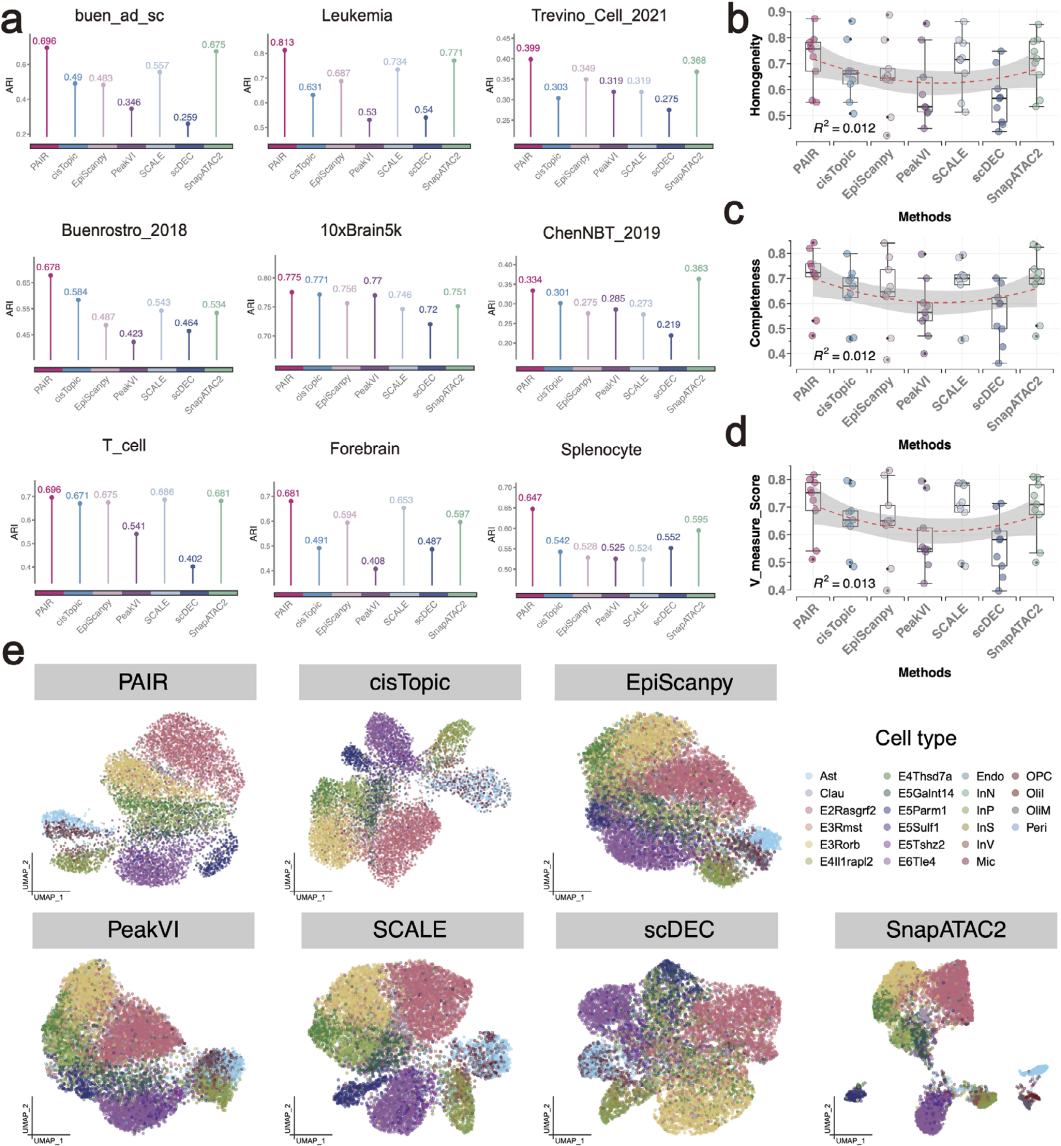
PAIR improves cell clustering across diverse scATAC‐seq datasets. (a) Clustering accuracy measured by ARI on nine scATAC‐seq datasets. Bars indicate ARI for each method, and the numeric value above each bar reports the corresponding ARI. Methods compared include PAIR, cisTopic, EpiScanpy, PeakVI, SCALE, scDEC, and SnapATAC2. (b–d) Cross‐dataset distributions of clustering quality measured by homogeneity (b), completeness (c), and V‐measure (d). Each point corresponds to one dataset; boxplots summarize variability across datasets for each method. (e) UMAP visualization on the ChenNBT_2019 dataset generated from method‐specific representations. Cells are colored by annotated cell type.

Across datasets, PAIR achieved the highest clustering accuracy in 8 of 9 benchmarks (Figure 3a). The gains were particularly evident in datasets with pronounced heterogeneity. For example, on the Leukemia dataset, PAIR reached an ARI of 0.813, exceeding SnapATAC2 (0.771) and SCALE (0.734), while PeakVI and scDEC remained substantially lower. Similar improvements were observed on buen_ad_sc (PAIR 0.696 vs. cisTopic 0.490 and PeakVI 0.346) and Forebrain (PAIR 0.681 vs. SCALE 0.653 and SnapATAC2 0.597). On Trevino_Cell_2021, which is overall more challenging, PAIR still provided the top ARI (0.399), with the next best method being SnapATAC2 (0.368). The only exception was ChenNBT_2019, where SnapATAC2 achieved the highest ARI (0.363) and PAIR ranked second (0.334), remaining better than the other baselines. The advantage of PAIR was consistent when measured by complementary clustering metrics. Over all datasets, PAIR showed the highest median homogeneity, completeness, and V‐measure (Figure 3b–d), indicating that its clusters are simultaneously purer with respect to cell labels and more complete in capturing each annotated population. Competing methods exhibited larger variability across datasets, with PeakVI and scDEC showing the lowest overall consistency.

We further visualized the learned manifolds using the ChenNBT dataset (Figure 3e). PAIR produced a coherent embedding with compact group structure and clear separation between major cellular compartments. In contrast, EpiScanpy and PeakVI displayed broader overlap among several cell types, suggesting less stable neighborhood geometry. SnapATAC2 separated some populations strongly but yielded a more fragmented embedding with multiple disconnected components. cisTopic and scDEC produced manifolds with increased mixing for several annotated subtypes. These qualitative observations are consistent with the quantitative clustering results in Figure 3a–d. In the Forebrain dataset (Figure S7c), the dashed region highlights a particularly challenging neighborhood dominated by excitatory neuronal subtypes (EX1/EX2/EX3), where high regulatory similarity and extreme sparsity often lead to subtype collapse and local mixing in low‐dimensional embeddings. PAIR produces a markedly cleaner organization in this area: EX1, EX2, and EX3 occupy a continuous yet clearly stratified manifold with sharper subtype boundaries, while remaining well separated from surrounding glial populations (AC/MG/OC) and inhibitory neurons (IN1/IN2). This indicates that PAIR enhances the usable chromatin accessibility signal without over‐smoothing subtype‐specific regulatory differences, thereby preserving meaningful local neighborhood structure. In contrast, cisTopic and EpiScanpy show pronounced color intermixing within the circled region, suggesting insufficient resolution of closely related excitatory programs under sparse coverage. PeakVI improves global separation but still exhibits substantial within‐region blending, implying limited discrimination among fine excitatory subtypes. SCALE and scDEC display stronger embedding biases in this neighborhood, such as uneven compaction and elongation and mixed boundaries, consistent with less stable modeling of local structure. SnapATAC2 tends to fragment cells into discrete islands, reducing continuity and obscuring potential gradations among excitatory states. Collectively, the circled‐region comparison demonstrates that PAIR better mitigates sparsity‐driven subtype mixing while avoiding over‐fragmentation, leading to more faithful clustering and finer‐grained excitatory subtype delineation in Forebrain scATAC‐seq data. The other UMAP plots of benchmarks are shown in Figures S5–S7.

### 2.4 PAIR Achieves Balanced Batch Correction while Preserving Biological Structure

To assess batch correction on real scATAC‐seq data, we evaluated PAIR on a multi‐batch hematopoietic dataset comprising multiple bone marrow donors (batches BM0106, BM0828, BM1077, BM1137, and BM1214) [26]. We compared PAIR with SCALE, cisTopic, SnapATAC2, PeakVI, EpiScanpy, and scDEC, as well as the unintegrated baseline. All methods were assessed on the same downstream pipeline, and integration quality was quantified using a standard set of complementary metrics that separately measure biological conservation and batch mixing.

**FIGURE 4:**
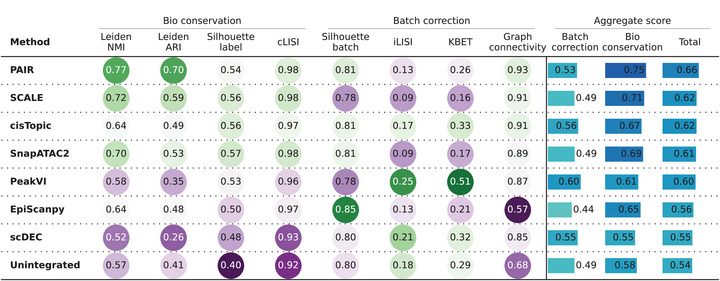
PAIR achieves balanced batch correction while preserving biological structure. Quantitative evaluation of integration quality across methods. Metrics are grouped into biological conservation (Leiden NMI, Leiden ARI, silhouette label, cLISI) and batch correction (silhouette batch, iLISI, kBET, graph connectivity). Rightmost columns report aggregated batch correction, biological conservation, and total scores.

Across metrics capturing biological structure (Figure 4a, left), PAIR achieved the strongest overall performance. It obtained the highest Leiden‐based clustering agreement with cell‐type annotations (Leiden NMI 0.77, Leiden ARI 0.70) and a high cell‐type silhouette score (0.54), indicating that the integrated embedding retains clear separation among hematopoietic populations. PAIR also achieved a near‐optimal cLISI (0.98), supporting strong local consistency of biological labels after integration. By comparison, SCALE and SnapATAC2 preserved biological structure reasonably well but with lower clustering agreement (Leiden ARI 0.59 and 0.53, respectively), while PeakVI and scDEC exhibited substantially weaker label preservation (Leiden ARI 0.35 and 0.26).

For batch correction metrics (Figure 4a, middle), PAIR achieved competitive batch mixing while maintaining stable neighborhood connectivity. In particular, PAIR showed high graph connectivity (0.93) and a favorable batch‐related silhouette (0.81), indicating that cells remain well connected in the integrated space without forming batch‐isolated components. Some methods achieved stronger batch removal in aggregate (PeakVI batch correction score 0.60; cisTopic 0.56) but at the cost of reduced biological conservation (bio conservation 0.61 for PeakVI and 0.67 for cisTopic). PAIR provided the best overall balance between the two objectives, yielding the highest total score (0.66) among all methods (Figure 4a, right). Relative to the unintegrated baseline (total 0.54), PAIR substantially improved both batch mixing and label preservation simultaneously.

**FIGURE 5:**
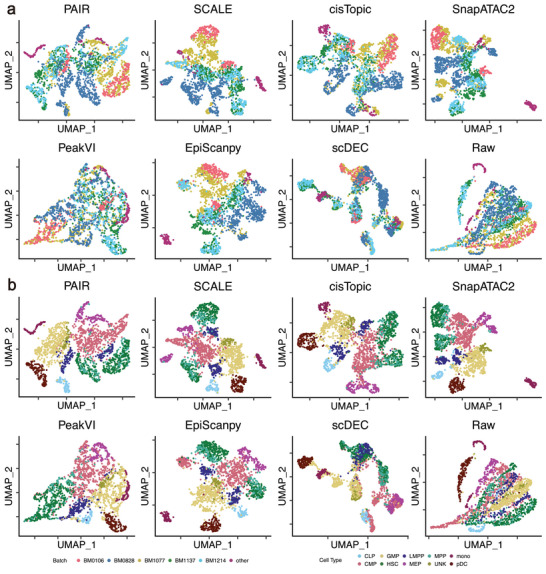
PAIR achieves balanced batch correction while preserving biological structure. (a) UMAP visualization of method‐specific embeddings colored by batch (donors BM0106, BM0828, BM1077, BM1137, BM1214, and others). (b) The same UMAP embeddings colored by cell type (CLP, CMP, GMP, HSC, LMPP, MPP, MEP, monocytes, pDC, and unknown). Methods compared include PAIR (ours), SCALE, cisTopic, SnapATAC2, PeakVI, EpiScanpy, scDEC, and the unintegrated/raw baseline.

These quantitative trends are consistent with the UMAP visualizations. When colored by batch (Figure 5a), the PAIR embedding shows strong intermixing of donor‐specific cells across the manifold rather than batch‐driven segregation. When colored by cell type (Figure 5b), PAIR maintains coherent and well‐separated hematopoietic populations, whereas methods that more aggressively mix batches tend to blur boundaries between nearby lineages, and the raw embedding exhibits prominent batch‐associated structure.

### 2.5 PAIR Reveals Co‐Accessible Regulatory Modules in Melanoma scATAC‐seq

To demonstrate that PAIR's peak‐level representation can support regulatory‐network interpretation beyond cell clustering, we analyzed a melanoma scATAC‐seq dataset [14], which includes melanoma cell states and SOX10 perturbation/time‐course measurements. We computed co‐accessible peak relationships using PAIR‐derived peak features.

**FIGURE 6:**
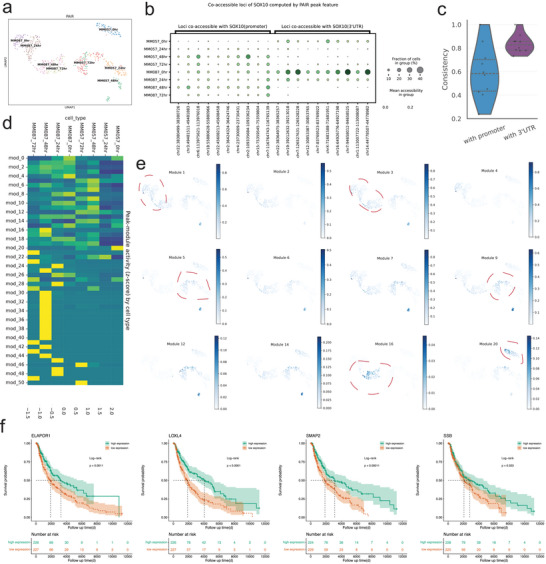
PAIR reveals co‐accessible regulatory modules in melanoma scATAC‐seq. (a) UMAP embedding of melanoma single cells computed from PAIR‐imputed peak features, colored by cell state (time‐point/condition labels as indicated). (b) Dot plot summarizing loci co‐accessible with the SOX10 promoter (left) or SOX10 3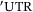 (right) across cell states. Dot size indicates the fraction of cells in each group with accessibility at the locus, and dot color indicates mean accessibility. (c) Violin plot showing the consistency of co‐accessibility patterns for loci linked to the SOX10 promoter versus loci linked to the SOX10 3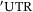. (d) Heatmap of peak‐module activity (z‐scored module accessibility) across cell states, highlighting modules with state‐biased accessibility programs. (e) UMAP projections of representative peak modules (modules indicated above each panel), showing spatially localized module activity across melanoma cell populations. (f) Kaplan–Meier survival analyses for four candidate genes (ELAPOR1, LOXL4, SMAP2, SSB) derived from the PAIR‐inferred regulatory program, stratifying patients into high‐ and low‐expression groups; shaded areas denote confidence intervals, and P values are from log‐rank tests.

In this dataset, PAIR preserves well‐separated cell manifolds (Figure 6a) and enables locus‐centered interrogation of regulatory neighborhoods. Focusing on SOX10, we summarized peaks that are co‐accessible with the SOX10 promoter versus the SOX10 3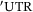 region (Figure 6b). These co‐accessible sets show structured, condition‐dependent accessibility patterns across melanoma states/time points, indicating that PAIR‐derived co‐accessibility captures coordinated regulation rather than isolated peak effects. Consistently, the “consistency” summary (Figure 6c) suggests that promoter‐anchored versus 3
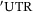
‐anchored co‐accessible neighborhoods can differ in how reproducibly they track cell states/conditions, reflecting distinct regulatory architectures around the same gene locus.

We next organized peaks into peak modules based on their co‐accessibility connectivity and summarized module activity across cell types (Figure 6d), revealing modules that are selectively active in specific melanoma states. Projecting module scores back onto the cell UMAP (Figure 6e) further highlights that individual modules map to discrete regions of the manifold, supporting an interpretable correspondence between “where cells sit” in embedding space and “which regulatory programs” are active. We performed GO enrichment on the top 100 genes whose promoters were most strongly co‐accessible with the SOX10 promoter peak (Figure S8). The enriched terms highlighted two major functional axes relevant to melanoma biology. First, multiple cell‐cycle–related processes, such as regulation of mitotic cytokinesis and protein localization to the cleavage furrow, suggested that SOX10‐linked regulatory programs capture proliferative states, consistent with the established role of SOX10 in melanoma growth and lineage maintenance [27]. Second, enrichment for cytoskeleton and trafficking components, including actin cytoskeleton, podosome/invadopodia‐associated compartments, endocytic vesicles, and phosphatidylinositol/phospholipid binding, pointed to coordinated regulation of migration/invasion‐associated machinery and membrane remodeling, processes broadly implicated in melanoma invasion and metastatic potential [28]. We also observed SCF/KIT‐related terms, consistent with the known relevance of KIT signaling in melanocyte biology and in specific melanoma subtypes [29].

Furthermore, we assessed the clinical relevance of the gene by performing Kaplan–Meier survival analyses in a melanoma patient cohort. We identified six genes: ELAPOR1, LOXL4, SMAP2, SSB, TARS3, and VAV3 (Figure 6f; Figure S8), whose expression levels significantly stratified patient survival, indicating that the regulatory programs captured from scATAC‐seq are reflected in clinically meaningful outcome differences. Functionally, these candidates are consistent with pathways known to shape melanoma progression. For example, LOXL4 belongs to the lysyl oxidase family that remodels and cross‐links the extracellular matrix, a process closely tied to tumor invasion and metastatic dissemination; related LOX/LOXL enzymes have been directly implicated in melanoma progression and microenvironmental remodeling [30]. In addition, SMAP2 is linked to membrane trafficking/endocytic regulation, and altered proteostasis and trafficking pathways are well recognized as key determinants of melanoma cell survival, metastatic competence, and therapy response [31].

### 2.6 PAIR Improves Cell‐Type Resolution and Recovers Transcription Factor Regulatory Programs

To evaluate whether imputation enhances downstream biological interpretation, we re‐performed clustering and regulatory analyses using the imputed accessibility matrix produced by PAIR and compared it with the raw data and representative baselines.

**FIGURE 7:**
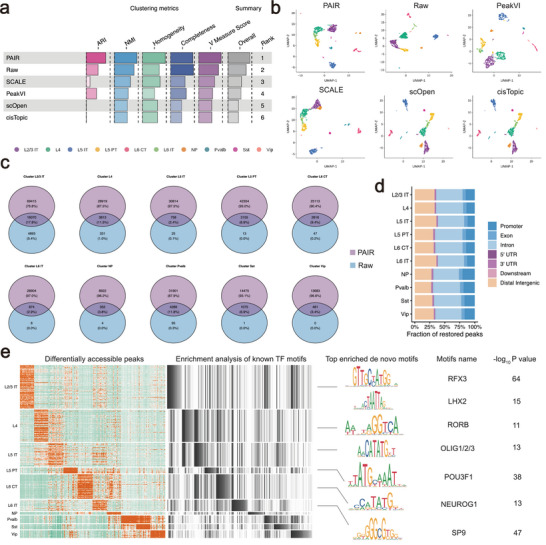
PAIR improves Cell‐Type Resolution and Recovers Transcription Factor Regulatory Programs. (a) Clustering performance after re‐clustering on imputed profiles. PAIR is compared with the raw matrix and baseline methods using multiple clustering metrics (ARI, NMI, homogeneity, completeness, and V‐measure), together with an overall summary and ranking. (b) UMAP visualization of cell embeddings derived from PAIR‐imputed data, the raw matrix, and representative baselines, colored by cell‐type annotations. (c) Differential accessibility analysis before and after imputation. For each cell type, Venn diagrams summarize the overlap of differentially accessible peaks (DAPs) identified from the PAIR‐imputed matrix versus the raw matrix. (d) Genomic annotation of restored DAPs. Stacked bar plots show the fraction of restored peaks assigned to genomic categories (such as promoter, exon, intron, UTRs, downstream, and distal intergenic regions) based on peak annotation. (e) Cell‐type–resolved regulatory interpretation. Left: heatmap of cell‐type‐specific DAP accessibility patterns. Right: enrichment analysis of known transcription factor motifs and de novo motifs from DAPs, highlighting top enriched motifs and their statistical significance.

We first re‐computed cell embeddings from the imputed matrix and re‐clustered the cells. Across multiple clustering metrics (ARI, NMI, homogeneity, completeness, and V‐measure), PAIR consistently achieved the best overall performance and ranked first among all compared approaches (Figure 7a). These improvements were also visually supported by the UMAP projections, where PAIR yielded clearer separation between major neuronal and interneuron populations with reduced mixing between closely related cell types (Figure 7b). UMAP is widely used to visualize high‐dimensional single‐cell embeddings while preserving local neighborhood structure. Next, we performed differential accessibility analysis with Signac [18] for each cell type using the raw versus imputed matrices. Compared with raw data, PAIR increased the number of detected differentially accessible peaks while maintaining substantial overlap with peaks identified from the raw matrix (Figure 7c). This pattern suggests that imputation not only preserves core cell‐type–specific regulatory signals but also boosts sensitivity for detecting weaker yet consistent accessibility differences [32, 33]. To characterize the genomic context of the restored peaks, we annotated differential peaks using ChIPseeker [34], which provides peak annotation and visualization utilities and reports genomic feature assignments for peaks based on transcript annotations. Then we summarized their distribution across genomic categories (promoter, exon, intron, UTRs, downstream, and distal intergenic regions) (Figure 7d). We observed that the restored peaks spanned both promoter‐proximal and distal regulatory regions, consistent with the idea that cell‐type identity is reflected by coordinated accessibility patterns across multiple regulatory elements.

To link restored distal accessibility to putative upstream regulators, we performed motif enrichment on cell‐type‐specific differentially accessible (DA) peaks restricted to distal regulatory regions. We tested curated TF motif libraries for over‐representation within each distal DA peak set and summarized the most significant known motifs alongside the top de novo motifs, treating enriched motifs as hypothesis‐generating candidates for TFs that may act through long‐range cis‐regulatory elements to shape cell‐type‐specific accessibility programs. Across neuronal populations, the enriched motifs recovered TFs with well‐established roles in forebrain and cortical patterning. For instance, RORB motifs were enriched in distal DA peaks of layer 4 excitatory neurons, consistent with RORβ’s prominent association with layer‐IV/barrel‐cortex identity and layer‐specific cortical programs [35]. Likewise, LHX2 motifs were enriched in excitatory‐lineage peak sets, aligning with LHX2's key function in specifying and maintaining cortical identity during development [36]. In addition, enrichment of RFX‐family motifs, such as RFX3, suggested contributions from regulators linked to brain developmental programs that depend on cilia‐associated gene regulation (notably in ventricular/ependymal contexts), which can intersect with broader neurodevelopmental regulation. Finally, motifs from the OLIG family (such as OLIG2) and POU3F1 were consistent with recovery of accessibility signatures related to neural lineage commitment and glial‐associated programs when present in the data [37, 38, 39].

Using GREAT [40], we assigned PAIR‐restored distal DA peaks (ChIPseeker‐defined distal regulatory regions) to putative target genes and observed strong, cell‐type‐consistent GO enrichments across BP/CC/MF using the region‐based binomial model (Tables S1–S12). Notably, excitatory‐lineage peak sets were enriched for neuronal compartment and synapse‐associated categories. For example, L5 PT distal peaks showed significant enrichment for synapse (GO:0045202), and L4 peaks were enriched for neurogenesis (GO:0022008) [41]. In contrast, inhibitory interneuron populations displayed comparatively stronger enrichments for structural and nuclear‐associated terms, such as actin cytoskeleton in Sst and Pvalb (GO:0015629) [42]. The restored peaks were distributed across both promoter‐proximal and distal regulatory regions. Functional enrichment of distal restored peaks was consistent with known neuronal biology, with excitatory populations biased toward synapse/neuron‐compartment terms and inhibitory populations showing relatively stronger enrichment for cytoskeletal and nuclear‐associated processes.

## 3 Discussion

Single‐cell ATAC‐seq provides a powerful window into cell‐state–specific regulatory landscapes, yet its analysis remains challenging due to intrinsically low coverage per cell and the resulting extreme sparsity and missingness in the cell‐by‐peak matrix. In this work, we introduced PAIR, a probabilistic cell–peak representation framework that directly models the bipartite structure of chromatin accessibility and learns uncertainty‐aware embeddings for both cells and peaks.

PAIR combines three ingredients to address core scATAC‐seq challenges. First, by constructing a cell–peak bipartite graph, PAIR avoids reliance on precomputed cell–cell similarities and instead preserves the native incidence structure of accessible regions across cells. Second, a variational latent layer explicitly models uncertainty in node representations, which is important under heterogeneous coverage and pervasive dropout. Third, PAIR adopts a dual‐decoder design: a qualitative decoder reconstructs open/closed accessibility structure, while a quantitative decoder models over‐dispersed accessibility counts using a Negative Binomial likelihood. Together, these components encourage the model to capture both discrete accessibility patterns and count‐level variability, improving robustness for downstream analyses such as clustering, batch‐aware integration, and regulatory program discovery. PAIR complements and differs from existing scATAC‐seq analysis paradigms. Deep generative models such as PeakVI and SCALE learn latent representations using variational formulations to account for technical factors and sparsity, while scDEC jointly learns embeddings and clusters via a deep generative clustering strategy. Within this landscape, PAIR's bipartite graph formulation and dual reconstruction objectives provide an alternative route to denoising and representation learning that is naturally aligned with peak‐level regulatory interpretation.

Beyond methodological improvements, PAIR is designed to be useful in clinically oriented scATAC‐seq studies, where patient samples often have limited material, heterogeneous coverage, and substantial missingness. By jointly reconstructing the open or closed incidence structure and the count‐level accessibility signal, PAIR outputs denoised accessibility profiles and uncertainty‐aware cell and peak representations that can support disease‐relevant stratification and biomarker discovery. In our melanoma case study, the learned peak level structure enables module‐based regulatory interpretation and identifies gene sets whose expression stratifies patient survival in an external cohort, illustrating how regulatory programs inferred from scATAC‐seq can be linked to clinically meaningful outcomes. More broadly, the imputed accessibility matrix can improve sensitivity for downstream analyses that are commonly used in translational pipelines, such as differential accessibility testing and motif enrichment for prioritizing candidate transcriptional regulators and pathways.

In practice, we envision PAIR as a preprocessing and feature learning layer that produces corrected accessibility estimates and interpretable peak features, which can be integrated with clinical covariates, treatment response, and matched bulk or single cell assays to nominate regulatory biomarkers and hypotheses for follow‐up. We note that translation to clinical decision making will require careful cohort‐level validation, robust control of batch and sample processing effects, and assessment of reproducibility across protocols and sequencing platforms, and we consider these important directions for future work.

Several limitations suggest opportunities for future improvement. (i) Although the Negative Binomial decoder captures over‐dispersion, scATAC‐seq data may exhibit additional structure, such as zero inflation driven by both biology and technical dropouts, motivating exploration of richer likelihoods or hierarchical dispersion models. (ii) The size of cell–peak graphs can be massive; while sparse operations help, further scalability gains may be achieved through subgraph training, adaptive neighbor sampling, or multi‐resolution peak representations. Looking forward, several biologically motivated extensions could further strengthen PAIR's ability to recover transcription factor activity and regulatory programs. One direction is to incorporate sequence‐level priors as peak attributes, enabling the model to connect accessibility patterns to TF binding logic more directly. Another is to extend the graph beyond incidence edges by adding peak–peak co‐accessibility links or gene‐centric regulatory constraints, which may help disentangle shared regulatory modules across cell types. Finally, although PAIR is developed for scATAC‐seq, the framework can be extended to multi‐omic integration in two complementary ways. One direction is to generalize the current cell–peak bipartite graph to a heterogeneous graph by introducing additional feature types and adding biologically informed cross‐feature links, such as peak–gene regulatory connections or co‐accessibility–guided constraints. This design enables information flow across modalities through message passing while retaining modality‐specific decoders for reconstruction. A second direction is to avoid explicit cross‐feature links by constructing a separate cell‐feature bipartite graph for each modality and aligning the resulting cell embeddings in a shared latent space; each modality can then be modeled with its own encoder/decoder while integration is achieved through latent alignment. Key challenges for both routes include the uncertainty and context dependence of cross‐modal links, balancing learning signals across modalities with different sparsity and noise characteristics, handling partially paired datasets with missing modalities, and scaling training as additional graphs or node/edge types increase computational cost. Future work will further improve its interpretability and scalability for mapping TF‐driven regulatory programs across diverse biological systems.

## 4 Methods

### 4.1 scATAC‐seq Data Processing

We started from the raw scATAC‐seq count matrix $X_{raw}\in R^{m\times n}$, where n denotes the number of cells and m denotes the number of peaks, and $x_{ij}$ is the accessibility count of peak j in cell i and non‐negative integer. We performed quality control using Scanpy's built‐in filtering utilities sc.pp.filter _cells and sc.pp.

filter_genes, where peaks were treated as “genes” in an AnnData object). Specifically, we removed low‐quality cells with insufficient total fragments and discard low‐information peaks that were rarely observed across the dataset. Concretely, cells were filtered based on the total number of nonzero peak counts (or total fragments), and peaks were filtered based on their prevalence across cells (number of cells with nonzero counts). After filtering, the resulting matrix was denoted again by X for simplicity.

To account for cell‐specific sequencing depth, we compute a library size factor for each cell. We define

(1) $$l_{i}=\frac{\sum_{j=1}^{M}x_{ij}}{{\mathrm{median}}_{i^{\prime }}\left\{\sum_{j=1}^{M}x_{i^{\prime }j}\right\}}$$

each cell's total fragment count was normalized by the median total count across cells. The factor $l_{i}$ is later used to calibrate the magnitude of reconstructed counts. For peak, instead of using a fixed heuristic peak statistic, we introduced a learnable peak factor $s_{j}$ for each peak j. We parameterized $s_{j}$ with a positivity‐preserving transform and optimized it jointly with the model parameters. Conceptually, this factor captured peak‐specific baseline accessibility effects and complements the cell library size factor during reconstruction.

#### 4.1.1 Bipartite Graph Construction

In contrast to prevailing approached that construct cell–cell graphs from precomputed similarity measures, we build a bipartite cell–peak graph. Specifically, the graph contains two types of nodes, cells and peaks, and we add an edge between a cell and a peak whenever that peak was accessible in the binarized open/closed chromatin matrix. Specifically, starting from the filtered cell‐by‐peak count matrix X, we construct a binary incidence matrix R by $r_{ij}=1\left(x_{ij}>0\right)$; equivalently, an edge $\left(u_{i},v_{j}\right)\in E$ exists whenever $x_{ij}>0$. This design choice was motivated by the observation that the resulting cell–peak graph directly encoded the original chromatin accessibility patterns in the binary matrix, without introducing an intermediate dependency on cell–cell similarity calculations.

In formal terms, we denoted the bipartite graph as G=(U,V,E). In this graph, U represents the set of m cell nodes $\left\{u_{1},u_{2},\text{…},u_{m}\right\}$, while V represents the set of n peak nodes $\left\{v_{1},v_{2},\text{…},v_{n}\right\}$. Each feature node corresponds to a distinct feature dimension within the input data. The set of edges, denoted as E and defined as E⊆U×V, encapsulates the relationships between cells and features. This graph structure could be succinctly represented as an adjacency matrix, further elucidating the connectivity and interactions within the dataset: $A=\left(\begin{array}{cc} O & R\\ R^{T} & O \end{array}\right)\in R^{\left(m+n)\times (m+n\right)}$, where O is a matrix filled with constant 0 and R is the binary chromatin accessibility matrix, where $r_{ij}=1$ indicates peak j is accessible in cell i and 0 otherwise. It's crucial to note that A represents a bipartite graph, where nodes within the same set (either peak nodes or cell nodes) were not directly connected. This inherent bipartite characteristic ensured the distinct separation between cell‐related and feature‐related nodes, preserving the integrity of the graph's structure.

### 4.2 Regulatory Graph Convolution

During gene regulation, chromatin accessibility exhibits structured dependencies at multiple levels. In particular, three types of relationships are typically involved: (i) cell–chromatin associations that link each cell to the set of peaks that were open within that cell, (ii) intra‐cell chromatin dependencies reflecting coordination among different accessible regions within the same cell (such as co‐accessible peaks), and (iii) inter‐cell chromatin state similarities capturing shared open/closed patterns across cells. A bipartite regulatory graph offered a unified representation to model these dependencies: the cell–peak edges explicitly encode type‐(i) relations, while higher‐order neighborhoods induced by message passing naturally recover type‐(ii) and type‐(iii) relations through multi‐hop connectivity (such as cell → peak → cell for inter‐cell similarity, and peak → cell → peak for intra‐cell co‐accessibility).

To simultaneously capture these three relationship types, we employed a regulatory graph convolutional network based on LightGCN [43]. LightGCN was particularly suitable for bipartite graphs because it focused on neighborhood aggregation with normalized propagation, while discarding nonlinear feature transformations and explicit self‐loop injections. This design reduced over‐parameterization and often yields more stable training on large, sparse graphs, which was common in single‐cell chromatin accessibility data.

Given the bipartite cell–peak graph, the propagation rule at the (k+1)‐th layer is defined as:

(2) $$e_{c}^{\left(k+1\right)}=\sum_{p\in N_{c}}\frac{1}{\sqrt{\left|N_{c}\right|}\sqrt{\left|N_{p}\right|}}e_{p}^{\left(k\right)},\;e_{p}^{\left(k+1\right)}=\sum_{c\in N_{p}}\frac{1}{\sqrt{\left|N_{p}\right|}\sqrt{\left|N_{c}\right|}}e_{c}^{\left(k\right)}$$

where $e_{c}$ denotes the ID embedding of cell c and $e_{p}$ denotes the ID embedding of peak p. Here, $N_{c}$ is the set of peak neighbors connected to cell c, and $N_{p}$ is the set of cell neighbors connected to peak p. The symmetric normalization term $\frac{1}{\sqrt{|N_{c}\left|\right|N_{p}|}}$ prevents high‐degree nodes from dominating the aggregation and encourages balanced information flow between cells and peaks.

Embeddings learned at different propagation depths encode complementary semantics. The first propagation layer primarily enforced local smoothness, making cells and peaks directly connected by observed accessibility edges more similar. The second layer aggregated information from two‐hop neighbors, enabling the model to capture cell–cell similarity via shared peaks (cell → peak → cell) and peak–peak co‐accessibility via shared cells (peak → cell → peak). As the number of layers increases, the embeddings progressively incorporate higher‐order proximity, effectively capturing more global regulatory structure in the bipartite graph. Therefore, we form the final representation by combining embeddings from multiple layers, which empirically provides a more expressive and robust representation than using a single layer. This layer‐wise aggregation could also be interpreted as implicitly incorporating self‐information through residual‐like mixing across propagation depths, improving the ability of the model to represent complex dependencies in sparse single‐cell graphs.

Let the 0‐th layer embedding matrix be $E^{\left(0\right)}\in R^{(m+n)\times d}$, where d is the embedding dimension, and m and n are the numbers of cells and peaks, respectively. Then the regulatory graph convolution in matrix form can be written as:

(3) $$E^{\left(k+1\right)}=\left(D^{-\frac{1}{2}}AD^{-\frac{1}{2}}\right)E^{\left(k\right)}$$

where $A\in R^{\left(m+n)\times (m+n\right)}$ is the adjacency matrix of the bipartite graph, and D is the corresponding diagonal degree matrix with $D_{ii}=\sum_{j}A_{ij}$. This normalized propagation operator defines the message passing backbone for the regulatory graph encoder.

### 4.3 Variational Cell–Peak Latent Bottleneck

Single‐cell chromatin accessibility data were extremely sparse and noisy, and a purely deterministic encoder may overfit spurious edges while failing to reflect uncertainty in the inferred representations. Inspired by the variational graph reconstruction idea in VGAE [44], which estimated a node‐wise Gaussian distribution and generated representations via sampling, we introduce a variational bottleneck between the bipartite GNN encoder, inferring stochastic latent embeddings for both cells and peaks from the scATAC‐seq cell–peak graph.

#### 4.3.1 Node‐Wise Gaussian Posterior

After K layers of regulatory graph convolution, we obtain layer‐wise embeddings ${\left\{E^{\left(k\right)}\right\}}_{k=0}^{K}$ for all nodes (cells and peaks). We aggregated multi‐layer embeddings into a deterministic representation

(4) $$\bar{E}=\mathrm{READOUT}\left(E^{\left(0\right)},E^{\left(1\right)},\text{…},E^{\left(K\right)}\right)$$

and use it to parameterize a factorized Gaussian approximate posterior for each node i∈U∪V:

(5) $$q_{\phi }\left(Z|A,E^{\left(0\right)}\right)=N\;\left(\mu_{i},\mathrm{diag}\left(\sigma_{i}^{2}\right)\right)$$

Specifically, we set the posterior mean to the aggregated embedding, i.e., $\mu_{i}={\bar{e}}_{i}$. For the uncertainty term, we adopt a lightweight parametric estimator, where the variance is learned from the mean via a small MLP. In practice, we parameterize the diagonal standard deviation as

(6) $$\sigma_{i}=\mathrm{softplus}\;\left(\mathrm{MLP}\left(\mu_{i}\right)\right)$$

which ensures positivity and yields node‐adaptive uncertainty. This design allowed different cells and peaks to had different confidence levels, which was particularly important under long‐tail coverage and heterogeneous sparsity.

#### 4.3.2 Reparameterization and Sampling

To enable end‐to‐end optimization, we employ the reparameterization trick:

(7) $$z_{i}=\mu_{i}+\sigma_{i}⊙\varepsilon ,\;\varepsilon ∼N\left(0,I\right)$$

where ⊙ denotes element‐wise multiplication.

### 4.4 Qualitative Decoder via Graph Generation

Following the graph generation principle, we modelled the qualitative chromatin accessibility pattern by reconstructing the binary cell–peak incidence graph. Given the binarized open/closed matrix $R\in {\left\{0,1\right\}}^{m\times n}$ (equivalently, the bipartite adjacency between cells and peaks), our goal is to generate R from the latent embeddings inferred by the variational hidden layer.

#### 4.4.1 Generative Likelihood for the Bipartite Graph

Let $z_{i}^{\left(c\right)}\in R^{d}$ be the latent embedding of cell i and $z_{j}^{\left(p\right)}\in R^{d}$ be the latent embedding of peak j. We assumed conditional independence over edges and factorized the likelihood over all cell–peak pairs:

(8) $$p_{\theta }\left(R|Z^{\left(c\right)},Z^{\left(p\right)}\right)=\prod_{i=1}^{N}\prod_{j=1}^{M}p_{\theta }\left(r_{ij}|z_{i}^{\left(c\right)},z_{j}^{\left(p\right)}\right)$$

For the qualitative decoder, we adopted a Bernoulli edge model:

(9) $$p_{\theta }\left(r_{ij}=1|z_{i}^{\left(c\right)},z_{j}^{\left(p\right)}\right)=\sigma \;\left(h_{ij}\right),\;h_{ij}={z_{i}^{\left(c\right)}}^{⊤}z_{j}^{\left(p\right)}$$

where σ(·) is the sigmoid function. This inner‐product parameterization was a simple yet effective choice for graph generation. Intuitively, $\sigma (h_{ij})$ represents the probability that peak j is accessible (open) in cell i.

#### 4.4.2 Qualitative Reconstruction Objective

The qualitative decoder contributed the edge‐reconstruction term in the variational objective. Specifically, it appeared as the expected log‐likelihood under the approximate posterior, as in the ELBO formulation of VGAE. Because R is extremely sparse, we optimized the reconstruction term with negative sampling and a pairwise learning strategy. For each cell i, we sample a positive peak $j^{+}$ from its observed open set $N_{i}$ and a negative peak $j^{-}$ from unobserved entries, and minimize

(10) $$L_{\mathrm{qual}}=\sum_{i=1}^{N}\;E_{\left(j^{+},j^{-}\right)∼D_{i}}\left[-\log\sigma \;\left(h_{ij^{+}}-h_{ij^{-}}\right)\right]$$

where $D_{i}$ denotes the sampling distribution over positive–negative peak pairs for cell i. This objective encourages observed open peaks to receive higher scores than sampled closed peaks, yielding a robust qualitative reconstruction signal under severe sparsity.

### 4.5 Quantitative Decoder with a Negative Binomial Likelihood

To model the quantitative chromatin accessibility signal, we decoded the observed scATAC‐seq count matrix $X=\left[x_{ij}\right]\in R^{m\times n}$ using a Negative Binomial (NB) likelihood. Given the latent representations of cells and peaks sampled from the variational hidden layer, the decoder predicts distribution parameters for each cell–peak pair and maximized the corresponding likelihood.

#### 4.5.1 Latent Inputs

We define a lightweight interaction feature by the Hadamard product

(11) $$h_{ij}^{\prime }=z_{i}^{\left(c\right)}⊙z_{j}^{\left(p\right)}$$

#### 4.5.2 Mean Factorization with Depth and Peak Effects

To account for cell‐specific sequencing depth and peak‐specific baseline accessibility, we introduce a pre‐computed cell library size factor $l_{i}>0$ and a learned peak factor $s_{j}>0$. The decoder first produces a positive interaction intensity ${\tilde{\mu }}_{ij}>0$ and then rescales it:

(12) $$\begin{array}{cc} {\tilde{\mu }}_{ij} & =\mathrm{softplus}\;\left(w_{\mu }^{⊤}h_{ij}^{\prime }+b_{\mu }\right) \end{array}$$

(13) $$\begin{array}{cc} \mu_{ij}^{\prime } & =l_{i}\cdot s_{j}\cdot {\tilde{\mu }}_{ij} \end{array}$$

here $\mu_{ij}^{\prime }$ serves as the NB mean parameter.

#### 4.5.3 Dispersion Modeling

We parameterized a peak‐specific inverse‐dispersion as a positive quantity. For parameter efficiency, we use a peak‐specific inverse‐dispersion parameter $\theta_{j}>0$:

(14) $$\theta_{j}=\mathrm{softplus}\left(u_{j}^{\prime }\right)$$

Under the $\left(\mu^{\prime },\theta \right)$ parameterization, $E\left[x_{ij}\right]=\mu_{ij}^{\prime }$ and $\mathrm{Var}\left(x_{ij}\right)=\mu_{ij}^{\prime }+\mu_{ij}^{\prime 2}/\theta_{j}$. We also define a peak‐specific dispersion parameter $\phi_{j}$ by

(15) $$\phi_{j}=\frac{1}{\theta_{j}}$$

which yields the equivalent variance form $\mathrm{Var}\left(x_{ij}\right)=\mu_{ij}^{\prime }+\phi_{j}\mu_{ij}^{\prime 2}$. In this terminology, larger $\phi_{j}$ corresponds to stronger over‐dispersion.

#### 4.5.4 NB Likelihood

Under the $\left(\mu^{\prime },\theta \right)$ parameterization, we assume

(16) $$x_{ij}∼\mathrm{NB}\left(\mu_{ij}^{\prime },\theta_{j}\right)$$

with probability mass function

(17) $$p\left(x_{ij}|\mu_{ij}^{\prime },\theta_{j}\right)=\frac{\Gamma (x_{ij}+\theta_{j})}{\Gamma \left(\theta_{j}\right)\;\Gamma \left(x_{ij}+1\right)}{\left(\frac{\mu_{ij}^{\prime }}{\mu_{ij}^{\prime }+\theta_{j}}\right)}^{x_{ij}}{\left(\frac{\theta_{j}}{\mu_{ij}^{\prime }+\theta_{j}}\right)}^{\theta_{j}}$$

#### 4.5.5 Quantitative Reconstruction Objective

The quantitative decoder is trained by minimizing the NB negative log‐likelihood:

(18) $$\begin{array}{ccc} L_{quan}\left(x_{ij};\mu_{ij}^{\prime },\theta_{j}\right) & = & -\log p(x_{ij}|\mu_{ij}^{\prime },\theta_{j})\\ & = & -\log\Gamma \left(x_{ij}+\theta_{j}\right)+\log\Gamma \left(\theta_{j}\right)+\log\Gamma \left(x_{ij}+1\right)\\ & & +\theta_{j}\log\left(\mu_{ij}^{\prime }+\theta_{j}\right)-\theta_{j}\log\theta_{j}\\ & & +x_{ij}\log\left(\mu_{ij}^{\prime }+\theta_{j}\right)-x_{ij}\log\mu_{ij}^{\prime } \end{array}$$

### 4.6 Overall Training Objective

The model was trained by jointly optimizing the qualitative decoder, the quantitative decoder, and the variational hidden layer. Let $L_{\mathrm{qual}}$ denote the qualitative loss defined in the previous subsection, and let $L_{\mathrm{quan}}$ denote the quantitative NB reconstruction loss. In addition to the KL regularization induced by the variational hidden layer, we apply an $\ell_{2}$ penalty on the trainable 0‐th layer ID embeddings $E^{\left(0\right)}$ to prevent overfitting and stabilize optimization.

#### 4.6.1 KL Regularization

With a standard Gaussian prior $p\left(z_{i}\right)=N\left(0,I\right)$ for each node i∈U∪V, the variational regularizer is

(19) $$L_{\mathrm{KL}}=\sum_{i\in U\cup V}\mathrm{KL}\;\left(q_{\phi }\left(z_{i}|A,E^{0}\right)\;∥\;N\left(0,I\right)\right)$$

#### 4.6.2 $\ell_{2}$ Regularization on $E^{\left(0\right)}$

We regularize the 0‐th layer embedding matrix using the squared Frobenius norm:

(20) $$L_{\mathrm{reg}}={\left\|E^{\left(0\right)}\right\|}_{F}^{2}$$

#### 4.6.3 Total Loss

The final training objective is a weighted sum of all components:

(21) $$L=L_{\mathrm{qual}}+\alpha L_{\mathrm{quan}}+L_{\mathrm{KL}}+\beta \;L_{\mathrm{reg}}$$

where α balance the qualitative and quantitative decoders, and β controls the $\ell_{2}$ penalty on $E^{\left(0\right)}$.

### 4.7 Hyperparameter Settings

We adopted a lightweight graph encoder based on layer‐wise neighborhood aggregation, where the number of propagation layers is set to K=3. All node representations were projected into a d=64 D latent space. Model parameters were optimized using the Adam optimizer with a learning rate of $5\times 10^{-3}$. For the overall objective, we used two scalar coefficients to balance different loss components: α controls the relative weight of the reconstruction terms (qualitative vs. quantitative), and β controls the strength of the regularization. Unless otherwise specified, we set α=1 and $\beta =10^{-3}$.

### 4.8 Comparison with Baseline Methods and Evaluation

The performance of PAIR was compared with several state‐of‐the‐art scATAC‐seq data methods. The parameters used for the baseline methods were directly adopted from their original research papers.

- PeakVI: A deep generative model for scATAC‐seq that used variational inference to learn a low‐dimensional cell representation while estimating peak accessibility probabilities, with explicit cell‐wise and region‐wise scaling factors to account for technical effects (https://docs.scvi‐tools.org/en/1.3.3/user_guide/models/peakvi.html).
- SCALE: A variational autoencoder framework tailored to sparse scATAC‐seq data that extracted latent features and combines them with a Gaussian mixture model to support unsupervized cell clustering in the latent space (https://github.com/jsxlei/SCALE).
- scOpen: An imputation method based on regularized non‐negative matrix factorization that recovers open‐chromatin signals from sparse scATAC‐seq counts and outputs an imputed accessibility matrix for downstream analysis (https://github.com/CostaLab/scopen).
- cisTopic: A topic‐modeling approach that identified co‐accessible regulatory “topics” (sets of peaks) and stable cell states from sparse single‐cell epigenomic data, enabling clustering and regulatory interpretation (https://github.com/aertslab/cisTopic).
- EpiScanpy: A Scanpy‐compatible toolkit for single‐cell epigenomics that provided an end‐to‐end workflow, including preprocessing, dimensionality reduction, clustering, and downstream analyses for scATAC‐seq and related assays (https://episcanpy.readthedocs.io/en/latest/).
- scDEC: A deep generative clustering method for scATAC‐seq built on GAN‐based components that simultaneously learned a latent embedding and infers cell cluster assignments in an unsupervized manner (https://github.com/kimmo1019/scDEC).
- SnapATAC2: A fast, scalable end‐to‐end analysis toolkit for single‐cell epigenomics that covered preprocessing, dimensionality reduction, clustering, integration, peak calling, differential analysis, motif analysis, and regulatory network analysis (https://scverse.org/SnapATAC2/).

#### 4.8.1 Peak Module Analysis and Visualization

We defined peak modules directly from the learned peak representations produced by PAIR and summarized their activities across cell states as a heatmap. After training PAIR, we extracted the peak embeddings, which provides a low‐dimensional representation for each peak. These embeddings serve as feature vectors for defining peak–peak similarity in a denoised representation space rather than using the raw sparse count matrix. To derive peak modules, we constructed a k‐nearest‐neighbor graph among peaks in the embedding space using cosine distance. Specifically, we built a peak‐only AnnData object whose rows correspond to peaks and whose matrix X stores the extracted peak embeddings. We then computed the kNN graph. Given this peak–peak graph, we applied Leiden community detection (Scanpy sc.tl.leiden) to obtain discrete peak modules. The resulting module label for each peak was stored in adata.var. To quantify how active each peak module was in each cell, we computed a per‐cell module score based on binary accessibility. We binarized the QC‐filtered cell‐by‐peak count matrix by setting an entry to 1 if the corresponding count was nonzero and 0 otherwise. For each module, we computed the module activity in a cell as the mean of these binary accessibility values over all peaks assigned to the module, so that the score could be interpreted as the fraction of peaks in the module that were accessible in the cell. Implementation‐wise, we constructed a peak‐to‐module one‐hot assignment matrix and computed the cell‐by‐module score matrix by matrix multiplication followed by division by the number of peaks in each module. To generate the heatmap in Figure 5d, we aggregated module activity scores by cell type. For each cell type, we computed the mean module activity across all cells of that type, yielding a cell‐type‐by‐module matrix. To highlight state‐specific relative activity patterns and improve comparability across modules, we z‐score normalized each module across cell types using the module‐wise mean and standard deviation, with a small constant added to the denominator for numerical stability. Finally, we plotted the resulting z‐scored matrix as a heatmap, where rows correspond to cell types and columns correspond to peak modules, and values indicate the relative module accessibility enriched or depleted in each cell state.

#### 4.8.2 Gene Selection for Survival Analysis

We first constructed a SOX10‐centered regulatory neighborhood from the PAIR‐derived peak representation by computing peak–peak co‐accessibility and identifying peaks connected to the SOX10 promoter peak. We then annotated promoter‐associated peaks to genes and ranked genes by the co‐accessibility strength between their promoter peak(s) and the SOX10 promoter peak. The top 100 genes were used as a predefined candidate set for downstream functional enrichment (Figure S8). We subsequently evaluated clinical relevance in an external melanoma patient cohort by performing Kaplan–Meier analyses for each candidate gene, stratifying patients into high‐ and low‐expression groups and computing log‐rank test statistics (Figure 5f; Figure S8).

### 4.9 Simulated scATAC‐seq Dataset Generation

To systematically assess robustness to dropout rate, sequencing depth and noise, we generated simulated scATAC‐seq datasets by adapting the simulation procedure described in Ref.[45]. We constructed synthetic scATAC‐seq matrices with an explicit cluster‐specific cell–peak structure, where peaks were enriched in accessibility within their corresponding cell groups and largely inactive across mismatched groups, thereby yielding a clear block‐pattern in the underlying signal. To mimic the extreme sparsity typically observed in scATAC‐seq data, we then introduced dropout by randomly masking observed (nonzero) cell–peak entries to zero, generating datasets with dropout rates ranging from 0.5 to 0.8. This produced a controlled series of increasingly sparse datasets that emulate technical missingness/dropout effects widely discussed in single‐cell sequencing and scATAC‐seq analysis. We used the bone marrow bulk ATAC‐seq profiles [26] with clear cell‐type annotations as the reference signal source, and simulated single‐cell cell‐by‐peak count matrices under controlled read coverage and noise settings.

#### 4.9.1 Bulk‐Derived Peak Prevalence

Let k be the number of peaks and let t index a cell type (sorted population). From the bulk ATAC‐seq peak‐by‐cell‐type count matrix, we computed the prevalence rate of peak i in cell type t as

(22) $$r_{i}^{t}=\frac{\text{reads in peak}\;i\;\text{for cell type}\;t}{\sum_{i^{\prime }=1}^{k}\text{reads in peak}\;i^{\prime }\;\text{for cell type}\;t}$$

which represents the relative accessibility of peak i in the bulk profile of cell type t.

#### 4.9.2 Noise and Coverage Controls

Following the referenced simulation, we introduced (i) a noise parameter q∈[0,1], defined as the fraction of reads redistributed uniformly across peaks (mimicking non‐specific Tn5 cutting), and (ii) a coverage parameter n, representing the number of simulated fragments per cell. A noise level of q=0 preserved perfect cell‐type specificity, while larger q progressively weakens cell‐type‐specific peak patterns by injecting random reads.

#### 4.9.3 Single‐Cell Count Generation

For each simulated cell j assigned to cell type t, we generated a count $c_{i,j}\in \left\{0,1,2\right\}$ for each peak i (reflecting the diploid setting) by sampling

(23) $$c_{i,j}∼\mathrm{Binomial}\;\left(2,\;p_{i}^{t}\right)$$

where the success probability $p_{i}^{t}$ is defined as a mixture of a bulk‐informed component and a uniform‐noise component:

(24) $$p_{i}^{t}=\left(r_{i}^{t}\right)\left(\frac{1}{2}n\right)\left(1-q\right)+\left(\frac{1}{k}\right)\left(\frac{1}{2}n\right)q$$

Intuitively, when q→0, the first term dominates and $p_{i}^{t}$ is governed by the bulk peak prevalence $r_{i}^{t}$; when q→1, the second term dominates and fragments are distributed approximately uniformly across peaks, removing cell‐type‐specific structure.

##### 4.9.3.1 Simulation Grid Used in This Work

We generated a grid of bone marrow simulated datasets by varying sequencing depth and noise. Specifically, we set the per‐cell coverage to

(25) sequencingdepth∈{1000,2000,3000,4000,5000}

and considered three noise settings

(26) noiselevel∈{0.2,0.3,0.4}

### 4.10 Evaluation Metrics

For imputation on simulated and down‐sampling experiments, we adopted the Pearson correlation coefficient (PCC) to quantify agreement between reconstructed and reference signals. PCC was reported in both a cell‐wise manner (correlation across peaks within each cell) and a peak‐wise manner (correlation across cells for each peak), and then summarized across cells/peaks. To assess recovery of the binary open/closed accessibility pattern, we additionally computed AUROC and AUPRC based on prediction scores against binarized ground truth. For clustering analysis, we compared predicted clusters with reference cell‐type annotations using Adjusted Rand Index (ARI) and Normalized Mutual Information (NMI). We further reported V‐measure together with its two components, homogeneity and completeness, to provide a more interpretable decomposition of clustering performance. To evaluate batch correction and data integration, we used three widely adopted metrics: (i) integration LISI (iLISI), which measures local mixing of batch labels in the neighborhood of each cell, (ii) kBET, a kNN‐based statistical test that quantified deviations from expected batch mixing, and (iii) graph connectivity, which assesses whether cells of the same biological label remain connected in the integrated kNN graph by computing the fraction of cells in the largest connected component per label and averaging across labels. In addition, we computed the silhouette score to measure within‐cluster compactness and between‐cluster separation in the learned embedding space.

#### 4.10.1 Pearson Correlation Coefficient

The PCC quantifies the linear correlation between the imputed expression values and the true values of genes and cells, and it is defined as:

(27) $$\text{PCC}=\frac{\sum_{i=1}^{n}\left(x_{i}-\bar{x}\right)\left(y_{i}-\bar{y}\right)}{\sqrt{\sum_{i=1}^{n}{\left(x_{i}-\bar{x}\right)}^{2}\sum_{i=1}^{n}{\left(y_{i}-\bar{y}\right)}^{2}}}$$

where $x_{i}$ and $y_{i}$ are the imputed and true expression values of the same gene or cell, and $\bar{x}$ and $\bar{y}$ are their respective means. The result always had a value between ‐1 and 1.

#### 4.10.2 ARI

The Rand Index measured the agreement between two clusterings by evaluating the correct overlaps and disagreements between them [9]. Adjusted Rand Index (ARI) further refined the Rand Index by accounting for agreements expected by random chance. We performed Leiden [46] clustering for this metric to obtain the best match between clusters and labels. An ARI of 0 indicates random labeling, while an ARI of 1 denotes a perfect match. For this analysis, we utilized the scikit‐learn (v.0.22.1) [47] implementation of the ARI metric. It is defined as:

(28) ARI=∑i,jnij2−∑iai2∑jbj2/N212∑iai2+∑jbj2−∑iai2∑jbj2/N2

where $n_{ij}$ is the number of cells assigned to both cluster i and cluster j, $a_{i}$ is the number of cells assigned to cluster i, $b_{j}$ is the number of cells assigned to cluster j, and N is the total number of cells.

#### 4.10.3 NMI

Normalized Mutual Information (NMI) evaluated the overlap between two clusterings [10]. The overlap was normalized by the mean entropy of the cell type and cluster labels, with NMI scores ranging from 0 (indicating uncorrelated clustering) to 1 (representing a perfect match). We also used the scikit‐learn (v.0.22.1) implementation of NMI. It is defined as:

(29) $$\text{NMI}=\frac{2\sum_{k=1}^{K}\sum_{j=1}^{J}n_{kj}\log\left(\frac{N\cdot n_{kj}}{n_{k}\cdot n_{j}}\right)}{\sum_{k=1}^{K}n_{k}\log\left(\frac{N\cdot n_{k}}{n_{k}}\right)+\sum_{j=1}^{J}n_{j}\log\left(\frac{N\cdot n_{j}}{n_{j}}\right)}$$

where $n_{kj}$ is the number of cells assigned to both cluster k and cluster j, $n_{k}$ is the number of cells assigned to cluster k, $n_{j}$ is the number of cells assigned to cluster j, and N is is the total number of cells.

#### 4.10.4 ASW

The silhouette width measured the balance between within‐cluster distances and its between‐cluster distances to the nearest neighboring cluster [48]. The Average Silhouette Width (ASW), calculated as the mean silhouette width across a set of cells, ranges from ‐1 to 1. ASW was a widely used metric for evaluating cluster separation, with a value of 1 indicating tightly packed and well‐separated clusters. In contrast, an ASW value of 0 suggests overlapping clusters, where within‐ and between‐cluster variability were comparable, while a value of ‐1 indicates substantial misclassification, where within‐cluster variability exceeds between‐cluster variability. To assess the outputs of each method, we employed two approaches: (1) the classical definition of Average Silhouette Width (ASW) to evaluate the clustering of cell‐type labels (cell‐type ASW) and (2) a modified ASW to quantify batch mixing. Both metrics were calculated using the embeddings provided by the integration methods or, in cases of feature‐based outputs, the PCA of expression matrices. For the bio‐conservation score (1), the ASW was computed based on cell identity labels and scaled to a range between 0 and 1 using the following equation:

(30) $$\mathrm{cell}\;\mathrm{type}\;\mathrm{ASW}=({\mathrm{ASW}}_{C}+1)/2$$

where C denotes the set of all cell identity labels.

The batch mixing score (b_ASW) quantifies the degree of batch mixing within cell clusters. It is calculated by computing the absolute silhouette widths based on batch labels for each cell and scaling these values to a range between 0 and 1. The score is defined as:

(31) $$b\_\mathrm{ASW}=\frac{1}{\left|M\right|}\sum_{j\in M}\;\text{batch}\;{\mathrm{ASW}}_{j}$$

where M is the set of unique cell labels, $\;\text{batch}\;{\mathrm{ASW}}_{j}$ is to assess batch mixing within each cell label independently. It is defined as:

(32) $$\;\text{batch}\;{\mathrm{ASW}}_{j}=\frac{1}{\left|C_{j}\right|}\sum_{i\in C_{j}}1-s_{\text{batch}\;}\left(i\right)$$

where $C_{j}$ is the set of cells with the cell label j and $\left|C_{j}\right|$ denotes the number of cells in that set, and

$s_{\text{batch}\;}\left(i\right)$ is the absolute silhouette width based on the batch labels assigned to each cell, denoted as:

(33) $$s_{\text{batch}\;}\left(i\right)=\left|s\left(i\right)\right|$$

#### 4.10.5 LISI

The Local Inverse Simpson's Index (LISI) [49] was a metric used to quantify the degree of local diversity or separation between different groups. It was a variant of the Inverse Simpson's Index, computed from neighborhood lists per node from integrated k‐nearest‐neighbor (kNN) graphs. Specifically, the Inverse Simpson's Index quantifies the number of cells that could be drawn from a neighbor list before encountering a cell from the same batch. As a result, LISI scores range from 1 to B (where B is the total number of batches), indicating perfect separation and perfect mixing, respectively. We adopted the definitions of cLISI and iLISI from [50] to quantify cell‐type separation and batch integration, respectively. First, we computed the median LISI score for each method across all neighborhoods using the following formulas: $cLISI=median\;f\left(x\right),x\in X;iLISI=median\;g\left(x\right),x\in X$. Second, we rescaled the cLISI and iLISI scores: $f\left(x\right)=\frac{B-x}{B-1};g\left(x\right)=\frac{x-1}{B-1}$, where a 0 value corresponds to low cell‐type separation and batch integration.

#### 4.10.6 kBET

kBET [51] (k‐nearest neighbor batch effect test) was a statistical method specifically designed to detect and quantify batch effects in single‐cell RNA sequencing data. This algorithm operated by first identifying the kNN for each cell based on their gene expression profiles. Subsequently, it compared the distribution of batch labels among these neighbors to the overall distribution of batch labels within the dataset. A statistical test, typically a Pearson's $\chi^{2}$ test, was then employed to assess whether the observed distribution significantly deviated from the expected distribution under the null hypothesis of no batch effect. Here, we used the method provided in Ref. [50] to calculate this metric.

#### 4.10.7 Graph Connectivity

The graph connectivity metric evaluated the extent to which the kNN graph representation, G, of the integrated data, directly links cells with identical cell identity labels. For each cell identity label c, a subset kNN graph, $G(N_{c};E_{c})$, was constructed to encompass only cells belonging to that label. Using these subset kNN graphs, we computed the graph connectivity (GC) score as follows:

(34) $$\mathrm{GC}=\frac{1}{\left|C\right|}\sum_{c\in C}\frac{\left|\mathrm{LCC}\left(G\left(N_{c};E_{c}\right)\right)\right|}{\left|N_{c}\right|}$$

where C represents the set of cell identity labels, $\left|\mathrm{LCC}\left(G\left(N_{c};E_{c}\right)\right)\right|$ is the number of nodes in the largest connected component of the graph $G(N_{c};E_{c})$, and $\left|N_{c}\right|$ is the number of nodes with cell identity c. The resulting score ranges from 0 to 1, where a score of 1 indicates that all cells with the same cell identity were connected within the integrated kNN graph, and the lowest possible score indicates a graph where no cells were connected.

#### 4.10.8 Homogeneity and Completeness

Homogeneity was a metric used to evaluate the quality of clustering by measuring how well each cluster contains only data points that belonged to a single class. It assessed whether all samples within a cluster share the same ground‐truth label, making it particularly useful for comparing clustering results to known class labels.

(35) $$h=1-\frac{H(C|K)}{H(C)},c=1-\frac{H(K|C)}{H(K)}$$

where H(C|K) represents the conditional entropy of the classes given the cluster assignments and is defined as:

(36) $$H\left(C|K\right)=-\sum_{c=1}^{\left|C\right|}\sum_{k=1}^{\left|K\right|}\frac{n_{c,k}}{n}\cdot \log\left(\frac{n_{c,k}}{n_{k}}\right)$$

Similarly, H(C) represents the entropy of the classes and is given by:

(37) $$H\left(C\right)=-\sum_{c=1}^{\left|C\right|}\frac{n_{c}}{n}\cdot \log\left(\frac{n_{c}}{n}\right)$$

The conditional entropy of clusters given a class, H(K|C), and the entropy of clusters, H(K), are defined symmetrically. In these formulas, n is the total number of samples, $n_{c}$ and $n_{k}$ are the numbers of samples belonging to class c and cluster k, respectively, $n_{c,k}$ is the number of samples from class c that are assigned to cluster k.

#### 4.10.9 V‐Measure

V‐Measure was a clustering evaluation metric that combined homogeneity and completeness into a single harmonic mean to assess the quality of clustering. It evaluated both how well each cluster contains only a single class (homogeneity) and how well all members of a class were grouped into the same cluster (completeness), providing a balanced view of clustering performance.

(38) $$v=\frac{(1+\beta )\cdot h\cdot c}{\beta \cdot h+c}$$

where β is a weighting factor that adjusts the relative importance of homogeneity versus completeness. By default, β=1, giving equal weight to both.

## Author Contributions

Yanchi Su conceived the study. Yanchi Su and Xiangtao Li drafted the manuscript. Yanchi Su, Qi Qi, Yi Fan, Gaoyang Hao, and Yubo Wang collected and analyzed data. Yanchi Su implemented the algorithm. Yunhe Wang, Xiangtao Li, and Ka‐Chun Wong provided important advice. All authors wrote the manuscript and read and approved the final manuscript.

## Conflicts of Interest

The authors declare no conflict of interest.

## Supporting information


**Supporting File**: advs74805‐sup‐0001‐SuppMat.pdf.

## Data Availability

The data that support the findings of this study are available in the supplementary material of this article.
