# Supplementary material for: PAIR: Reconstructing Single‐Cell Open‐Chromatin Landscapes for Transcription Factor Regulome Mapping
Source: Adv Sci (Weinh). 2026 Mar 14;13(29):e24392. doi: 10.1002/advs.202524392 (PMC13205863; doi:10.1002/advs.202524392)
Supplement: Supplementary file 1 — Supporting File: advs74805‐sup‐0001‐SuppMat.pdf. [file ADVS-13-e24392-s001.pdf]

Supplementary Information "PAIR: Reconstructing Single-Cell Open-Chromatin Landscapes for Transcription Factor Regulome Mapping"

Yanchi Su[1], **Qi Qi**[2], **Yi Fan**[2], **Yubo Wang**[2], **Gaoyang Hao**[2], **Ka-Chun Wong**[3], **Yunhe Wang**[4*], **Xiangtao Li**[2*]

[1]School of Information Science and Technology, Northeast Normal University, Jilin, China

[2]School of Artificial Intelligence, Jilin University, Jilin, China

[3]Department of Computer Science, City University of Hong Kong, Hong Kong SAR

[4]School of Artificial Intelligence, Hebei University of Technology, Tianjin, China

DRAFT

Supplementary Information

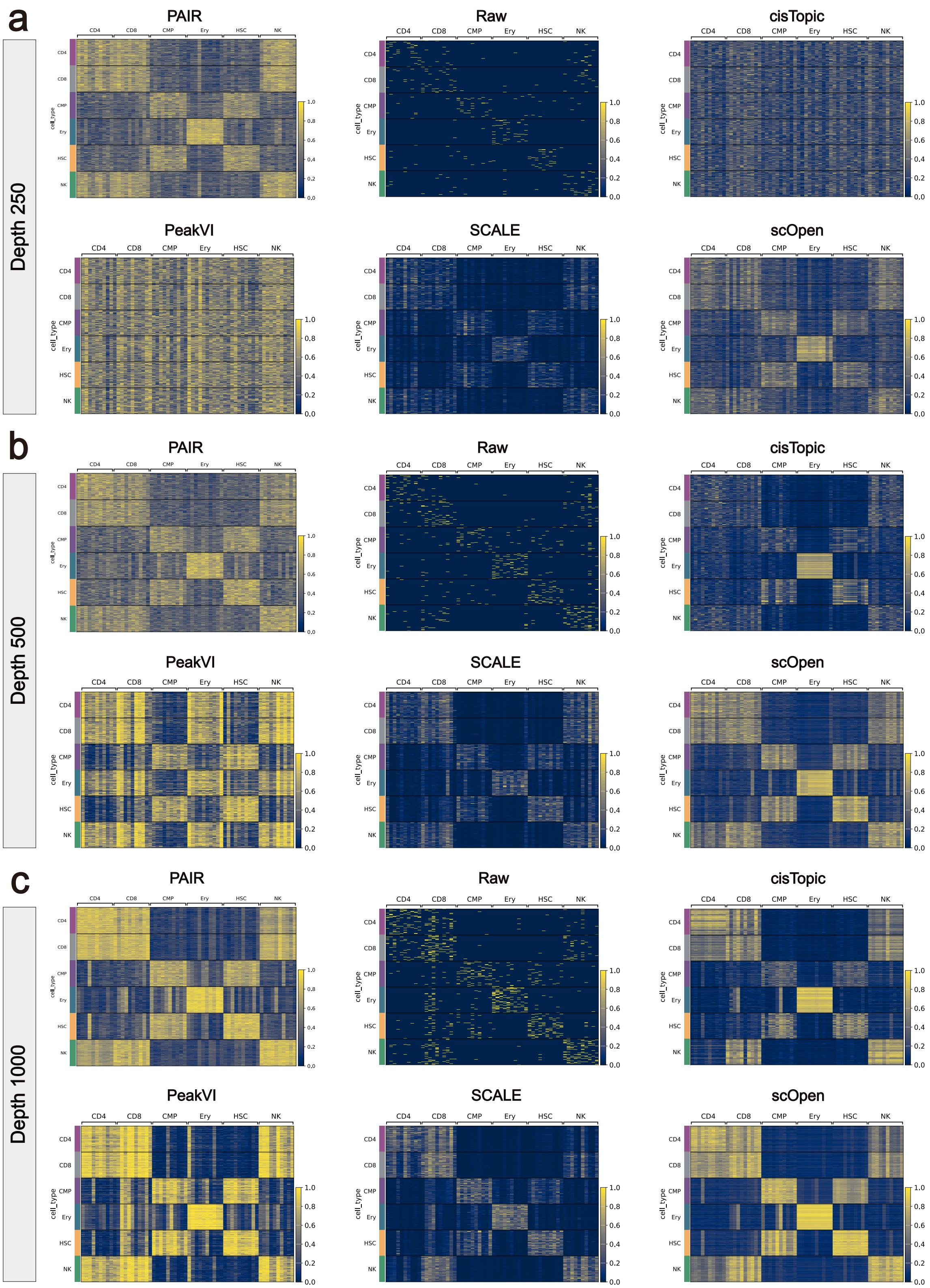

Supplementary Fig. S1. Comparison heatmaps of different methods at varying sequencing depths((a) depth = 250; (b) depth = 500; (c) depth = 1000).

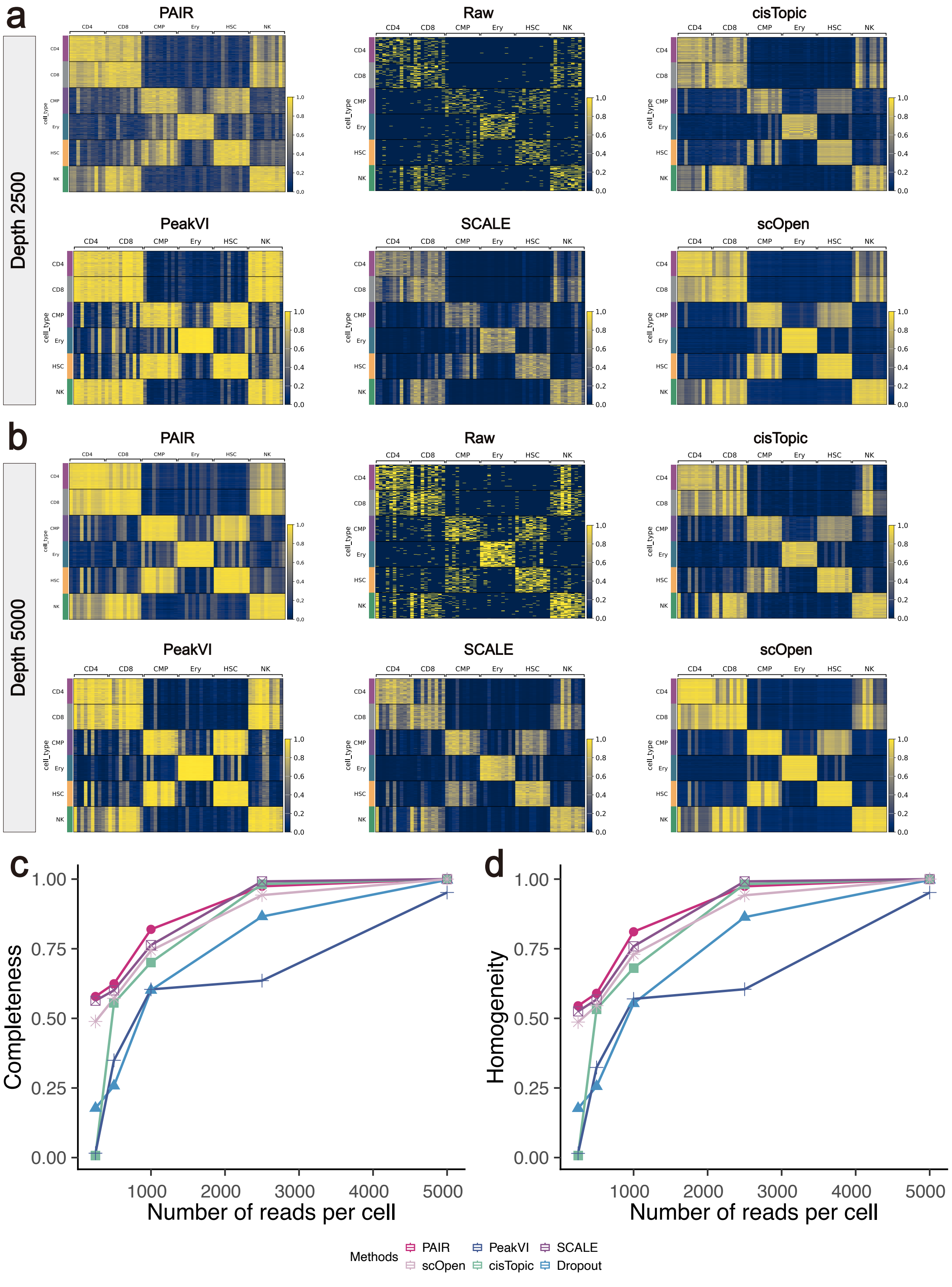

Supplementary Fig. S2. Comparison heatmaps of different methods at varying sequencing depths((a) depth = 2500; (b) depth = 5000). (c&d)Performance under varying sequencing depth, generated by downsampling to different numbers of reads per cell.

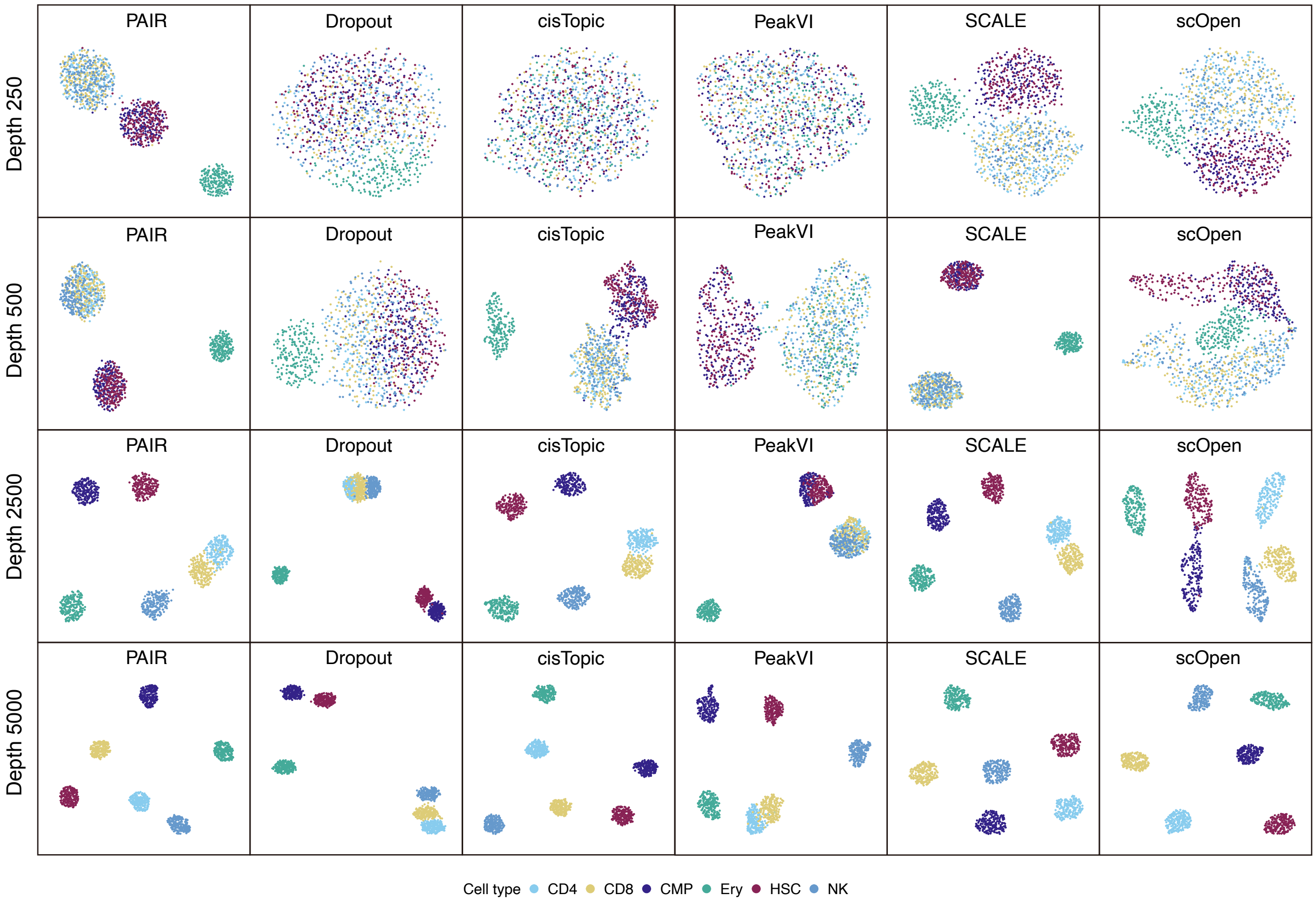

Supplementary Fig. S3. The UMAP plot of different methods under sequencing depth 250, 500, 2500, and 5000.

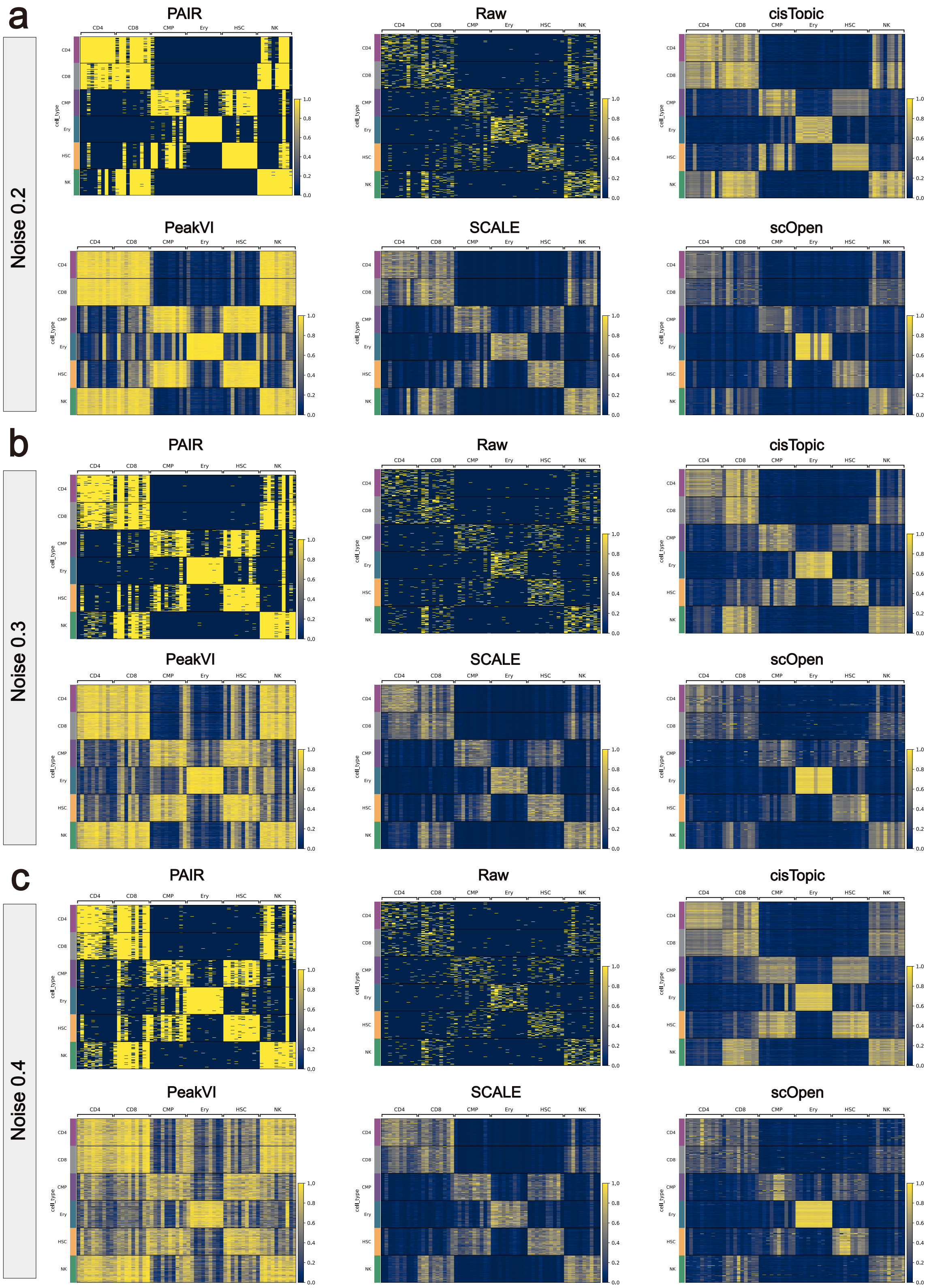

Supplementary Fig. S4. Comparison heatmaps of different methods at varying noise level((a) noise level = 0.2; (b) noise level = 0.3; noise level = 0.4).

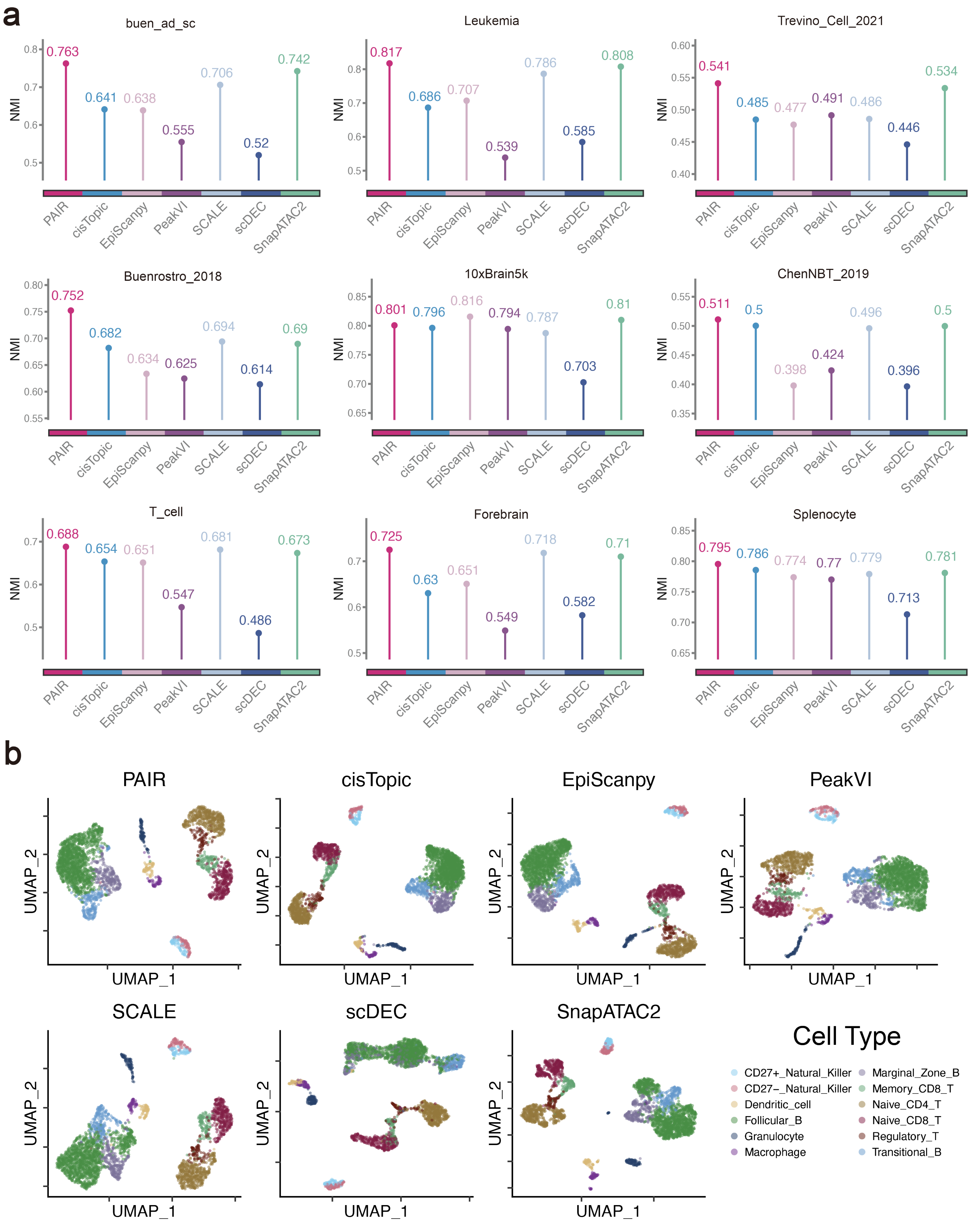

Supplementary Fig. S5. (a) Clustering accuracy measured by NMI on nine scATAC-seq datasets. Bars indicate NMI for each method, and the numeric value above each bar reports the corresponding NMI. Methods compared include PAIR, cisTopic, EpiScanpy, PeakVI, SCALE, scDEC, and SnapATAC2. (b) UMAP visualization on the Splenocyte dataset generated from method-specific representations. Cells are colored by annotated cell type.

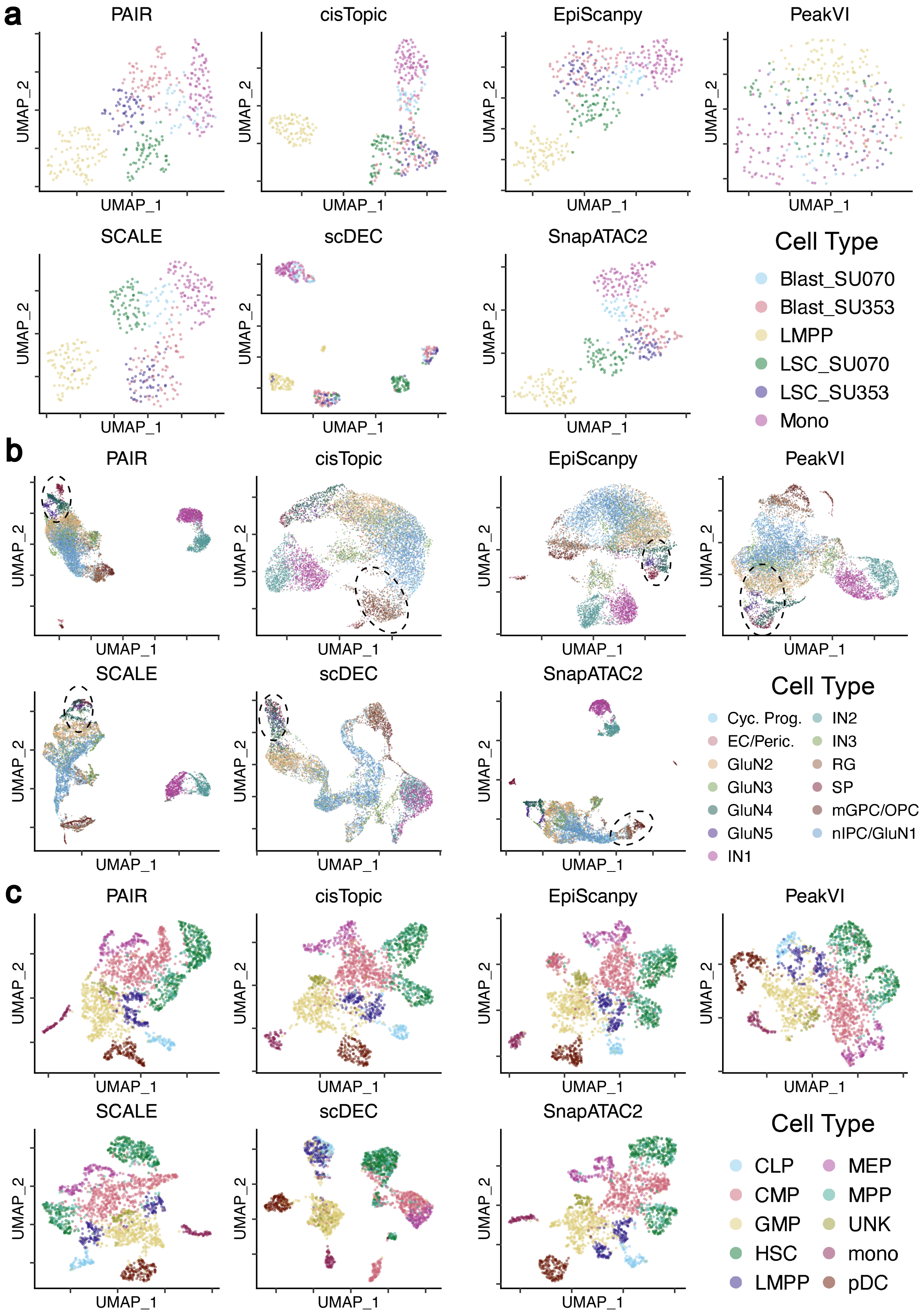

Supplementary Fig. S6. (a-c) UMAP visualization on the Leukemia, Trevino_Cell_2021, and Buenrostro_2018 dataset generated from method-specific representations. Cells are colored by annotated cell type.

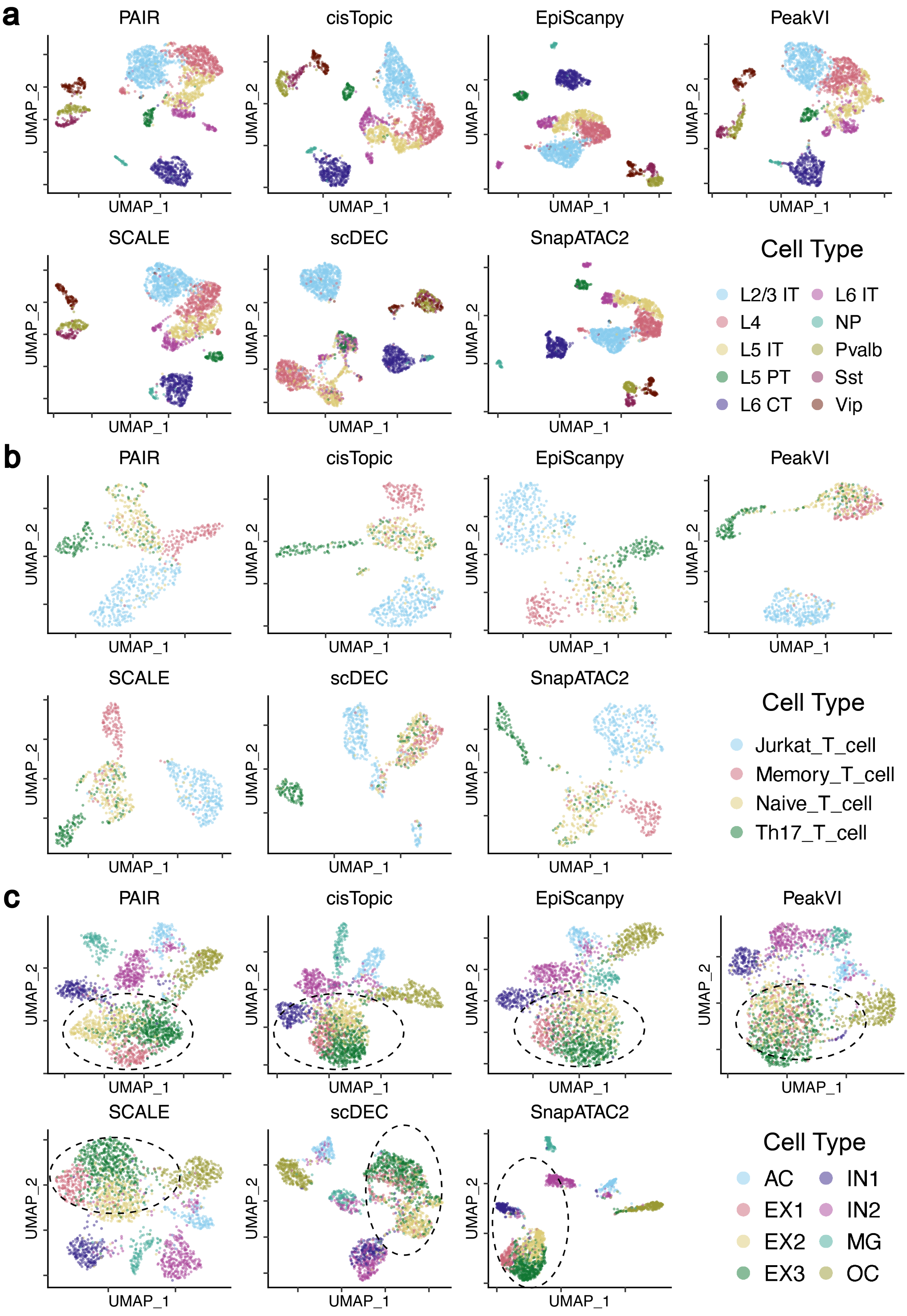

Supplementary Fig. S7. (a-c) UMAP visualization on the 10x_Brain5k, T_cell, and Forebrain dataset generated from method-specific representations. Cells are colored by annotated cell type.

Table S1. The results of Gene Ontology (GO) Biological Process enrichment analysis using GREAT.

cluster	ID	name	score	Binom_Adjp_BH	Binom_Fold_Enrichment	Binom_Region_Set_Coverage
L2/3 IT	GO:0051128	regulation of cellular component organization	307.652656	0	1.287063	0.2794497
L2/3 IT	GO:0032879	regulation of localization	307.652656	0	1.282493	0.2975726
L2/3 IT	GO:0051049	regulation of transport	288.718333	1.91278866871795e-289	1.328629	0.2142909
L2/3 IT	GO:0006996	organelle organization	274.741052	1.81529927425e-275	1.30504	0.2273284
L2/3 IT	GO:0050790	regulation of catalytic activity	270.463213	3.44181153953488e-271	1.291763	0.2381186
L2/3 IT	GO:0035556	intracellular signal transduction	267.94854	1.125797715e-268	1.379828	0.1651948
L2/3 IT	GO:0051641	cellular localization	251.381502	4.154299996e-252	1.333778	0.1869481
L2/3 IT	GO:0006950	response to stress	248.402157	3.96134614615385e-249	1.256807	0.2633148
L2/3 IT	GO:0044267	cellular protein metabolic process	245.729716	1.86330526148148e-246	1.237432	0.2888857
L2/3 IT	GO:0006796	phosphate-containing compound metabolic process	241.239202	5.7649829754386e-242	1.320779	0.1907081
L2/3 IT	GO:0006793	phosphorus metabolic process	238.626623	2.36252608103448e-239	1.31251	0.1958943
L2/3 IT	GO:0044093	positive regulation of molecular function	232.25218	5.59525286833333e-233	1.306929	0.1963264
L2/3 IT	GO:1902578	single-organism localization	231.757069	1.7495677695082e-232	1.284145	0.2177915
L2/3 IT	GO:0044085	cellular component biogenesis	223.311905	4.87635744444444e-224	1.281672	0.2138299
L2/3 IT	GO:1902531	regulation of intracellular signal transduction	219.058857	8.732580346875e-220	1.299267	0.1936181
L2/3 IT	GO:0043085	positive regulation of catalytic activity	218.247667	5.65370995538462e-219	1.339779	0.1615789
L2/3 IT	GO:0043412	macromolecule modification	217.346493	4.50304925e-218	1.231518	0.2719873
L2/3 IT	GO:0022607	cellular component assembly	209.610451	2.45216279e-210	1.284427	0.2002593
L2/3 IT	GO:0044765	single-organism transport	199.676351	2.10692515753425e-200	1.286976	0.1899445
L2/3 IT	GO:0006464	cellular protein modification process	197.398311	3.99658927027027e-198	1.231838	0.2520781
L4	GO:0051128	regulation of cellular component organization	135.487903	3.25159685666667e-136	1.288591	0.2797815
L4	GO:0032879	regulation of localization	130.862262	1.37321471736842e-131	1.270943	0.2948926
L4	GO:0006996	organelle organization	107.09454	8.04378172888889e-108	1.295489	0.2256648
L4	GO:0006950	response to stress	105.205738	6.22676301041667e-106	1.259645	0.2639095
L4	GO:0051049	regulation of transport	95.8799185	1.31850404125e-96	1.293022	0.208548
L4	GO:1902578	single-organism localization	94.6729541	2.12346898596491e-95	1.282042	0.2174349
L4	GO:2000026	regulation of multicellular organismal development	91.6486397	2.2457445487931e-92	1.240597	0.262146
L4	GO:0051641	cellular localization	91.1764681	6.66088421694915e-92	1.311488	0.1838238
L4	GO:0044267	cellular protein metabolic process	91.0315269	9.29978831833333e-92	1.224207	0.2857983
L4	GO:0050790	regulation of catalytic activity	87.7445572	1.80070611111111e-88	1.257219	0.2317508
L4	GO:0035556	intracellular signal transduction	86.5223285	3.00380320625e-87	1.333537	0.1596528
L4	GO:0022008	neurogenesis	85.8372962	1.45446675338462e-86	1.240295	0.2495591
L4	GO:0045595	regulation of cell differentiation	85.6381201	2.3008055e-86	1.24721	0.2398077
L4	GO:0044765	single-organism transport	85.3729217	4.23719392537313e-86	1.291661	0.1906359
L4	GO:0051649	establishment of localization in cell	84.9094865	1.23172434e-85	1.38	0.1303641
L4	GO:0023057	negative regulation of signaling	83.5730579	2.67264980144928e-84	1.322614	0.1624192
L4	GO:0010648	negative regulation of cell communication	82.8116863	1.542814515e-83	1.3215	0.1619351
L4	GO:0043412	macromolecule modification	82.4099482	3.89091584788732e-83	1.221247	0.2697189
L4	GO:1902531	regulation of intracellular signal transduction	81.0519408	8.87276924722222e-82	1.282272	0.1910854
L4	GO:0009968	negative regulation of signal transduction	80.8383594	1.45091030383562e-81	1.337039	0.1482416
L5 IT	GO:0032879	regulation of localization	127.717655	1.915779096e-128	1.25908	0.2921399
L5 IT	GO:0051128	regulation of cellular component organization	126.816525	1.52572094888889e-127	1.27003	0.2757513
L5 IT	GO:0050790	regulation of catalytic activity	120.317919	4.809285635e-121	1.292752	0.2383008
L5 IT	GO:1902578	single-organism localization	105.985846	1.03312853456522e-106	1.289306	0.2186668
L5 IT	GO:0044267	cellular protein metabolic process	102.563532	2.73192227e-103	1.230659	0.2873045
L5 IT	GO:0016192	vesicle-mediated transport	96.8078316	1.55656915615385e-97	1.448168	0.1094957
L5 IT	GO:0044765	single-organism transport	96.316301	4.82724128679245e-97	1.3004	0.1919257
L5 IT	GO:0051641	cellular localization	96.1404528	7.23681134814815e-97	1.309787	0.1835854
L5 IT	GO:0033036	macromolecule localization	94.7354078	1.839044326e-95	1.285786	0.2028299
L5 IT	GO:0044085	cellular component biogenesis	93.7788615	1.66394306375e-94	1.274484	0.2126306

Table S2. The results of Gene Ontology (GO) Biological Process enrichment analysis using GREAT.

cluster	ID	name	score	Binom_Adjp_BH	Binom_Fold_Enrichment	Binom_Region_Set_Coverage
L5 IT	GO:0044085	cellular component biogenesis	93.7788615	1.66394306375e-94	1.274484	0.2126306
L5 IT	GO:0008104	protein localization	91.9193356	1.20410517084746e-92	1.307723	0.1783929
L5 IT	GO:0044093	positive regulation of molecular function	91.8709539	1.34600324833333e-92	1.289948	0.1937756
L5 IT	GO:0051049	regulation of transport	90.5182498	3.03214682222222e-91	1.275276	0.2056857
L5 IT	GO:0022607	cellular component assembly	89.990545	1.02200972671875e-90	1.280307	0.1996171
L5 IT	GO:0043412	macromolecule modification	88.396075	4.0172139e-89	1.221965	0.2698773
L5 IT	GO:0006464	cellular protein modification process	87.4669372	3.41242233134328e-88	1.232229	0.2521581
L5 IT	GO:0043085	positive regulation of catalytic activity	85.8161877	1.5269059575e-86	1.319895	0.1591809
L5 IT	GO:0009653	anatomical structure morphogenesis	85.6777175	2.10030567681159e-86	1.20686	0.2888947
L5 IT	GO:0006793	phosphorus metabolic process	84.5933182	2.55083147e-85	1.278962	0.1908873
L5 IT	GO:0006796	phosphate-containing compound metabolic process	83.9527577	1.11491641366197e-84	1.283587	0.1853378
L5 PT	GO:0051128	regulation of cellular component organization	245.600313	2.510074259e-246	1.321555	0.2869387
L5 PT	GO:0006996	organelle organization	228.808137	1.55547359333333e-229	1.358543	0.2366483
L5 PT	GO:0051641	cellular localization	228.540695	2.879421545e-229	1.411096	0.1977853
L5 PT	GO:0035556	intracellular signal transduction	202.959004	1.09899471868421e-203	1.425647	0.1706805
L5 PT	GO:0050790	regulation of catalytic activity	200.536947	2.90437535756098e-201	1.322853	0.2438495
L5 PT	GO:0044267	cellular protein metabolic process	194.344982	4.51875215e-195	1.271367	0.2968079
L5 PT	GO:0051049	regulation of transport	193.397686	4.00233751627907e-194	1.34516	0.2169571
L5 PT	GO:1902578	single-organism localization	184.225732	5.94658792291667e-185	1.326	0.2248902
L5 PT	GO:1902589	single-organism organelle organization	183.109072	7.77907978571429e-184	1.426274	0.155735
L5 PT	GO:0051649	establishment of localization in cell	180.274537	5.314508584e-181	1.460869	0.1380035
L5 PT	GO:0044093	positive regulation of molecular function	176.78966	1.62308146e-177	1.34446	0.2019644
L5 PT	GO:0006796	phosphate-containing compound metabolic process	171.545944	2.8448286625e-172	1.347555	0.1945743
L5 PT	GO:0006793	phosphorus metabolic process	168.071586	8.48036327068965e-169	1.336886	0.1995325
L5 PT	GO:0044765	single-organism transport	163.265388	5.42765091186441e-164	1.334183	0.1969117
L5 PT	GO:0033036	macromolecule localization	162.063298	8.64375678833333e-163	1.319364	0.2081267
L5 PT	GO:0006950	response to stress	159.70163	1.9877891283871e-160	1.26397	0.2648156
L5 PT	GO:0043085	positive regulation of catalytic activity	158.869108	1.35173619109375e-159	1.372764	0.165557
L5 PT	GO:0044085	cellular component biogenesis	156.846368	1.42440083584615e-157	1.303105	0.2174057
L5 PT	GO:0008104	protein localization	154.924326	1.19034852791045e-155	1.34136	0.1829815
L5 PT	GO:0046907	intracellular transport	152.133913	7.34660693768116e-153	1.489952	0.1087264
L6 CT	GO:0051128	regulation of cellular component organization	159.259448	5.50240406896552e-160	1.337164	0.2903277
L6 CT	GO:0006996	organelle organization	112.75337	1.76453367106383e-113	1.326323	0.2310357
L6 CT	GO:0044093	positive regulation of molecular function	108.230958	5.87545959e-109	1.35084	0.2029228
L6 CT	GO:0050790	regulation of catalytic activity	106.125832	7.48458223333333e-107	1.304968	0.2405527
L6 CT	GO:0035556	intracellular signal transduction	103.810142	1.54831041037037e-104	1.394616	0.1669653
L6 CT	GO:1902578	single-organism localization	97.6654208	2.16062391754386e-98	1.308234	0.2218771
L6 CT	GO:0051049	regulation of transport	97.4930976	3.21293824310345e-98	1.317892	0.2125592
L6 CT	GO:1902531	regulation of intracellular signal transduction	93.6853373	2.06377672152542e-94	1.327236	0.197786
L6 CT	GO:0044765	single-organism transport	92.8604242	1.379036736e-93	1.327695	0.1959543
L6 CT	GO:0051641	cellular localization	92.7489463	1.7825992042623e-93	1.338088	0.1875523
L6 CT	GO:0022607	cellular component assembly	92.2283002	5.9115284516129e-93	1.315557	0.2051129
L6 CT	GO:0044085	cellular component biogenesis	92.1290891	7.42866642222222e-93	1.30222	0.217258
L6 CT	GO:0043085	positive regulation of catalytic activity	91.8462156	1.4249000871875e-92	1.368593	0.165054
L6 CT	GO:0006950	response to stress	91.815639	1.52883648076923e-92	1.260299	0.2640465
L6 CT	GO:0048584	positive regulation of response to stimulus	87.4766699	3.33679966666667e-88	1.297098	0.2131565
L6 CT	GO:0044267	cellular protein metabolic process	85.86676	1.359064245e-86	1.233887	0.288058
L6 CT	GO:1902589	single-organism organelle organization	85.3973424	4.00550774927536e-86	1.376686	0.1503206
L6 CT	GO:0070887	cellular response to chemical stimulus	85.149967	7.07999578e-86	1.256929	0.2533747
L6 CT	GO:0006796	phosphate-containing compound metabolic process	84.0710986	8.4898763369863e-85	1.31547	0.1899415

Table S3. The results of Gene Ontology (GO) Biological Process enrichment analysis using GREAT.

cluster	ID	name	score	Binom_Adjp_BH	Binom_Fold_Enrichment	Binom_Region_Set_Coverage
L6 CT	GO:0006793	phosphorus metabolic process	82.7182824	1.91301151621622e-83	1.306509	0.1949986
L6 IT	GO:0051128	regulation of cellular component organization	155.268757	5.385712784375e-156	1.309497	0.2843205
L6 CT	GO:0006793	phosphorus metabolic process	82.7182824	1.91301151621622e-83	1.306509	0.1949986
L6 IT	GO:0051128	regulation of cellular component organization	155.268757	5.385712784375e-156	1.309497	0.2843205
L6 IT	GO:0032879	regulation of localization	148.447493	3.568671645e-149	1.289049	0.2990936
L6 IT	GO:0044267	cellular protein metabolic process	121.673243	2.1220555493617e-122	1.259965	0.2941461
L6 IT	GO:0050790	regulation of catalytic activity	118.071267	8.48658323333333e-119	1.299537	0.2395516
L6 IT	GO:0044093	positive regulation of molecular function	114.432079	3.69760987037037e-115	1.335575	0.2006297
L6 IT	GO:0051049	regulation of transport	110.153435	7.0236849375e-111	1.314715	0.2120468
L6 IT	GO:0043412	macromolecule modification	109.715678	1.92452027649123e-110	1.256031	0.2774011
L6 IT	GO:0043085	positive regulation of catalytic activity	109.046304	8.98867731034483e-110	1.374411	0.1657556
L6 IT	GO:0006796	phosphate-containing compound metabolic process	106.141086	7.2262657220339e-107	1.330789	0.1921533
L6 IT	GO:0048584	positive regulation of response to stimulus	105.69789	2.00498006409836e-106	1.30445	0.2143648
L6 IT	GO:0044085	cellular component biogenesis	104.232163	5.85918534193548e-105	1.299395	0.2167866
L6 IT	GO:0006793	phosphorus metabolic process	103.525905	2.97916764444444e-104	1.319898	0.196997
L6 IT	GO:1902531	regulation of intracellular signal transduction	103.310704	4.889855403125e-104	1.319852	0.1966856
L6 IT	GO:0006464	cellular protein modification process	101.777736	1.66825948046154e-102	1.259216	0.2576806
L6 IT	GO:0022607	cellular component assembly	100.198656	6.32912805e-101	1.306107	0.2036396
L6 IT	GO:0051246	regulation of protein metabolic process	99.5792594	2.63475715373134e-100	1.255205	0.2585801
L6 IT	GO:0019220	regulation of phosphate metabolic process	98.6514199	2.2314138e-99	1.303461	0.2034321
L6 IT	GO:0051174	regulation of phosphorus metabolic process	97.9966684	1.00770083014493e-98	1.301977	0.2036742
L6 IT	GO:0032268	regulation of cellular protein metabolic process	96.9951098	1.011323676e-97	1.26296	0.2434957
L6 IT	GO:0051641	cellular localization	94.1509295	7.06432270422535e-95	1.316611	0.1845419
NP	GO:0006950	response to stress	44.6813922	2.08260935473684e-45	1.308541	0.2741538
NP	GO:0002376	immune system process	42.0406076	9.107358575e-43	1.41493	0.1770903
NP	GO:0051128	regulation of cellular component organization	40.4698843	3.38934424888889e-41	1.286418	0.2793096
NP	GO:0048584	positive regulation of response to stimulus	38.9323836	1.16846685083333e-39	1.336804	0.2196817
NP	GO:0035556	intracellular signal transduction	38.1012644	7.92018998571429e-39	1.405228	0.1682358
NP	GO:0006996	organelle organization	37.23498	5.82129995740741e-38	1.317121	0.2294329
NP	GO:0044267	cellular protein metabolic process	36.6906734	2.03857465625e-37	1.259307	0.2939924
NP	GO:0006793	phosphorus metabolic process	34.5779693	2.64259542105263e-35	1.336714	0.1995068
NP	GO:0006796	phosphate-containing compound metabolic process	34.4330705	3.68917688644068e-35	1.342904	0.1939027
NP	GO:0002682	regulation of immune system process	34.0279435	9.37684042166667e-35	1.452554	0.1309124
NP	GO:0044093	positive regulation of molecular function	33.887009	1.29715226245902e-34	1.331831	0.2000672
NP	GO:0051246	regulation of protein metabolic process	33.6029617	2.49481465483871e-34	1.269867	0.2616005
NP	GO:0051641	cellular localization	33.3277313	4.70184904444444e-34	1.343413	0.1882986
NP	GO:0050790	regulation of catalytic activity	32.7905246	1.61985225769231e-33	1.286599	0.2371666
NP	GO:0043412	macromolecule modification	32.3014749	4.99488021666667e-33	1.252493	0.2766196
NP	GO:0070887	cellular response to chemical stimulus	32.0328368	9.27178175e-33	1.267155	0.255436
NP	GO:1902578	single-organism localization	31.185273	6.52720128732394e-32	1.294629	0.2195696
NP	GO:0071702	organic substance transport	30.7863579	1.63546809066667e-31	1.347865	0.1729433
NP	GO:0043085	positive regulation of catalytic activity	30.0903386	8.12197052658228e-31	1.356872	0.1636404
NP	GO:0032268	regulation of cellular protein metabolic process	29.2762014	5.29417939382716e-30	1.262688	0.2434432
Pvalb	GO:0006996	organelle organization	204.685133	2.06474969555556e-205	1.392138	0.2425002
Pvalb	GO:0006950	response to stress	197.945382	1.13401355128205e-198	1.341192	0.2809943
Pvalb	GO:0050790	regulation of catalytic activity	178.367629	4.2891462326087e-179	1.351925	0.2492085
Pvalb	GO:0051128	regulation of cellular component organization	176.661429	2.18057592617021e-177	1.314101	0.2853202
Pvalb	GO:0044093	positive regulation of molecular function	161.197261	6.349490378e-162	1.380589	0.2073916
Pvalb	GO:0006796	phosphate-containing compound metabolic process	154.340607	4.56449558653846e-155	1.381398	0.1994608
Pvalb	GO:0035556	intracellular signal transduction	152.976661	1.05521157396226e-153	1.425941	0.1707157

Table S4. The results of Gene Ontology (GO) Biological Process enrichment analysis using GREAT.

cluster	ID	name	score	Binom_Adjp_BH	Binom_Fold_Enrichment	Binom_Region_Set_Coverage
Pvalb	GO:1902589	single-organism organelle organization	152.645628	2.26137216148148e-153	1.449785	0.1583022
Pvalb	GO:0006793	phosphorus metabolic process	149.872871	1.34007428210526e-150	1.36791	0.2041629
Pvalb	GO:0071702	organic substance transport	149.596521	2.53208897586207e-150	1.403552	0.1800884
Pvalb	GO:1902578	single-organism localization	146.491272	3.22647119666667e-147	1.335385	0.2264819
Pvalb	GO:1902531	regulation of intracellular signal transduction	145.693478	2.02545132196721e-146	1.362876	0.2030971
Pvalb	GO:0043085	positive regulation of catalytic activity	143.171783	6.73313496935484e-144	1.40956	0.1699947
Pvalb	GO:0051641	cellular localization	140.597849	2.52435871111111e-141	1.370046	0.1920316
Pvalb	GO:0051049	regulation of transport	138.680178	2.088439959375e-139	1.336579	0.2155732
Pvalb	GO:0044765	single-organism transport	134.453644	3.51848728e-135	1.35018	0.1992728
Pvalb	GO:0048584	positive regulation of response to stimulus	133.778302	1.66608845666667e-134	1.326491	0.2179869
Pvalb	GO:0007010	cytoskeleton organization	133.051644	8.87883563731343e-134	1.517905	0.1154823
Pvalb	GO:0044267	cellular protein metabolic process	132.432875	3.690839075e-133	1.257876	0.2936585
Pvalb	GO:0070887	cellular response to chemical stimulus	132.427908	3.73329076521739e-133	1.284624	0.2589574
Sst	GO:0006950	response to stress	83.7275815	1.87248554166667e-84	1.33018	0.2786874
Sst	GO:0050790	regulation of catalytic activity	80.3225226	4.75858030232558e-81	1.351815	0.2491883
Sst	GO:0051128	regulation of cellular component organization	75.4199798	3.80207064285714e-76	1.305507	0.2834542
Sst	GO:0006996	organelle organization	67.83337	1.46767546961538e-68	1.334555	0.2324698
Sst	GO:0044093	positive regulation of molecular function	65.6343059	2.32110126433962e-66	1.361276	0.2044905
Sst	GO:0006796	phosphate-containing compound metabolic process	62.2291774	5.8995998875e-63	1.360252	0.1964076
Sst	GO:1902531	regulation of intracellular signal transduction	61.1364853	7.30322516e-62	1.349974	0.2011744
Sst	GO:1902589	single-organism organelle organization	61.094783	8.03927714754099e-62	1.422943	0.1553713
Sst	GO:0048584	positive regulation of response to stimulus	60.4466497	3.57561161774194e-61	1.327181	0.2181002
Sst	GO:0006793	phosphorus metabolic process	59.9326919	1.16763755777778e-60	1.346036	0.2008981
Sst	GO:0043085	positive regulation of catalytic activity	59.7376074	1.829753743125e-60	1.393708	0.1680829
Sst	GO:0007010	cytoskeleton organization	58.5579687	2.76714141846154e-59	1.511906	0.1150259
Sst	GO:0035556	intracellular signal transduction	57.8620981	1.37373161298507e-58	1.388948	0.1662867
Sst	GO:0023056	positive regulation of signaling	57.7784549	1.665501915e-58	1.346067	0.1948187
Sst	GO:0044710	single-organism metabolic process	57.1089139	7.78190824782609e-58	1.260181	0.2812435
Sst	GO:0010647	positive regulation of cell communication	56.8864673	1.29877136338028e-57	1.343547	0.1941969
Sst	GO:1902578	single-organism localization	56.1774612	6.64567119027778e-57	1.308776	0.2219689
Sst	GO:0033554	cellular response to stress	54.7969178	1.59618116093333e-55	1.40643	0.1492228
Sst	GO:0044085	cellular component biogenesis	52.5351748	2.9162531e-53	1.301471	0.217133
Sst	GO:0044765	single-organism transport	51.9813702	1.04382999628205e-52	1.323746	0.1953713
Vip	GO:0006950	response to stress	82.4017159	3.96537319574468e-83	1.33706	0.2801286
Vip	GO:0050790	regulation of catalytic activity	76.3285969	4.692487492e-77	1.352749	0.2493605
Vip	GO:0044093	positive regulation of molecular function	72.6206354	2.39532601e-73	1.391905	0.2090916
Vip	GO:0006996	organelle organization	72.5171266	3.03999894423077e-73	1.356421	0.2362786
Vip	GO:0032879	regulation of localization	69.9602979	1.095726296e-70	1.289207	0.2991303
Vip	GO:1902531	regulation of intracellular signal transduction	68.1543956	7.00816565166667e-69	1.381033	0.2058028
Vip	GO:0048584	positive regulation of response to stimulus	67.3316853	4.6592355983871e-68	1.35597	0.2228313
Vip	GO:0043085	positive regulation of catalytic activity	67.3300393	4.67692817777778e-68	1.431354	0.172623
Vip	GO:0051128	regulation of cellular component organization	65.5211214	3.01216406615385e-66	1.292547	0.2806402
Vip	GO:0006796	phosphate-containing compound metabolic process	63.0417055	9.08436375e-64	1.373187	0.1982752
Vip	GO:0006793	phosphorus metabolic process	61.6363247	2.31033658493151e-62	1.36127	0.2031718
Vip	GO:0045595	regulation of cell differentiation	59.6589038	2.1932907e-60	1.302214	0.2503837
Vip	GO:0023056	positive regulation of signaling	59.4372096	3.65418426153846e-60	1.361365	0.1970328
Vip	GO:0009968	negative regulation of signal transduction	59.2506243	5.6153349443038e-60	1.42445	0.1579332
Vip	GO:0010647	positive regulation of cell communication	58.7983672	1.590863043e-59	1.359628	0.1965212
Vip	GO:0051246	regulation of protein metabolic process	58.1363343	7.30576525853659e-59	1.284951	0.264708
Vip	GO:0033554	cellular response to stress	57.5818233	2.619248225e-58	1.429288	0.151648
Vip	GO:0032268	regulation of cellular protein metabolic process	57.5248681	2.98628946e-58	1.296033	0.2498721
Vip	GO:0044267	cellular protein metabolic process	56.8046665	1.56795480574713e-57	1.258775	0.2938683
Vip	GO:1902589	single-organism organelle organization	56.4591646	3.47404435e-57	1.417625	0.1547906

Table S5. The results of Gene Ontology (GO) Cellular Components enrichment analysis using GREAT.

cluster	ID	name	score	Binom_Adjp_BH	Binom_Fold_Enrichment	Binom_Region_Set_Coverage
L2/3 IT	GO:0005856	cytoskeleton	294.779525	1.66140179333333e-295	1.346967	0.2023626
L2/3 IT	GO:0043234	protein complex	284.802654	1.57523811789474e-285	1.25965	0.2877332
L2/3 IT	GO:0070062	extracellular exosome	243.214603	6.10094584e-244	1.280567	0.2300223
L2/3 IT	GO:1903561	extracellular vesicle	239.889966	1.2883510773913e-240	1.276997	0.2314053
L2/3 IT	GO:0043230	extracellular organelle	239.843852	1.4326772075e-240	1.276734	0.2316502
L2/3 IT	GO:0042995	cell projection	238.037909	9.164119888e-239	1.261545	0.2482317
L2/3 IT	GO:0097708	intracellular vesicle	236.063988	8.63002436153846e-237	1.335604	0.1755384
L2/3 IT	GO:0031410	cytoplasmic vesicle	233.257038	5.53301306962963e-234	1.334261	0.1747173
L2/3 IT	GO:0097458	neuron part	230.293509	5.087340335e-231	1.293961	0.2064539
L2/3 IT	GO:0005654	nucleoplasm	228.469012	3.39616274344828e-229	1.255005	0.2485054
L2/3 IT	GO:0031090	organelle membrane	222.701027	1.99054836129032e-223	1.281413	0.2131672
L2/3 IT	GO:0044430	cytoskeletal part	201.352746	4.43868126625e-202	1.345504	0.1467406
L2/3 IT	GO:0098805	whole membrane	184.983021	1.03987086823529e-185	1.35277	0.132104
L2/3 IT	GO:0045202	synapse	183.025961	9.41973384e-184	1.321772	0.149449
L2/3 IT	GO:0098588	bounding membrane of organelle	178.345795	4.51029148216216e-179	1.313161	0.1519124
L2/3 IT	GO:0030054	cell junction	171.840366	1.44422282052632e-172	1.27745	0.175063
L2/3 IT	GO:0044456	synapse part	167.403304	3.9508971425e-168	1.350896	0.1217028
L2/3 IT	GO:0014069	postsynaptic density	154.652479	2.22597627488372e-155	1.589586	0.05317295
L2/3 IT	GO:0099572	postsynaptic specialization	152.523356	2.99670217e-153	1.583169	0.05328819
L2/3 IT	GO:0032279	asymmetric synapse	152.002455	9.94362565777778e-153	1.573333	0.05441187
L4	GO:0097458	neuron part	99.0251428	9.43750581052632e-100	1.299284	0.2073032
L4	GO:0005856	cytoskeleton	97.4154165	3.84223158e-98	1.30804	0.1965144
L4	GO:0097708	intracellular vesicle	90.3257585	4.72325623e-91	1.321556	0.173692
L4	GO:0031410	cytoplasmic vesicle	90.0215012	9.5169730173913e-91	1.321679	0.1730696
L4	GO:0005654	nucleoplasm	90.0021579	9.95043600833333e-91	1.248103	0.2471386
L4	GO:0031090	organelle membrane	88.0158489	9.641644936e-89	1.274225	0.2119714
L4	GO:0042995	cell projection	87.9450507	1.13487836e-88	1.246149	0.2452021
L4	GO:0043234	protein complex	86.6238189	2.37783141037037e-87	1.221206	0.2789516
L4	GO:0098588	bounding membrane of organelle	83.336012	4.613048165e-84	1.332843	0.1541893
L4	GO:0070062	extracellular exosome	70.9355856	1.159883494e-71	1.233784	0.221619
L4	GO:0043230	extracellular organelle	69.2601949	5.49294254193548e-70	1.229452	0.2230713
L4	GO:1903561	extracellular vesicle	69.1835647	6.55292586666667e-70	1.22948	0.2227947
L4	GO:0045202	synapse	68.3750386	4.216590576e-69	1.304366	0.1474809
L4	GO:0098805	whole membrane	67.687871	2.05177138833333e-68	1.329995	0.12988
L4	GO:0005783	endoplasmic reticulum	66.1185669	7.61084898378378e-67	1.291526	0.1524603
L4	GO:0044463	cell projection part	63.8097303	1.54977863526316e-64	1.306956	0.1370379
L4	GO:0044430	cytoskeletal part	63.056264	8.7848841948718e-64	1.298073	0.1415678
L4	GO:0005794	Golgi apparatus	62.7151273	1.9269601505e-63	1.291191	0.1457865
L4	GO:0043005	neuron projection	60.5376231	2.89985943902439e-61	1.262336	0.1646668
L4	GO:0005768	endosome	60.2749355	5.30963326666667e-61	1.410936	0.08447733
L5 IT	GO:0097458	neuron part	107.972068	1.066428902e-108	1.302774	0.2078601
L5 IT	GO:0042995	cell projection	104.786171	1.63617087333333e-105	1.260719	0.2480691
L5 IT	GO:0097708	intracellular vesicle	99.877783	1.32500334521739e-100	1.327693	0.1744986
L5 IT	GO:0031410	cytoplasmic vesicle	99.2859525	5.17663389833333e-100	1.327387	0.1738171
L5 IT	GO:0043234	protein complex	89.4449285	3.5898102856e-90	1.217709	0.2781528
L5 IT	GO:0098588	bounding membrane of organelle	87.7522904	1.76892562e-88	1.330826	0.153956
L5 IT	GO:0005856	cytoskeleton	87.5859146	2.59468976148148e-88	1.282033	0.1926073
L5 IT	GO:0031090	organelle membrane	86.3768205	4.199324965e-87	1.262776	0.2100669
L5 IT	GO:0045202	synapse	83.8581817	1.3861756662069e-84	1.327478	0.1500941
L5 IT	GO:0044459	plasma membrane part	83.7856919	1.63797829066667e-84	1.208192	0.2804894

Table S6. The results of Gene Ontology (GO) Cellular Components enrichment analysis using GREAT.

cluster	ID	name	score	Binom_Adjp_BH	Binom_Fold_Enrichment	Binom_Region_Set_Coverage
L5 IT	GO:0005794	Golgi apparatus	77.0012106	9.97216269677419e-78	1.313534	0.1483092
L5 IT	GO:0005654	nucleoplasm	74.9340119	1.164094036875e-75	1.218548	0.2412864
L5 IT	GO:0044463	cell projection part	74.7658993	1.71435469333333e-75	1.322528	0.1386707
L5 IT	GO:0070062	extracellular exosome	72.0470665	8.97291372352941e-73	1.228009	0.2205816
L5 IT	GO:1903561	extracellular vesicle	70.7412452	1.814490832e-71	1.224609	0.2219121
L5 IT	GO:0043230	extracellular organelle	70.2424177	5.72245434444444e-71	1.223599	0.2220095
L5 IT	GO:0098805	whole membrane	68.9539501	1.11185957473684e-69	1.322311	0.1291296
L5 IT	GO:0043005	neuron projection	68.9539501	1.11185957473684e-69	1.27153	0.1658662
L5 IT	GO:0030054	cell junction	66.605966	2.47761573230769e-67	1.258885	0.172519
L5 IT	GO:0005768	endosome	57.4414518	3.61866371658537e-58	1.387583	0.08307912
L5 PT	GO:0005856	cytoskeleton	231.977474	1.05323697111111e-232	1.396178	0.2097559
L5 PT	GO:0097458	neuron part	211.968376	1.07553397368421e-212	1.363933	0.2176182
L5 PT	GO:0045202	synapse	207.632332	2.33167242e-208	1.445233	0.1634084
L5 PT	GO:0042995	cell projection	206.600171	2.5108952e-207	1.313669	0.258488
L5 PT	GO:0031090	organelle membrane	190.19814	6.33665668086957e-191	1.334994	0.2220806
L5 PT	GO:0044456	synapse part	186.730354	1.8605689025e-187	1.482039	0.1335175
L5 PT	GO:0043234	protein complex	175.728811	1.86719130230769e-176	1.261134	0.288072
L5 PT	GO:0097708	intracellular vesicle	173.650532	2.23598024222222e-174	1.36996	0.1800538
L5 PT	GO:0031410	cytoplasmic vesicle	171.953355	1.113383315e-172	1.368885	0.1792511
L5 PT	GO:0098588	bounding membrane of organelle	169.922661	1.19491956137931e-170	1.395187	0.1614015
L5 PT	GO:0098805	whole membrane	168.072742	8.4578032e-169	1.434699	0.1401048
L5 PT	GO:0014069	postsynaptic density	165.864307	1.36676182322581e-166	1.800567	0.06023044
L5 PT	GO:0044463	cell projection part	164.551582	2.80813638875e-165	1.41209	0.1480616
L5 PT	GO:0099572	postsynaptic specialization	163.797214	1.59509401333333e-164	1.792226	0.06032488
L5 PT	GO:0032279	asymmetric synapse	161.867983	1.35524135352941e-162	1.775031	0.06138735
L5 PT	GO:0098984	neuron to neuron synapse	160.162915	6.8720256e-161	1.766612	0.06164707
L5 PT	GO:0070062	extracellular exosome	154.187258	6.49743581666667e-155	1.286039	0.2310053
L5 PT	GO:1903561	extracellular vesicle	152.832289	1.47133377243243e-153	1.283128	0.2325164
L5 PT	GO:0043230	extracellular organelle	152.750122	1.77778122789474e-153	1.282809	0.2327525
L5 PT	GO:0005654	nucleoplasm	151.883854	1.30660913025641e-152	1.266429	0.2507673
L6 CT	GO:0005856	cytoskeleton	110.537594	2.90005221578947e-111	1.353876	0.2034006
L6 CT	GO:0097458	neuron part	94.3765266	4.20216786e-95	1.314007	0.2096524
L6 CT	GO:0045202	synapse	91.7309624	1.85796520608696e-92	1.381955	0.1562537
L6 CT	GO:0043234	protein complex	90.4404009	3.62743066583333e-91	1.243117	0.2839565
L6 CT	GO:0031090	organelle membrane	89.1601464	6.915978608e-90	1.296908	0.2157448
L6 CT	GO:0042995	cell projection	85.1801364	6.60486039230769e-86	1.260363	0.247999
L6 CT	GO:0005654	nucleoplasm	77.8566426	1.39109711481481e-78	1.247622	0.2470434
L6 CT	GO:0098588	bounding membrane of organelle	76.097399	7.9909973e-77	1.341739	0.1552184
L6 CT	GO:0044459	plasma membrane part	76.0544291	8.82207745517241e-77	1.22004	0.2832398
L6 CT	GO:0030054	cell junction	75.1170125	7.63813871333333e-76	1.306403	0.1790308
L6 CT	GO:0005783	endoplasmic reticulum	72.5962525	2.53365514387097e-73	1.329396	0.1569307
L6 CT	GO:0044456	synapse part	72.0633392	8.6429266e-73	1.384787	0.1247561
L6 CT	GO:0044430	cytoskeletal part	71.0844685	8.23249621764706e-72	1.341452	0.1462987
L6 CT	GO:0031410	cytoplasmic vesicle	69.8920566	1.282163432e-70	1.303343	0.1706686
L6 CT	GO:0098590	plasma membrane region	69.5269437	2.97205147555556e-70	1.338852	0.1450245
L6 CT	GO:0097708	intracellular vesicle	69.1611252	6.90040827567568e-70	1.300975	0.1709871
L6 CT	GO:0044463	cell projection part	68.8539262	1.39982530947368e-69	1.343629	0.1408832
L6 CT	GO:0070062	extracellular exosome	68.7674806	1.70812405076923e-69	1.247192	0.2240274
L6 CT	GO:1903561	extracellular vesicle	67.4209513	3.793574939e-68	1.243314	0.2253016
L6 CT	GO:0043230	extracellular organelle	67.1631239	6.86872377560976e-68	1.242622	0.2254609
L6 IT	GO:0005856	cytoskeleton	115.220975	6.01208128888889e-116	1.336123	0.2007335

Table S7. The results of Gene Ontology (GO) Cellular Components enrichment analysis using GREAT.

cluster	ID	name	score	Binom_Adjp_BH	Binom_Fold_Enrichment	Binom_Region_Set_Coverage
L6 IT	GO:0043234	protein complex	102.008408	9.80826806666667e-103	1.240621	0.2833864
L6 IT	GO:0097708	intracellular vesicle	91.8016455	1.57889963e-92	1.324348	0.174059
L6 IT	GO:0031410	cytoplasmic vesicle	90.2029631	6.26667164869565e-91	1.3221	0.1731248
L6 IT	GO:0030054	cell junction	89.3728459	4.23793344333333e-90	1.311778	0.1797675
L6 IT	GO:0098805	whole membrane	85.0980448	7.97912440769231e-86	1.372137	0.1339953
L6 IT	GO:0031090	organelle membrane	82.7886805	1.626745175e-83	1.265735	0.2105591
L6 IT	GO:0098588	bounding membrane of organelle	79.6354469	2.3150110537931e-80	1.325161	0.1533006
L6 IT	GO:0097458	neuron part	78.1852996	6.52680130666667e-79	1.26483	0.201806
L6 IT	GO:0005654	nucleoplasm	77.0002973	9.99315736774193e-78	1.229007	0.2433573
L6 IT	GO:0045202	synapse	76.8018153	1.578282330625e-77	1.323402	0.1496333
L6 IT	GO:0042995	cell projection	76.7766885	1.67228959333333e-77	1.229564	0.2419388
L6 IT	GO:0043230	extracellular organelle	72.9546486	1.11007271941176e-73	1.235619	0.2241904
L6 IT	GO:1903561	extracellular vesicle	72.8735572	1.337958952e-73	1.235655	0.2239136
L6 IT	GO:0070062	extracellular exosome	72.3939262	4.03713985888889e-73	1.236158	0.2220454
L6 IT	GO:0044430	cytoskeletal part	64.9898329	1.02368687473684e-65	1.302871	0.1420911
L6 IT	GO:0005794	Golgi apparatus	61.6767187	2.10514161846154e-62	1.288797	0.1455162
L6 IT	GO:0098590	plasma membrane region	61.3699998	4.26579692e-62	1.295162	0.140292
L6 IT	GO:0044456	synapse part	56.2810075	5.23591368837209e-57	1.314145	0.1183919
L6 IT	GO:0048471	perinuclear region of cytoplasm	53.1200307	7.58523885777778e-54	1.411752	0.07486853
NP	GO:0005856	cytoskeleton	50.1109642	7.74525697647059e-51	1.403309	0.2108272
NP	GO:0043234	protein complex	39.8482626	1.41819986e-40	1.272822	0.290742
NP	GO:0005654	nucleoplasm	39.1185563	7.611034431e-40	1.297365	0.2568931
NP	GO:0044430	cytoskeletal part	36.3003467	5.00787298e-37	1.415162	0.1543376
NP	GO:0070062	extracellular exosome	36.1234451	7.52583832e-37	1.304116	0.2342524
NP	GO:0031090	organelle membrane	34.5155923	3.05075782846154e-35	1.311815	0.2182246
NP	GO:1903561	extracellular vesicle	34.5155923	3.05075782846154e-35	1.295183	0.2347007
NP	GO:0043230	extracellular organelle	34.4950817	3.19829333185185e-35	1.294782	0.2349249
NP	GO:0098588	bounding membrane of organelle	29.4627376	3.445580875e-30	1.359315	0.1572517
NP	GO:0015629	actin cytoskeleton	26.6127921	2.43897803032258e-27	1.653559	0.06175745
NP	GO:0005783	endoplasmic reticulum	25.7831862	1.64745606666667e-26	1.331166	0.1571397
NP	GO:0005794	Golgi apparatus	24.2159873	6.08152776e-25	1.329203	0.1500785
NP	GO:0098805	whole membrane	22.6349294	2.31777154756757e-23	1.346305	0.1314728
NP	GO:0031410	cytoplasmic vesicle	21.3258225	4.72255994736842e-22	1.282197	0.1678996
NP	GO:0097708	intracellular vesicle	21.2360065	5.80755757435897e-22	1.280894	0.1683479
NP	GO:0015630	microtubule cytoskeleton	19.3565706	4.39976422439024e-20	1.344148	0.115445
NP	GO:0048471	perinuclear region of cytoplasm	18.5974941	2.52642192e-19	1.443502	0.07655234
NP	GO:0005815	microtubule organizing center	17.1991978	6.32123833478261e-18	1.450496	0.06949115
NP	GO:0030054	cell junction	17.181286	6.58740013191489e-18	1.245624	0.1707016
NP	GO:0005739	mitochondrion	16.3875671	4.09668842916667e-17	1.277795	0.1367406
Pvalb	GO:0005856	cytoskeleton	218.311882	4.87660438222222e-219	1.445123	0.2171092
Pvalb	GO:0005654	nucleoplasm	173.249156	5.63435366421053e-174	1.33027	0.2634087
Pvalb	GO:0031090	organelle membrane	170.287915	5.153297919e-171	1.36654	0.2273283
Pvalb	GO:0044430	cytoskeletal part	152.194474	6.39036321e-153	1.448641	0.1579888
Pvalb	GO:0043234	protein complex	151.075131	8.41140969565217e-152	1.279552	0.2922792
Pvalb	GO:0042995	cell projection	132.539316	2.88857492e-133	1.288813	0.2535971
Pvalb	GO:0098588	bounding membrane of organelle	120.774412	1.68107738615385e-121	1.38357	0.1600577
Pvalb	GO:0005739	mitochondrion	120.66293	2.17305127925926e-121	1.401655	0.1499953
Pvalb	GO:0098805	whole membrane	120.090561	8.1178174e-121	1.422988	0.1389612
Pvalb	GO:0070062	extracellular exosome	118.727502	1.8728279862069e-119	1.289477	0.2316228
Pvalb	GO:0043230	extracellular organelle	115.321558	4.769163938e-116	1.283321	0.2328454
Pvalb	GO:0015629	actin cytoskeleton	115.233407	5.84242567096774e-116	1.719761	0.06422996

Table S8. The results of Gene Ontology (GO) Cellular Components enrichment analysis using GREAT.

cluster	ID	name	score	Binom_Adjp_BH	Binom_Fold_Enrichment	Binom_Region_Set_Coverage
Pvalb	GO:1903561	extracellular vesicle	115.083055	8.25932463125e-116	1.283214	0.2325319
Pvalb	GO:0031410	cytoplasmic vesicle	114.870036	1.34885058e-115	1.346552	0.1763268
Pvalb	GO:0097708	intracellular vesicle	114.502194	3.14634442294118e-115	1.34518	0.176797
Pvalb	GO:0097458	neuron part	113.601626	2.5024978e-114	1.304946	0.2082066
Pvalb	GO:0015630	microtubule cytoskeleton	111.45734	3.48867347888889e-112	1.438382	0.1235384
Pvalb	GO:0099512	supramolecular fiber	97.8163935	1.5261826195e-98	1.483957	0.09529482
Pvalb	GO:0099081	supramolecular polymer	96.8678409	1.35568588878049e-97	1.479095	0.09579637
Pvalb	GO:0099080	supramolecular complex	95.8470567	1.42214325e-96	1.47556	0.09585906
Sst	GO:0005856	cytoskeleton	95.2968694	5.04813129333333e-96	1.437004	0.2158895
Sst	GO:0043234	protein complex	66.9764569	1.05570614947368e-67	1.276908	0.2916753
Sst	GO:0042995	cell projection	63.604732	2.48466603e-64	1.298006	0.2554059
Sst	GO:0044430	cytoskeletal part	61.9099999	1.23026911333333e-62	1.424641	0.1553713
Sst	GO:0031090	organelle membrane	60.7748638	1.67933073e-61	1.324356	0.2203109
Sst	GO:0005654	nucleoplasm	58.5226703	3.00144024782609e-59	1.284272	0.2543005
Sst	GO:0097458	neuron part	55.9251246	1.18816117538462e-56	1.318892	0.2104318
Sst	GO:0015629	actin cytoskeleton	51.595245	2.53953939555556e-52	1.716563	0.06411054
Sst	GO:0005739	mitochondrion	47.6592763	2.19141038827586e-48	1.374423	0.1470812
Sst	GO:0098588	bounding membrane of organelle	44.1813533	6.58637928666667e-45	1.343658	0.1554404
Sst	GO:0098805	whole membrane	42.6926456	2.0293378875e-43	1.37314	0.1340933
Sst	GO:0015630	microtubule cytoskeleton	41.8475703	1.42046212e-42	1.397988	0.1200691
Sst	GO:0044463	cell projection part	41.6853425	2.06375187117647e-42	1.353325	0.1418998
Sst	GO:0048471	perinuclear region of cytoplasm	38.303984	4.9661062e-39	1.500697	0.07958549
Sst	GO:0070062	extracellular exosome	36.9964981	1.00809610611111e-37	1.239194	0.2225907
Sst	GO:0030054	cell junction	36.1184459	7.6129697368421e-37	1.279447	0.1753368
Sst	GO:0099512	supramolecular fiber	35.757569	1.74755559282051e-36	1.432972	0.09202073
Sst	GO:1903561	extracellular vesicle	35.546963	2.8381610565e-36	1.232929	0.2234197
Sst	GO:0043230	extracellular organelle	35.4893759	3.24059018585366e-36	1.232514	0.2236269
Sst	GO:0099081	supramolecular polymer	35.2073471	6.2037305e-36	1.427199	0.09243523
Vip	GO:0005654	nucleoplasm	85.8072505	1.55865338588235e-86	1.356394	0.2685815
Vip	GO:0005856	cytoskeleton	81.1116289	7.73341135368421e-82	1.413647	0.2123803
Vip	GO:0043234	protein complex	73.1216017	7.557850454e-74	1.298027	0.2964993
Vip	GO:0044430	cytoskeletal part	64.8795314	1.3196799e-65	1.448131	0.1579332
Vip	GO:0031090	organelle membrane	55.110151	7.75977208695652e-56	1.31754	0.2191771
Vip	GO:0070062	extracellular exosome	49.9289139	1.17783950307692e-50	1.287323	0.2312358
Vip	GO:0042995	cell projection	49.5980074	2.52343761185185e-50	1.269882	0.2498721
Vip	GO:1903561	extracellular vesicle	48.4654641	3.42401686e-49	1.281305	0.2321859
Vip	GO:0043230	extracellular organelle	48.4194179	3.80699337586207e-49	1.280895	0.2324052
Vip	GO:0048471	perinuclear region of cytoplasm	45.9243871	1.190180684e-46	1.568267	0.0831689
Vip	GO:0005739	mitochondrion	45.7695653	1.69994430064516e-46	1.377489	0.1474092
Vip	GO:0098805	whole membrane	44.5213441	3.010619679375e-45	1.392749	0.1360082
Vip	GO:0015629	actin cytoskeleton	44.4029186	3.95440738e-45	1.680901	0.06277863
Vip	GO:0098588	bounding membrane of organelle	43.9303056	1.174071228e-44	1.352571	0.1564715
Vip	GO:0015630	microtubule cytoskeleton	43.4110137	3.88138126444444e-44	1.41764	0.1217569
Vip	GO:0031410	cytoplasmic vesicle	42.9421166	1.14257140918919e-43	1.323291	0.1732807
Vip	GO:0097708	intracellular vesicle	42.7455441	1.79661850789474e-43	1.321763	0.1737192
Vip	GO:0005794	Golgi apparatus	37.2562942	5.5425018e-38	1.328217	0.1499671
Vip	GO:0030054	cell junction	33.9123545	1.22361701e-34	1.278309	0.1751809
Vip	GO:0044463	cell projection part	31.8195562	1.5151088126087e-32	1.315954	0.1379814

Table S9. The results of Gene Ontology (GO) Molecular Function enrichment analysis using GREAT.

cluster	ID	name	score	Binom_Adjp_BH	Binom_Fold_Enrichment	Binom_Region_Set_Coverage
L2/3 IT	GO:0043168	anion binding	307.652656	5.18408260211845e-319	1.296056	0.2657639
L2/3 IT	GO:0019899	enzyme binding	260.696435	2.0117068065e-261	1.324414	0.2006483
L2/3 IT	GO:0017076	purine nucleotide binding	239.692263	2.03112549e-240	1.327267	0.1847007
L2/3 IT	GO:0032555	purine ribonucleotide binding	237.790854	1.6186257702e-238	1.326198	0.1842685
L2/3 IT	GO:0001883	purine nucleoside binding	233.804864	1.56724093718182e-234	1.328108	0.1801628
L2/3 IT	GO:0001882	nucleoside binding	233.747312	1.7893213065e-234	1.327556	0.1805229
L2/3 IT	GO:0032549	ribonucleoside binding	233.635243	2.31609980630769e-234	1.327901	0.1801628
L2/3 IT	GO:0032550	purine ribonucleoside binding	233.476571	3.33756178071429e-234	1.327927	0.1800187
L2/3 IT	GO:0032553	ribonucleotide binding	233.362284	4.342262094e-234	1.321202	0.1851761
L2/3 IT	GO:0036094	small molecule binding	224.31684	4.82124879e-225	1.277467	0.2191313
L2/3 IT	GO:0035639	purine ribonucleoside triphosphate binding	223.508974	3.09760119e-224	1.321819	0.1780307
L2/3 IT	GO:0000166	nucleotide binding	222.276282	5.293194885e-223	1.29598	0.1990348
L2/3 IT	GO:0097367	carbohydrate derivative binding	215.659505	2.1902562e-216	1.274825	0.2149679
L2/3 IT	GO:0098772	molecular function regulator	213.090841	8.112573468e-214	1.358346	0.1467694
L2/3 IT	GO:0030554	adenyl nucleotide binding	196.729334	1.86494447442857e-197	1.324569	0.1576172
L2/3 IT	GO:0032559	adenyl ribonucleotide binding	194.826855	1.48985773077273e-195	1.323221	0.1572139
L2/3 IT	GO:0005524	ATP binding	179.813004	1.53813887491304e-180	1.316263	0.1512785
L2/3 IT	GO:0016740	transferase activity	168.039024	9.14062755375e-169	1.233802	0.2193618
L2/3 IT	GO:0008092	cytoskeletal protein binding	167.478116	3.3257094696e-168	1.392934	0.1034935
L2/3 IT	GO:0019900	kinase binding	136.101335	7.91890127035714e-137	1.423211	0.07655406
L4	GO:0043168	anion binding	104.090807	8.1132088158e-105	1.262561	0.2588955
L4	GO:0019899	enzyme binding	79.6455951	2.261543317875e-80	1.277727	0.1935752
L4	GO:0098772	molecular function regulator	70.0749894	8.414157123e-71	1.318846	0.1425015
L4	GO:0036094	small molecule binding	69.9148603	1.21657724672727e-70	1.240363	0.2127667
L4	GO:0017076	purine nucleotide binding	68.7904858	1.6199971245e-69	1.271268	0.1769079
L4	GO:0032555	purine ribonucleotide binding	68.6807193	2.08583881961538e-69	1.271232	0.1766313
L4	GO:0032553	ribonucleotide binding	67.5099259	3.09082305214286e-68	1.26739	0.1776341
L4	GO:0032549	ribonucleoside binding	67.4620677	3.4508997e-68	1.272562	0.1726547
L4	GO:0001882	nucleoside binding	67.2650162	5.43230114625e-68	1.271727	0.1729313
L4	GO:0001883	purine nucleoside binding	67.2286374	5.9069412e-68	1.271996	0.1725509
L4	GO:0032550	purine ribonucleoside binding	66.8676185	1.356380343e-67	1.271309	0.1723434
L4	GO:0097367	carbohydrate derivative binding	66.0884322	8.15770094684211e-67	1.235728	0.2083751
L4	GO:0016740	transferase activity	64.9312195	1.17160294242857e-65	1.225882	0.2179536
L4	GO:0000166	nucleotide binding	61.7642764	1.72077293209091e-62	1.241292	0.1906359
L4	GO:0035639	purine ribonucleoside triphosphate binding	61.6420889	2.27987555086957e-62	1.261108	0.1698537
L4	GO:0008289	lipid binding	59.6698107	2.1388941e-60	1.417186	0.08264463
L4	GO:0032559	adenyl ribonucleotide binding	56.4059379	3.92701122e-57	1.268951	0.1507659
L4	GO:0030554	adenyl nucleotide binding	56.3164326	4.82577859038462e-57	1.268446	0.1509388
L4	GO:0008092	cytoskeletal protein binding	56.2217985	6.00069366e-57	1.352477	0.1004876
L4	GO:0005524	ATP binding	50.0341727	9.24330577178571e-51	1.257946	0.1445762
L5 IT	GO:0043168	anion binding	113.444868	3.5903132388e-114	1.265472	0.2594924
L5 IT	GO:0019899	enzyme binding	95.9847951	1.035630684e-96	1.295758	0.1963069
L5 IT	GO:0098772	molecular function regulator	85.5404323	2.88116240914286e-86	1.341961	0.144999
L5 IT	GO:0097367	carbohydrate derivative binding	73.7962808	1.598524014375e-74	1.241911	0.2094178
L5 IT	GO:0017076	purine nucleotide binding	71.7826418	1.6495223454e-72	1.268413	0.1765107
L5 IT	GO:0032555	purine ribonucleotide binding	71.6122681	2.44192235318182e-72	1.268262	0.1762186
L5 IT	GO:0016740	transferase activity	71.5632434	2.73373641225e-72	1.230076	0.2186993
L5 IT	GO:0032553	ribonucleotide binding	71.5293072	2.95592084376923e-72	1.266553	0.1775167
L5 IT	GO:0032549	ribonucleoside binding	70.9102447	1.22957592107143e-71	1.270605	0.1723892
L5 IT	GO:0032550	purine ribonucleoside binding	70.5449178	2.851557696e-71	1.269971	0.172162

Table S10. The results of Gene Ontology (GO) Molecular Function enrichment analysis using GREAT.

cluster	ID	name	score	Binom_Adjp_BH	Binom_Fold_Enrichment	Binom_Region_Set_Coverage
L5 IT	GO:0001883	purine nucleoside binding	70.4419957	3.61413444375e-71	1.269607	0.1722269
L5 IT	GO:0001882	nucleoside binding	70.3246512	4.73531427e-71	1.268934	0.1725514
L5 IT	GO:0036094	small molecule binding	69.5173798	3.03822657e-70	1.231624	0.2112676
L5 IT	GO:0000166	nucleotide binding	68.7849307	1.64085158295e-69	1.246733	0.1914714
L5 IT	GO:0035639	purine ribonucleoside triphosphate binding	67.0310747	9.30947799857143e-68	1.263789	0.1702148
L5 IT	GO:0008092	cytoskeletal protein binding	60.5702588	2.6899316325e-61	1.354476	0.1006361
L5 IT	GO:0030554	adenyl nucleotide binding	57.6696482	2.1396944124e-58	1.262984	0.1502888
L5 IT	GO:0032559	adenyl ribonucleotide binding	57.3653179	4.31203313076923e-58	1.262477	0.1499968
L5 IT	GO:0005524	ATP binding	53.2401472	5.75244963e-54	1.257672	0.1445447
L5 IT	GO:0008289	lipid binding	51.594964	2.54118317464286e-52	1.373996	0.08012592
L5 PT	GO:0043168	anion binding	237.839026	1.448683866e-238	1.328968	0.2725126
L5 PT	GO:0019899	enzyme binding	199.03081	9.31514337e-200	1.364738	0.2067573
L5 PT	GO:0017076	purine nucleotide binding	185.204697	6.24169904142857e-186	1.370394	0.1907022
L5 PT	GO:0032555	purine ribonucleotide binding	183.844437	1.430748613125e-184	1.369273	0.1902536
L5 PT	GO:0032553	ribonucleotide binding	181.204208	6.24872961e-182	1.364504	0.1912452
L5 PT	GO:0032549	ribonucleoside binding	180.544704	2.8529652654e-181	1.371126	0.1860273
L5 PT	GO:0032550	purine ribonucleoside binding	180.48448	3.27732809890909e-181	1.371205	0.1858856
L5 PT	GO:0001883	purine nucleoside binding	179.975042	1.0591519095e-180	1.37047	0.1859092
L5 PT	GO:0001882	nucleoside binding	179.869481	1.35057738253846e-180	1.369771	0.1862634
L5 PT	GO:0000166	nucleotide binding	178.026257	9.41333330571428e-179	1.341037	0.2059546
L5 PT	GO:0035639	purine ribonucleoside triphosphate binding	176.954121	1.1114218656e-177	1.368745	0.1843509
L5 PT	GO:0036094	small molecule binding	169.006738	9.84604572e-170	1.309526	0.2246305
L5 PT	GO:0030554	adenyl nucleotide binding	158.863742	1.36854050921053e-159	1.375816	0.1637154
L5 PT	GO:0097367	carbohydrate derivative binding	157.665743	2.1590237745e-158	1.301883	0.2195306
L5 PT	GO:0032559	adenyl ribonucleotide binding	157.125642	7.48787027142857e-158	1.373968	0.1632431
L5 PT	GO:0098772	molecular function regulator	156.98746	1.02929535736364e-157	1.395652	0.1508004
L5 PT	GO:0005524	ATP binding	148.201485	6.28803397956522e-149	1.370034	0.1574586
L5 PT	GO:0008092	cytoskeletal protein binding	141.990416	1.022313544875e-142	1.467497	0.1090334
L5 PT	GO:0016740	transferase activity	125.899827	1.25942689296e-126	1.259983	0.2240166
L5 PT	GO:0019900	kinase binding	99.4700031	3.38841756e-100	1.466065	0.07885914
L6 CT	GO:0043168	anion binding	110.474591	3.3528071772e-111	1.290982	0.2647235
L6 CT	GO:0017076	purine nucleotide binding	90.1861568	6.51393120375e-91	1.335454	0.18584
L6 CT	GO:0032555	purine ribonucleotide binding	89.7961109	1.599149574e-90	1.334928	0.1854816
L6 CT	GO:0001882	nucleoside binding	89.2585052	5.514356232e-90	1.338252	0.1819775
L6 CT	GO:0001883	purine nucleoside binding	88.5944647	2.54410639363636e-89	1.337376	0.18142
L6 CT	GO:0032553	ribonucleotide binding	88.2296926	5.8926064275e-89	1.329916	0.1863975
L6 CT	GO:0032550	purine ribonucleoside binding	87.7010768	1.99032151984615e-88	1.33562	0.1810616
L6 CT	GO:0032549	ribonucleoside binding	87.5610012	2.74788631671429e-88	1.335113	0.1811412
L6 CT	GO:0000166	nucleotide binding	87.0046224	9.89413056e-88	1.309369	0.2010911
L6 CT	GO:0035639	purine ribonucleoside triphosphate binding	86.8827534	1.3099256415e-87	1.335158	0.1798272
L6 CT	GO:0036094	small molecule binding	86.7932034	1.609891524e-87	1.288135	0.2209613
L6 CT	GO:0097367	carbohydrate derivative binding	81.7676504	1.7074563615e-82	1.282502	0.2162625
L6 CT	GO:0019899	enzyme binding	78.7601237	1.73730596494737e-79	1.296322	0.1963923
L6 CT	GO:0098772	molecular function regulator	77.9759725	1.05688433475e-78	1.361729	0.147135
L6 CT	GO:0016740	transferase activity	72.8148981	1.53144676104545e-73	1.257356	0.2235496
L6 CT	GO:0030554	adenyl nucleotide binding	71.3705916	4.25998778086956e-72	1.326162	0.1578067
L6 CT	GO:0032559	adenyl ribonucleotide binding	70.708068	1.9585380825e-71	1.324859	0.1574085
L6 CT	GO:0005524	ATP binding	66.9409841	1.14555488652e-67	1.322133	0.1519532
L6 CT	GO:0019900	kinase binding	63.8566627	1.391032548e-64	1.487245	0.07999841
L6 CT	GO:0008047	enzyme activator activity	59.6867469	2.05708898892857e-60	1.545949	0.06379166

Table S11. The results of Gene Ontology (GO) Molecular Function enrichment analysis using GREAT.

cluster	ID	name	score	Binom_Adjp_BH	Binom_Fold_Enrichment	Binom_Region_Set_Coverage
L6 IT	GO:0043168	anion binding	103.888787	1.2918529296e-104	1.262373	0.2588569
L6 IT	GO:0016740	transferase activity	82.320222	4.783854461625e-83	1.255512	0.2232217
L6 IT	GO:0017076	purine nucleotide binding	81.7687037	1.703320083e-82	1.296541	0.1804249
L6 IT	GO:0032555	purine ribonucleotide binding	81.2622865	5.4665523e-82	1.295795	0.1800443
L6 IT	GO:0032553	ribonucleotide binding	80.2290402	5.90146398e-81	1.292239	0.1811168
L6 IT	GO:0098772	molecular function regulator	79.6504639	2.23633096425e-80	1.340343	0.1448242
L6 IT	GO:0001882	nucleoside binding	79.5767385	2.65009528730769e-80	1.296303	0.1762732
L6 IT	GO:0001883	purine nucleoside binding	79.2843766	5.19545247428571e-80	1.29612	0.1758234
L6 IT	GO:0032549	ribonucleoside binding	79.2231908	5.981486958e-80	1.295918	0.1758234
L6 IT	GO:0032550	purine ribonucleoside binding	79.0620506	8.66860916625e-80	1.295704	0.1756504
L6 IT	GO:0097367	carbohydrate derivative binding	75.5133244	3.06673047e-76	1.252372	0.2111818
L6 IT	GO:0000166	nucleotide binding	74.8394128	1.447395453e-75	1.266266	0.1944714
L6 IT	GO:0019899	enzyme binding	74.7658823	1.71442174689474e-75	1.268342	0.1921533
L6 IT	GO:0035639	purine ribonucleoside triphosphate binding	74.582118	2.617471827e-75	1.287964	0.1734708
L6 IT	GO:0036094	small molecule binding	71.5801159	2.62956641142857e-72	1.242822	0.2131885
L6 IT	GO:0030554	adenyl nucleotide binding	68.6892298	2.04536227304348e-69	1.297307	0.1543731
L6 IT	GO:0032559	adenyl ribonucleotide binding	68.1762086	6.664865895e-69	1.296399	0.1540271
L6 IT	GO:0016772	transferase activity, transferring phosphorus-containing groups	61.8418242	1.43938101288462e-62	1.367112	0.1031691
L6 IT	GO:0005524	ATP binding	61.2188937	6.04096479e-62	1.285993	0.1477996
L6 IT	GO:0016301	kinase activity	54.5605535	2.75072077285714e-55	1.363404	0.09344727
NP	GO:0043168	anion binding	36.2500211	5.62313962542857e-37	1.282858	0.2630576
NP	GO:0019899	enzyme binding	33.831952	1.47247532775e-34	1.331676	0.2017485
NP	GO:0001883	purine nucleoside binding	30.6255356	2.368451016e-31	1.337683	0.1814616
NP	GO:0000166	nucleotide binding	30.5563081	2.777742234e-31	1.31219	0.2015243
NP	GO:0001882	nucleoside binding	30.5159238	3.04843014e-31	1.336107	0.1816857
NP	GO:0035639	purine ribonucleoside triphosphate binding	29.8779639	1.32445171285714e-30	1.333978	0.1796682
NP	GO:0017076	purine nucleotide binding	29.8779639	1.32445171285714e-30	1.327348	0.1847119
NP	GO:0016740	transferase activity	29.8779639	1.32445171285714e-30	1.280991	0.2277516
NP	GO:0032550	purine ribonucleoside binding	29.7851584	1.6399915776e-30	1.331956	0.1805649
NP	GO:0032549	ribonucleoside binding	29.6471572	2.2534235791875e-30	1.330865	0.1805649
NP	GO:0032555	purine ribonucleotide binding	29.542624	2.86665885e-30	1.325356	0.1841515
NP	GO:0032553	ribonucleotide binding	28.8155078	1.5292982655e-29	1.319489	0.1849361
NP	GO:0098772	molecular function regulator	28.6183292	2.40807948157895e-29	1.372374	0.1482851
NP	GO:0036094	small molecule binding	27.7235211	1.8900746064e-28	1.276102	0.2188971
NP	GO:0030554	adenyl nucleotide binding	25.0107714	9.75502961217391e-26	1.328091	0.1580363
NP	GO:0032559	adenyl ribonucleotide binding	24.5778596	2.64326338125e-25	1.325427	0.1574759
NP	GO:0005524	ATP binding	24.3370785	4.6017340596e-25	1.330201	0.1528805
NP	GO:0097367	carbohydrate derivative binding	23.1840586	6.54547893576923e-24	1.254922	0.2116117
NP	GO:0044877	macromolecular complex binding	22.3866756	4.10510628e-23	1.305131	0.1594934
NP	GO:0019900	kinase binding	22.319511	4.79169296035714e-23	1.489858	0.08013898
Pvalb	GO:0043168	anion binding	205.938961	1.1509030719e-206	1.353821	0.2776089
Pvalb	GO:0019899	enzyme binding	183.523155	2.99809449514286e-184	1.405341	0.2129087
Pvalb	GO:0008092	cytoskeletal protein binding	168.366858	4.296766014e-169	1.59564	0.1185543
Pvalb	GO:0098772	molecular function regulator	153.333934	4.635172026e-154	1.454057	0.1571111
Pvalb	GO:0036094	small molecule binding	145.446649	3.57561746918182e-146	1.332015	0.2284881
Pvalb	GO:0017076	purine nucleotide binding	137.862518	1.3724042625e-138	1.368234	0.1904016
Pvalb	GO:0001883	purine nucleoside binding	137.230692	5.87905920642857e-138	1.373083	0.1862638
Pvalb	GO:0001882	nucleoside binding	137.230692	5.87905920642857e-138	1.37254	0.1866399
Pvalb	GO:0035639	purine ribonucleoside triphosphate binding	136.998061	1.0044751123125e-137	1.374336	0.1851039
Pvalb	GO:0032549	ribonucleoside binding	136.998061	1.0044751123125e-137	1.372638	0.1862324

Table S12. The results of Gene Ontology (GO) Molecular Function enrichment analysis using GREAT.

cluster	ID	name	score	Binom_Adjp_BH	Binom_Fold_Enrichment	Binom_Region_Set_Coverage
Pvalb	GO:0032550	purine ribonucleoside binding	136.705162	1.971686205e-137	1.372375	0.1860443
Pvalb	GO:0032555	purine ribonucleotide binding	136.700256	1.9940848785e-137	1.366728	0.1899
Pvalb	GO:0032553	ribonucleotide binding	136.385482	4.11640235052632e-137	1.364299	0.1912166
Pvalb	GO:0000166	nucleotide binding	136.33258	4.6496491155e-137	1.344067	0.2064199
Pvalb	GO:0097367	carbohydrate derivative binding	121.83734	1.45431914014286e-122	1.306113	0.2202439
Pvalb	GO:0030554	adenyl nucleotide binding	117.799948	1.58508253840909e-118	1.373002	0.1633805
Pvalb	GO:0032559	adenyl ribonucleotide binding	116.814438	1.53307139517391e-117	1.371694	0.1629729
Pvalb	GO:0005524	ATP binding	116.082465	8.27055977625e-117	1.377921	0.1583649
Pvalb	GO:0008047	enzyme activator activity	99.5622715	2.739860964e-100	1.630259	0.06727062
Pvalb	GO:0044877	macromolecular complex binding	92.9461545	1.13199759276923e-93	1.324626	0.1618758
Sst	GO:0043168	anion binding	88.3215542	4.76920335375e-89	1.345267	0.2758549
Sst	GO:0019899	enzyme binding	78.1633531	6.8650998714e-79	1.394464	0.2112608
Sst	GO:0098772	molecular function regulator	65.8817347	1.31300163e-66	1.443711	0.1559931
Sst	GO:0036094	small molecule binding	57.6998111	1.9961301825e-58	1.31173	0.2250086
Sst	GO:0008092	cytoskeletal protein binding	57.155787	6.98575008272727e-58	1.513748	0.1124698
Sst	GO:0017076	purine nucleotide binding	56.4993858	3.16675334025e-57	1.351324	0.1880484
Sst	GO:0001883	purine nucleoside binding	56.0069656	9.84089044384615e-57	1.355173	0.1838342
Sst	GO:0001882	nucleoside binding	55.8199272	1.51381498242857e-56	1.353939	0.1841105
Sst	GO:0032555	purine ribonucleotide binding	55.4558266	3.500849514375e-56	1.347932	0.1872884
Sst	GO:0032553	ribonucleotide binding	55.4558266	3.500849514375e-56	1.346131	0.1886701
Sst	GO:0032549	ribonucleoside binding	55.4408855	3.62338515e-56	1.352925	0.1835579
Sst	GO:0032550	purine ribonucleoside binding	55.4387031	3.641639445e-56	1.353014	0.1834197
Sst	GO:0000166	nucleotide binding	55.0851916	8.21880061105263e-56	1.325656	0.2035924
Sst	GO:0035639	purine ribonucleoside triphosphate binding	53.994082	1.01371992885e-54	1.349521	0.1817617
Sst	GO:0030554	adenyl nucleotide binding	48.3946635	4.03029212142857e-49	1.356205	0.1613817
Sst	GO:0032559	adenyl ribonucleotide binding	46.8871175	1.29682849377273e-47	1.350741	0.1604836
Sst	GO:0097367	carbohydrate derivative binding	46.356447	4.40101652869565e-47	1.281109	0.2160276
Sst	GO:0005524	ATP binding	44.8125579	1.539721294875e-45	1.349469	0.155095
Sst	GO:0019900	kinase binding	42.9394299	1.14966176148e-43	1.530942	0.08234888
Sst	GO:0008047	enzyme activator activity	37.8120649	1.54146993923077e-38	1.577116	0.06507772
Vip	GO:0019899	enzyme binding	92.241147	5.739221709e-93	1.442857	0.2185924
Vip	GO:0043168	anion binding	76.8319444	1.4725011465e-77	1.330825	0.2728934
Vip	GO:0098772	molecular function regulator	63.6010262	2.505958236e-64	1.448815	0.1565446
Vip	GO:0008092	cytoskeletal protein binding	55.3347206	4.626786096e-56	1.520708	0.1129869
Vip	GO:0001882	nucleoside binding	53.832305	1.47127893381818e-54	1.358139	0.1846817
Vip	GO:0017076	purine nucleotide binding	53.6455148	2.2619614095e-54	1.352339	0.1881897
Vip	GO:0001883	purine nucleoside binding	53.6043325	2.48695256423077e-54	1.357649	0.1841701
Vip	GO:0035639	purine ribonucleoside triphosphate binding	53.0147482	9.66611214642857e-54	1.357093	0.1827816
Vip	GO:0032553	ribonucleotide binding	52.8821964	1.3116065454e-53	1.347918	0.1889206
Vip	GO:0032555	purine ribonucleotide binding	52.7250165	1.883577472875e-53	1.349159	0.1874589
Vip	GO:0032550	purine ribonucleoside binding	52.6797087	2.090698128e-53	1.354237	0.1835855
Vip	GO:0032549	ribonucleoside binding	52.5902541	2.568892185e-53	1.353667	0.1836586
Vip	GO:0036094	small molecule binding	52.303175	4.97536552894737e-53	1.304575	0.2237813
Vip	GO:0000166	nucleotide binding	48.8339059	1.4658655212e-49	1.315304	0.2020025
Vip	GO:0097367	carbohydrate derivative binding	46.5887397	2.57786596285714e-47	1.290252	0.2175692
Vip	GO:0019900	kinase binding	44.8019413	1.57782437140909e-45	1.559774	0.08389973
Vip	GO:0044877	macromolecular complex binding	43.8717528	1.34352962021739e-44	1.343197	0.1641453
Vip	GO:0030554	adenyl nucleotide binding	42.9817713	1.04286647475e-43	1.345036	0.1600526
Vip	GO:0032559	adenyl ribonucleotide binding	42.2304427	5.882436594e-43	1.342193	0.159468
Vip	GO:0005524	ATP binding	42.0170561	9.61488025615385e-43	1.348093	0.1549368

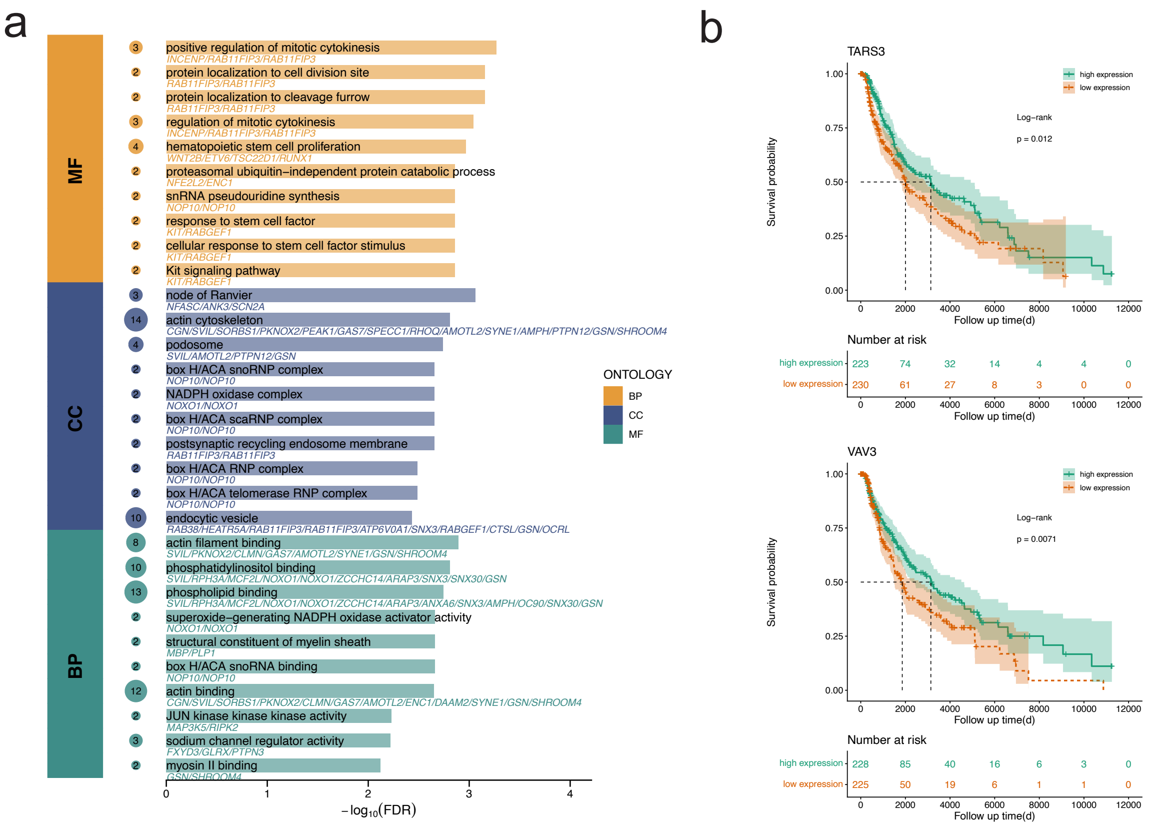

Supplementary Fig. S8. (a) Results of GO enrichment using the top 100 genes. (b) The results of survival analysis on genes TARS3, and VAV3.

Supplementary Note 1: Ablations for the Qualitative and Quantitative Decoder

This section provides systematic ablations to isolate the effect of PAIR's joint qualitative and quantitative learning via dual decoders. We evaluate the ablations on five representative datasets, buen_ad_sc, Leukemia, Buenrostro_2018, T_cell, and Forebrain. Clustering quality is measured using both ARI and NMI, and the conclusions are consistent across the two metrics.

We first test whether the improvements require jointly optimizing the qualitative and quantitative objectives by removing one decoder at a time. We consider three settings. **Qualitative only.** We remove the quantitative NB decoder and train the model using only the qualitative reconstruction objective. **Quantitative only.** We remove the qualitative decoder and train the model using only the quantitative NB objective. **Both.** We train the full model with both decoders.

Supplementary Figure 9 a&b report ARI and NMI for these three variants. Across all five datasets, the full model that uses both decoders achieves the best performance under both metrics, while either single view variant shows degraded performance. This indicates that the gains are not driven by only qualitative structure learning or only quantitative count modeling, but rather by their complementary combination.

To further disentangle the contribution of qualitative versus quantitative learning, we sweep the weighting coefficient α that controls the strength of the quantitative objective relative to the qualitative objective in the overall training loss. Supplementary Figure 9 c&d show per dataset ARI and NMI as a function of α.

The results show a clear optimum at a moderate α. When α is too small, the model underutilizes quantitative count information and clustering quality decreases. When α is too large, the quantitative term dominates optimization and performance also degrades. Supplementary Figure 9 e&f summarize the average performance across the five datasets, where the bars show the

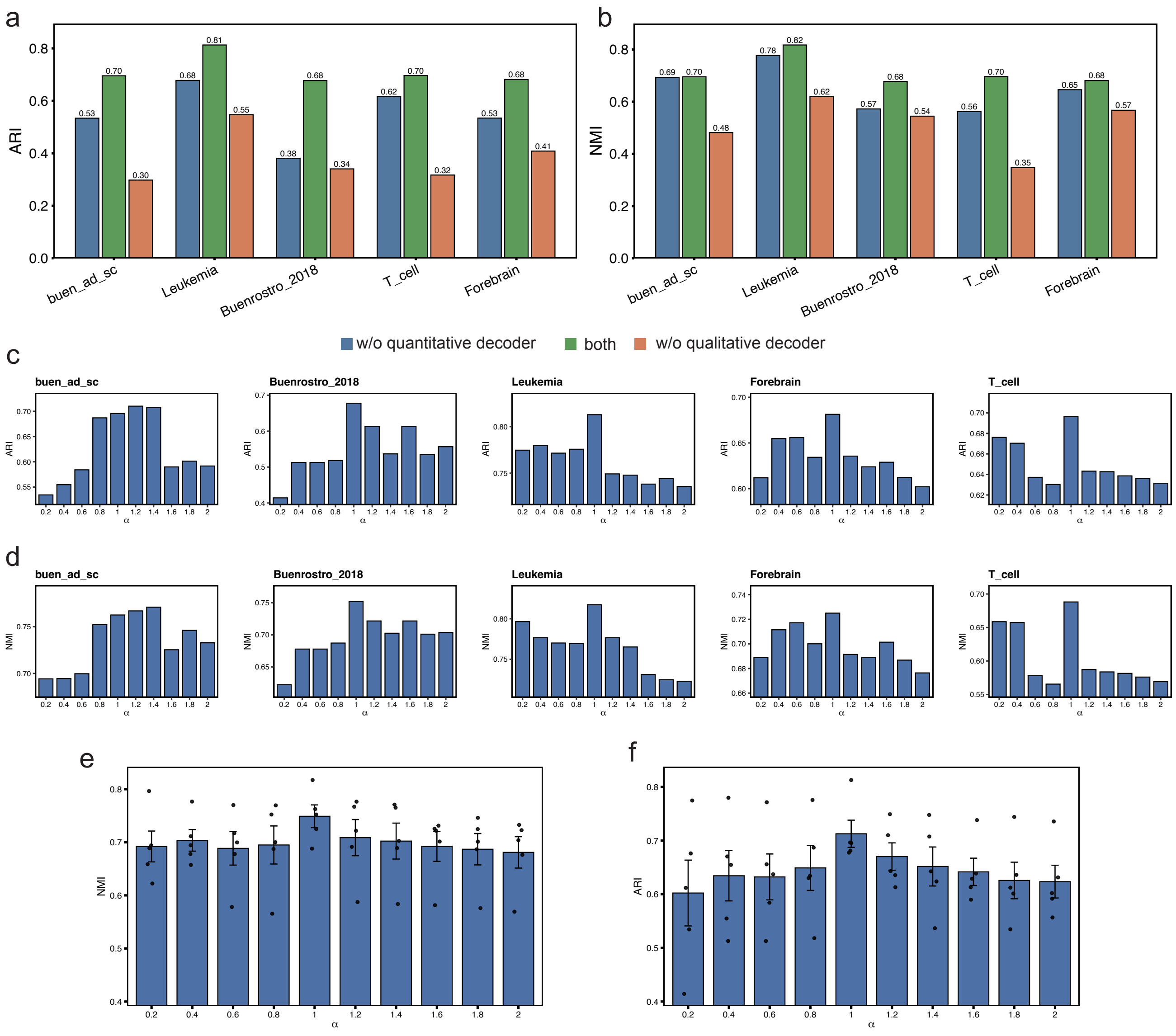

Supplementary Fig. S9. Ablations for the dual decoder design and the qualitative quantitative balance. (a) ARI of three variants, qualitative only, quantitative only, and both decoders, evaluated on five datasets. (b) NMI of the same three variants on the same five datasets. (c) ARI as a function of the balance coefficient α on each dataset. (d) NMI as a function of α on each dataset. (e) Mean NMI across the five datasets for each α, where bars denote the mean, black points denote dataset specific values, and error bars indicate the standard deviation across datasets. (f) Mean ARI across the five datasets for each α, with the same aggregation protocol.

mean and the black points show dataset specific values. These results support that the best performance is obtained when both decoders are jointly optimized with a balanced contribution, consistent with the motivation of combining robust qualitative learning under sparsity with informative quantitative count variation.

Supplementary Note 2: Ablation Studies for Hyperparameters

This section provides systematic ablations to assess the sensitivity of PAIR to key design choices and hyperparameters. Unless otherwise specified, we keep the data preprocessing, training schedule, and all remaining hyperparameters fixed, and evaluate clustering quality using both ARI and NMI. Across all ablations, ARI and NMI exhibit highly consistent trends, supporting that the conclusions are not metric dependent.

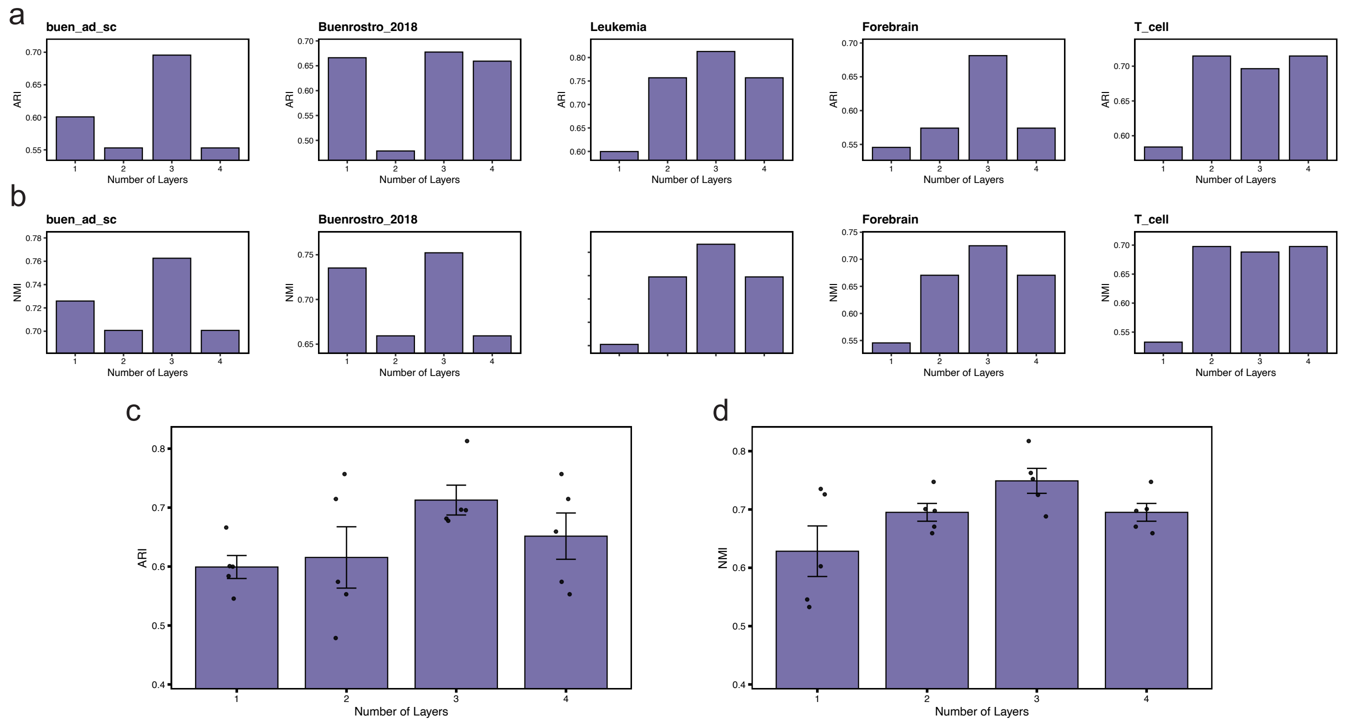

Supplementary Fig. S10. Sensitivity analysis of the number of propagation layers K on five datasets. We evaluate $K \in \{1,2,3,4\}$ on five representative datasets (buen_ad_sc, Buenrostro_2018, Leukemia, Forebrain, and T_cell) while keeping all other settings fixed. (a) ARI as a function of K for each dataset. (b) NMI as a function of K for each dataset. (c) Aggregated ARI across the five datasets: bars show the mean ARI, black dots show dataset specific ARI values, and error bars indicate the standard deviation across datasets. (d) Aggregated NMI across the five datasets: bars show the mean NMI, black dots show dataset specific NMI values, and error bars indicate the standard deviation across datasets.

A. Number of propagation layers K. We further analyze the sensitivity to the depth of graph propagation. We evaluate the number of propagation layers on the same five representative datasets (buen_ad_sc, Buenrostro_2018, Leukemia, Forebrain, and T_cell) and test $K \in \{1,2,3,4\}$ with all other settings fixed.

Supplementary Figure 10 shows that $K = 3$ provides the strongest overall clustering performance on average under both ARI and NMI. Values $K = 2$ and $K = 4$ typically yield similar but slightly lower performance, suggesting that moderate depth is sufficient to capture multi-hop relations in the cell peak graph, while deeper propagation offers diminishing returns. Based on this stability and overall performance, we use $K = 3$ as the default setting throughout the main experiments.

B. Embedding dimension d. We assess sensitivity to the embedding dimension used for node representations. Due to the computational cost of full sweeps on all datasets, we conduct a systematic evaluation on the same five representative datasets. We test $d \in \{16,32,48,64,80\}$ while keeping all other settings fixed.

Supplementary Figure 11 reports per-dataset ARI and NMI and aggregated results across the five datasets. Performance improves from very small embeddings and reaches the best or near-best values around $d = 64$ under both ARI and NMI. Nearby values such as $d = 48$ and $d = 80$ achieve comparable performance, indicating that PAIR does not rely on a narrowly tuned embedding size. We choose $d = 64$ as a robust default that provides consistently strong performance across datasets while maintaining a favorable computation accuracy tradeoff.

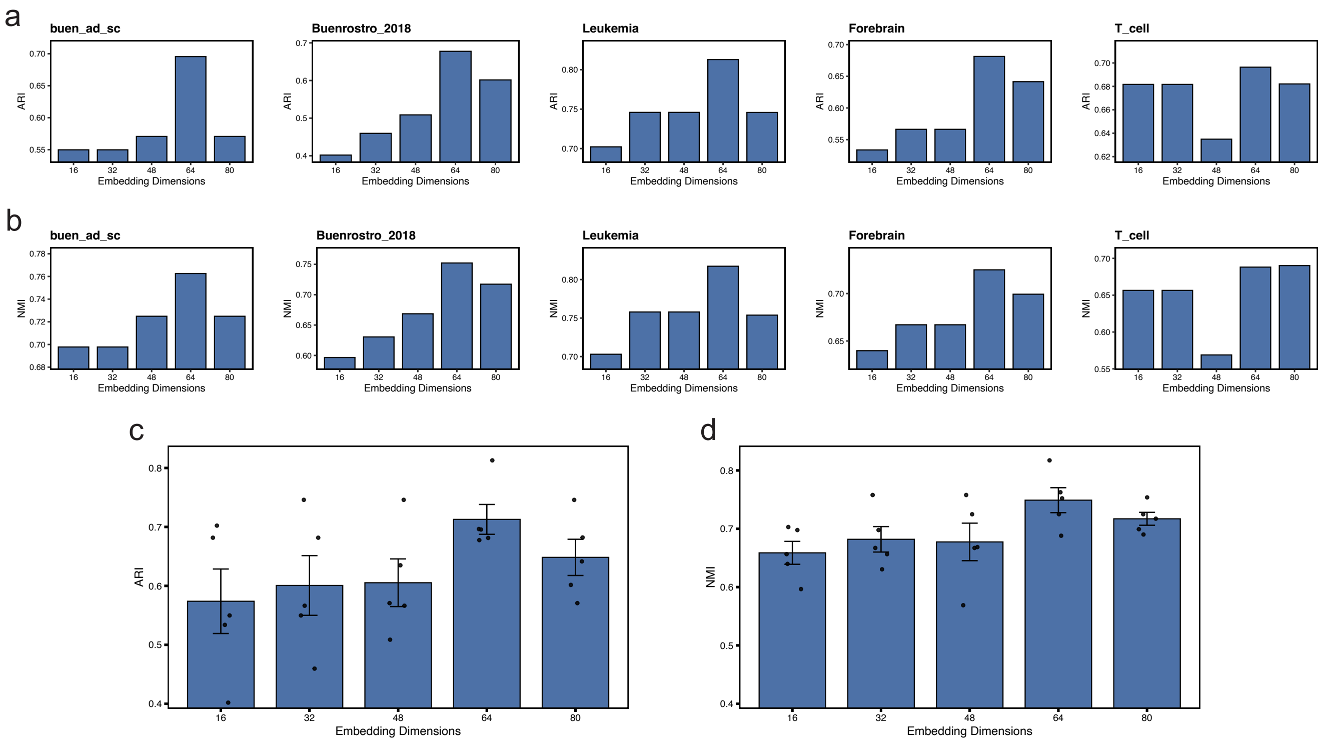

Supplementary Fig. S11. Sensitivity analysis of the embedding dimension d on five datasets. We evaluate $d \in \{16, 32, 48, 64, 80\}$ on five representative datasets (buen_ad_sc, Buenrostro_2018, Leukemia, Forebrain, and T_cell) while keeping all other settings fixed. (a) ARI as a function of d for each dataset. (b) NMI as a function of d for each dataset. (c) Aggregated ARI across the five datasets: bars show the mean ARI, black dots show dataset specific ARI values, and error bars indicate the standard deviation across datasets. (d) Aggregated NMI across the five datasets: bars show the mean NMI, black dots show dataset specific NMI values, and error bars indicate the standard deviation across datasets.
